# New benzochromene-based compounds as potential EGFR-TK inhibitors: synthesis, anti-proliferative activity, molecular docking studies, and ADME profiles

**DOI:** 10.1039/d6ra02423h

**Published:** 2026-04-30

**Authors:** Mohamed H. Hekal, Abdullah Yahya Abdullah Alzahrani, Saad Alrashdi, Fatma S. M. Abu El-Azm, Yasmeen M. Ali

**Affiliations:** a Department of Chemistry, Faculty of Science, Ain Shams University Abbassia 11566 Cairo Egypt mohamed.hekal@sci.asu.edu.eg; b Department of Chemistry, Faculty of Science, King Khalid University Abha Saudi Arabia; c Department of Chemistry, College of Science, Jouf University Sakaka 72341 Aljouf Saudi Arabia

## Abstract

In this study, we report the synthesis and biological evaluation of a novel series of benzochromene and benzochromenopyrimidine derivatives employingenaminonitrile compound 1 as a key synthetic precursor. The structures of the synthesized compounds were elucidated using comprehensive analytical and spectroscopic techniques. The antiproliferative activities of the prepared derivatives were evaluated against three cancer cell lines, HCT-116, MCF-7, and HepG2, as well as the normal human lung fibroblast cell line, WI-38, using the MTT assay, with doxorubicin used as the standard reference drug. Among the tested derivatives, compounds 6, 7, and 10 demonstrated notable antiproliferative activity against all examined cancer cell lines. In particular, compound 7 exhibited potent cytotoxic effects, with IC_50_ values of 5.98, 6.52, and 8.51 µM against HCT-116, MCF-7, and HepG2 cells, respectively, comparable with that of doxorubicin. Importantly, compound 7 displayed low cytotoxicity toward WI-38 cells, resulting in the highest selectivity index (SI = 6.7). Molecular docking analysis further revealed that compound 7 exhibited the promising binding affinity toward EGFR, with a docking score of −9.06 kcal mol^−1^, comparable with that of gefitinib. Collectively, these findings highlight compound 7 as a potent EGFR-targeting kinase inhibitor with notable anticancer activity.

## Abstract 2

New benzochromenopyrimidines were synthesized as potential EGFR inhibitors. Their antiproliferative activities were evaluated against cancer cell lines and normal fibroblasts. Compound 7 exhibited strong cytotoxicity and good selectivity.

## Graphical abstract

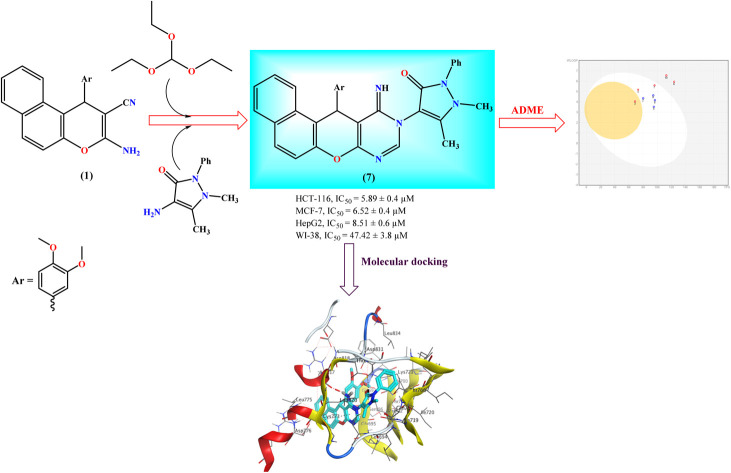

## 1. Introduction

Benzochromenes are fused oxygen-containing heterocycles generated by the annulation of a benzene ring. They have attracted considerable interest in medicinal chemistry owing to their structural diversity and wide range of biological activities. Benzochromene derivatives display antifungal and antibacterial activities,^1–4^ exhibit anti-inflammatory and analgesic effects,^5^ have antispasmolytic^6^ and antioxidant properties,^7,8^ and present anti-HIV activity^9^ due to the presence of a chromene (benzopyran) moiety. These scaffolds are widely found in natural products and present antianaphylactic,^10^ antileishmanial,^11^ estrogenic,^12^ antihelminthic,^13^ and CNS effects,^14–16^ antidepressant and anticonvulsant activities,^17^ as well as antitubercular properties.^18^

**Fig. 1:**
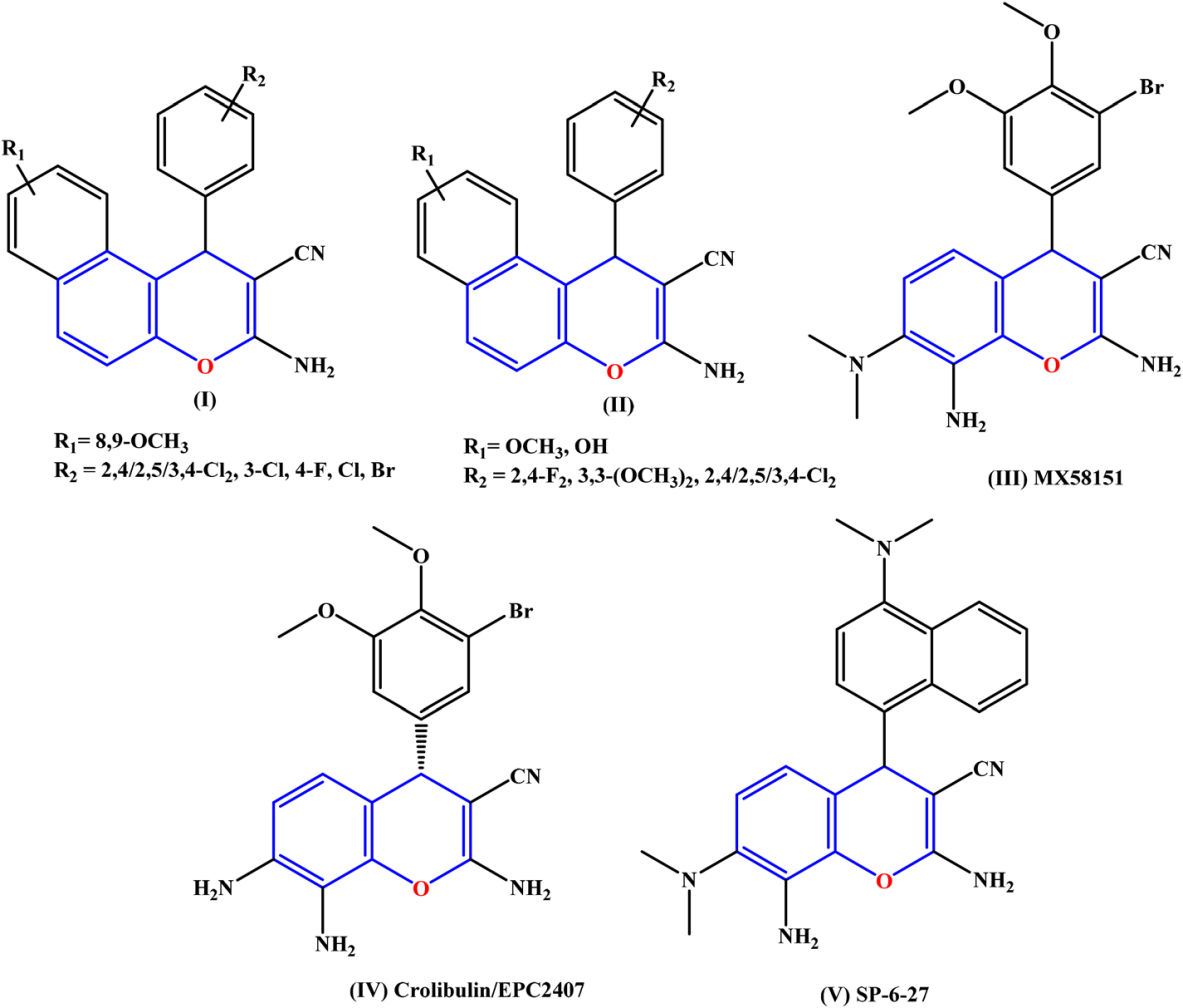
Chromene-based anticancer agents.

Their potent anticancer properties have established benzochromenes as privileged scaffolds in drug discovery against a wide range of human cancers. Many derivatives induce cell-cycle arrest and apoptosis. They can bind to DNA,^19^ inhibit key enzymes, such as topoisomerase I and II, and regulate anti-apoptotic proteins such as Bcl-2.^20^ The identification of HA14-1 marked a significant advancement in the development of 4*H*-chromene-based anticancer agents. Evidence indicates that it acts synergistically with flavopiridol to downregulate Bcl-2 by disrupting the interaction between Bax and Bcl-2.^21–23^ Certain structural motifs have gained attention as potential leads in anticancer drug development (Fig. 1). Notably, benzo[*f*]chromene-2-carbonitrile derivatives (I) induce cell-cycle arrest and apoptosis in human cancer cells through the simultaneous inhibition of tubulin and topoisomerases,^24^ while 9-hydroxy/methoxy-1*H*-benzo[*f*]chromenes (II) show activity against resistant breast cancer cells *via P*-glycoprotein inhibition and apoptosis.^25^ Moreover, chromene-based derivatives have gained considerable research attention as attractive and versatile scaffolds for the design of potent antitumour agents.^26^ Several representatives highlight their therapeutic potential; for instance, 2-amino-4-(3-bromo-4,5-dimethoxyphenyl)-7-(dimethylamino)-4*H*-chromene-3-carbonitrile (III) has been identified as an effective tubulin polymerization inhibitor.^27^ In addition, Crolibulin™ (IV) has progressed to phase I/II clinical trials for the treatment of advanced solid tumors,^28^ while SP-6-27 (V) demonstrates strong antitumour activity against both cisplatin-sensitive and cisplatin-resistant ovarian cancer cell lines.^29^

**Fig. 2:**
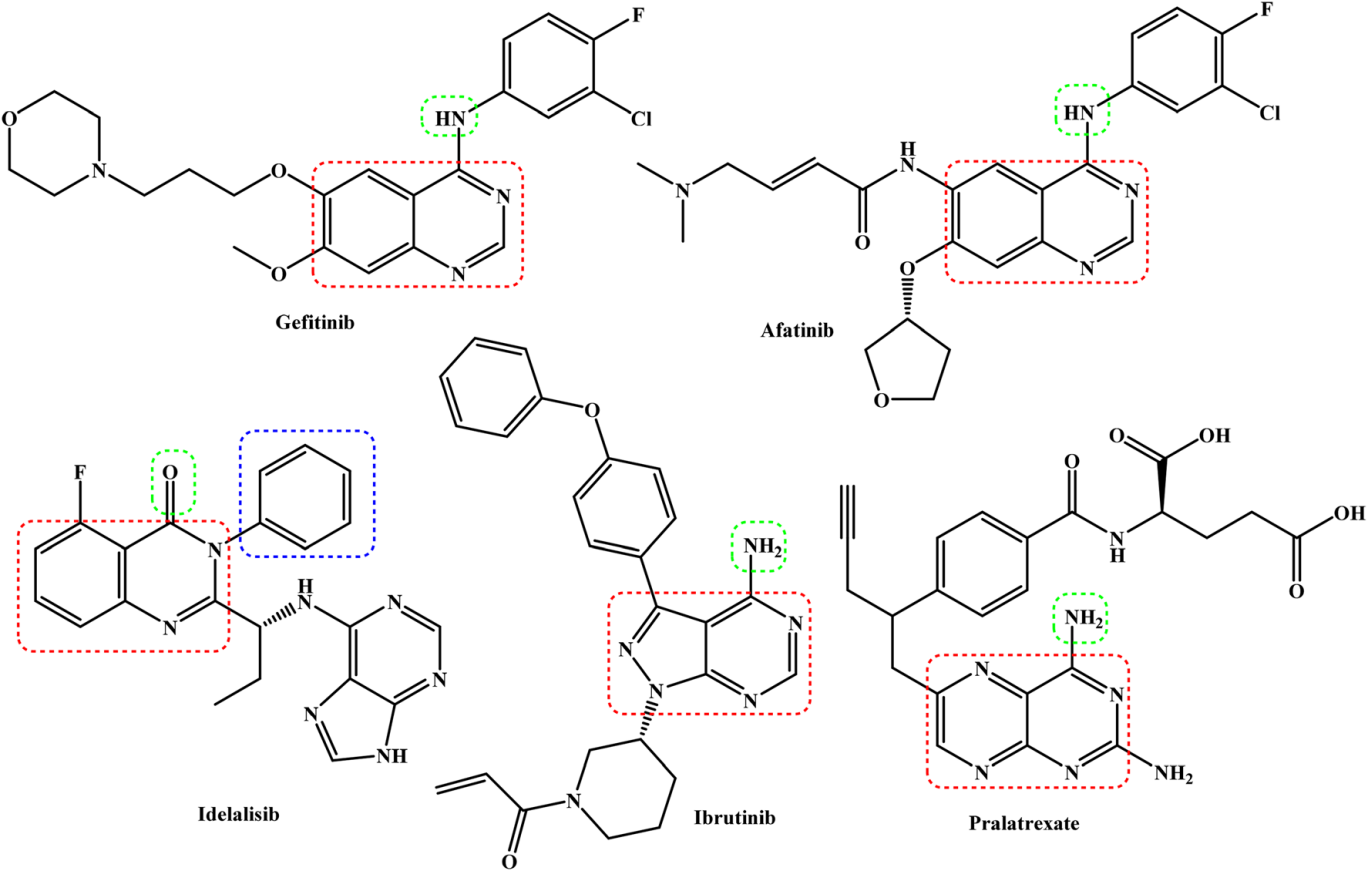
FDA-approved pyrimidine-fused bicyclic drugs for cancer therapy.

Pyrimidine-based heterocycles complement this strategy, being key motifs in clinically used anticancer drugs such as gefitinib, afatinib, idelalisib, ibrutinib, and pralatrexate (Fig. 2).^30^ These scaffolds modulate diverse molecular targets involved in cancer progression, including kinases and DNA synthesis enzymes, and apoptotic pathways. Pyrimidinones, in particular, have been validated as effective chemotherapeutic agents due to their ability to inhibit tumor cell proliferation, induce apoptosis, and overcome drug resistance.^31–33^ The incorporation of pyrimidine moieties into chromene-based derivatives thus represents a promising approach for the development of novel multitargeted anticancer agents, providing a clear rationale for the design and synthesis of the compounds investigated in this study. In view of the promising anticancer activities of both benzochromene and pyrimidine scaffolds, this study focuses on the design, synthesis, and biological evaluation of novel chromene–pyrimidine hybrid derivatives. By combining these two pharmacophores, we aim to develop multitargeted agents capable of inhibiting key cancer-related pathways, providing potential leads for further anticancer drug development.

Motivated by the reported anticancer potential of these scaffolds, and as part of our ongoing efforts to synthesize and biologically evaluate a variety of heterocyclic systems,^34–62^ we herein report the development of a facile synthetic approach to benzochromene-based derivatives, with the aim of assessing their cytotoxic properties and epidermal growth factor receptor (EGFR) kinase inhibitory activity.

### 1.1. Rational design of the work

**Fig. 3:**
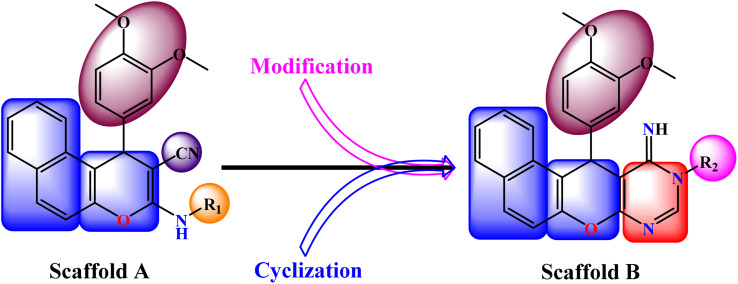
Design rationale of benzochromene-based scaffolds (A and B), highlighting key substitutions for optimized EGFR binding and selectivity.

Inspired by the established relevance of benzochromeno-pyrimidine scaffolds in kinase inhibition, a series of rationally designed derivatives was developed as prospective EGFR inhibitors. Structure-based design targeted the ATP-binding site, emphasizing hinge-region hydrogen bonding, π–π stacking, and hydrophobic interactions to enhance the affinity and selectivity. The design strategy encompassed the following aspects. (i) A dimethoxyphenyl moiety was incorporated to increase lipophilicity and promote favorable hydrophobic interactions within the non-polar regions of the kinase pocket. (ii) The benzochromeno-pyrimidine core was preserved to maintain molecular planarity, which is essential for effective π–π stacking and hydrogen-bonding interactions. (iii) The cyano (–CN) substituent was strategically incorporated as a strong electron-withdrawing group to increase the electron deficiency of the heterocyclic core, thereby strengthening hydrogen bonding and dipole–dipole interactions with active-site residues. (iv) Integration of the pyrimidine ring was specifically intended to provide multiple hydrogen-bond donor and acceptor sites, thereby strengthening interactions with critical catalytic amino acids and enhancing binding affinity. (v) Systematic modification of the R2 substituent was carried out to fine-tune the electronic and steric environment, allowing modulation of polarity, solubility, and binding geometry (Fig. 3).

## 2. Results and discussion

In this context, we focused on the construction of a new class of fused heterocycles based on the benzo[*f*]chromene framework, namely benzo[*f*]chromeno[2,3-*d*]pyrimidines and related derivatives, with the purpose of evaluating their antiproliferative activity. The benzo[*f*]chromene-2-carbonitrile scaffold 1, which serves as a pivotal synthetic precursor, was prepared *via* an efficient one-pot multicomponent protocol.^63^ This reaction involved the condensation of β-naphthol, malononitrile, and 3,4-dimethoxybenzaldehyde in absolute ethanol under basic catalysis using piperidine, affording the desired intermediate 1.

**Scheme 1:**
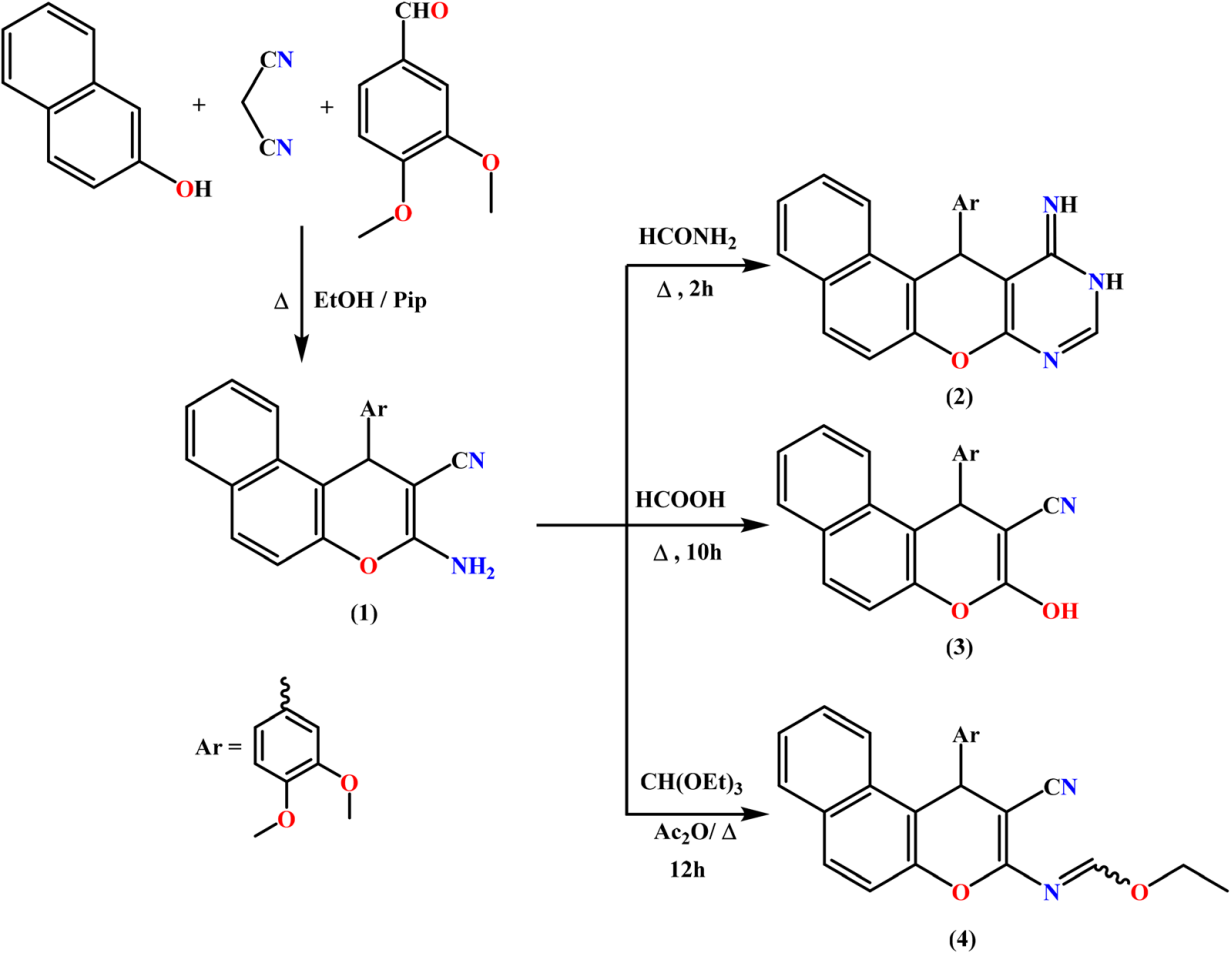
Reaction of enaminonitrile 1 with various one C-donors.

Treatment of 1 with formamide under heating promotes cyclocondensation involving the amino and cyano groups, resulting in the formation of a fused pyrimidinone-containing naphthopyran derivative 2 (Scheme 1). The suggested structure was corroborated by the IR spectrum, which showed no absorption band corresponding to the CN group. Furthermore, the ^1^H NMR and ^13^C NMR spectra were consistent with the proposed structure of 2.

Heating intermediate 1 in formic acid resulted in deamination of the NH_2_ group, affording the unexpected compound 1-(3,4-dimethoxyphenyl)-3-hydroxy-1*H*-benzo[*f*]chromene-2-carbonitrile (3) instead of the anticipated fused pyrimidinone derivative.

To increase the reactivity of the less active amino group in the enaminonitrile intermediate 1, it was refluxed for 12 hours with triethyl orthoformate in the presence of freshly distilled acetic anhydride. This reaction resulted in the formation of the corresponding ethyl formimidate derivative 4, as illustrated in Scheme 1.

**Scheme 2:**
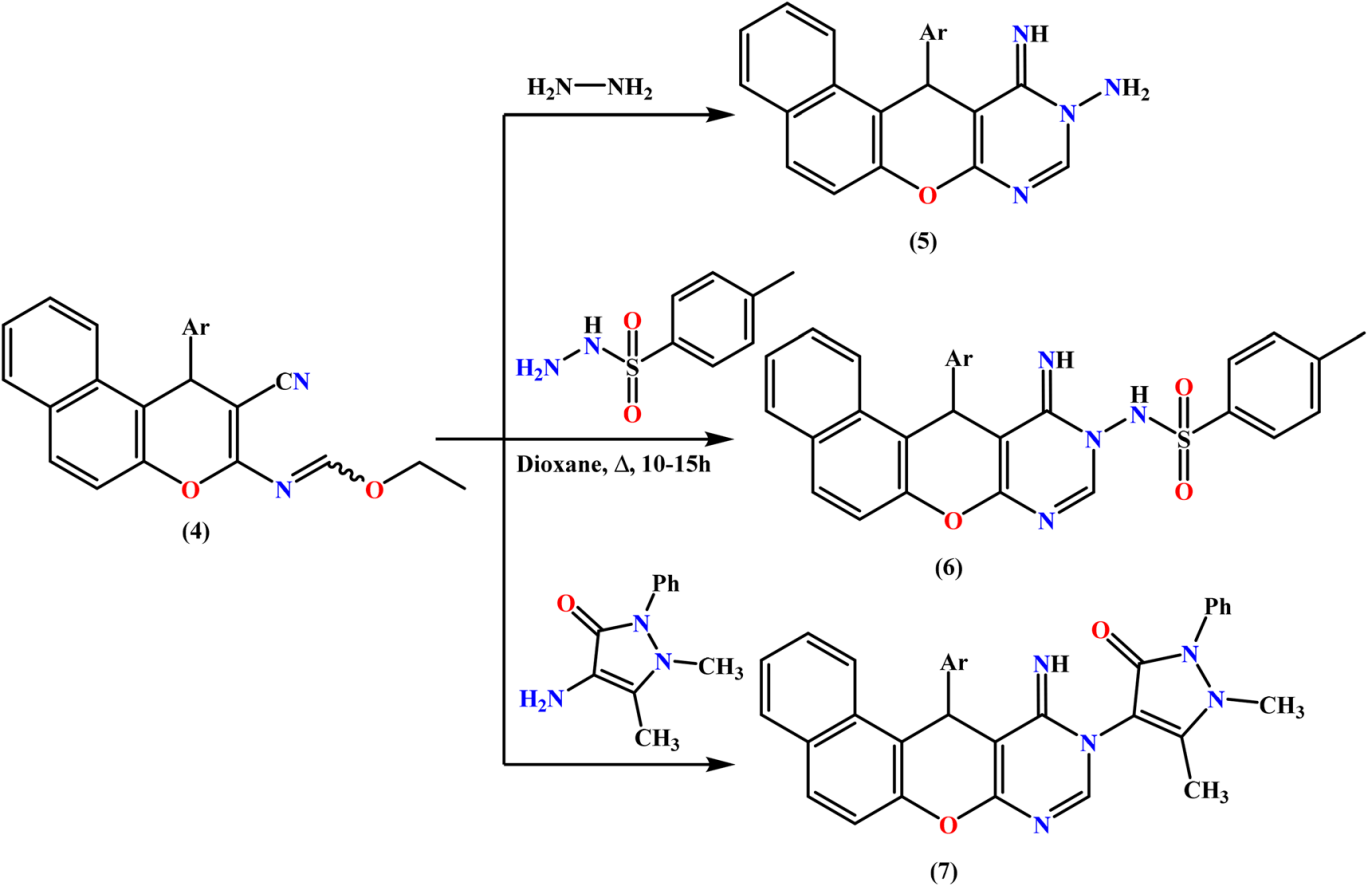
Treatment of ethyl formimidate 4 with some *N*-nucleophiles.

Ethyl formimidate 4 was reacted with different *N*-nucleophiles in an effort to synthesize novel benzochromenopyrimidines. Thus, treatment of compound 4 with hydrazine hydrate in absolute ethanol at room temperature for 6 hours produced the corresponding amino–imino derivative 5 in 78% yield (Scheme 2), and refluxing compound 4 with *p*-toluenesulfonohydrazide in 1,4-dioxane for 10 h produced benzochromenopyrimidine 6 as white crystals. The cyclized structure of 6 was confirmed by the expected elemental and spectroscopic analyses. The IR spectrum displayed an absorption band for the NH group at 3328 cm^−1^, while the absence of the nitrile absorption band was in accordance with the assigned structure 6.

Similarly, heating the tri-heterocyclic compound 4 with heterocyclic amines, such as 4-aminoantipyrine, in boiling 1,4-dioxane afforded the corresponding benzochromenopyrimidine derivative 7 in 68% yield, as illustrated in Scheme 2.

**Scheme 3:**
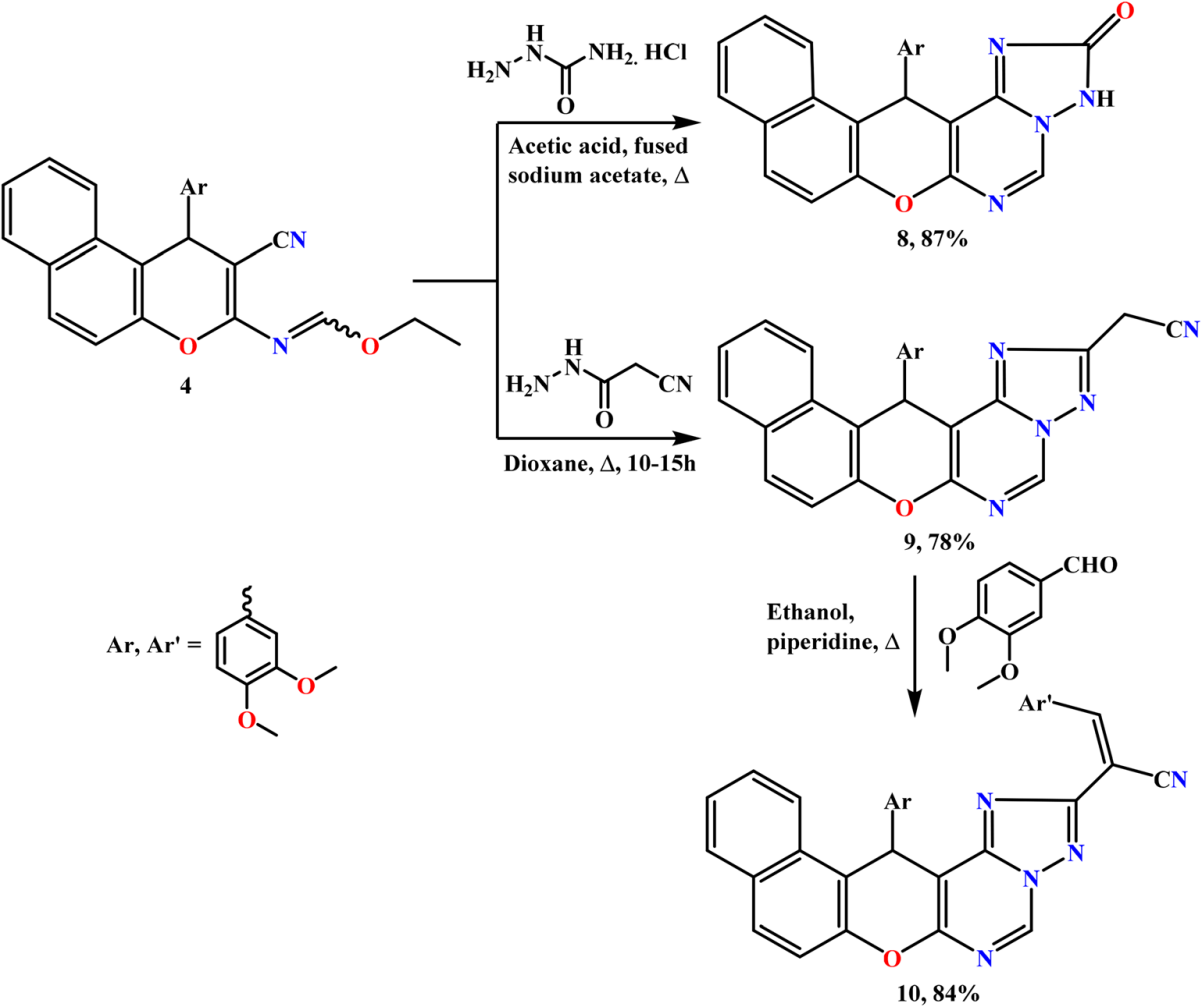
Reaction of ethyl formimidate 4 with some acid hydrazides.

In this study, polyfunctionalized binary and fused triazole scaffolds were synthesized to facilitate the incorporation of functional groups that are known to enhance biological activity. The design of these scaffolds was guided by structure–activity relationship (SAR) insights, and earlier studies have emphasized the insecticidal properties of 1,2,4-triazoles and related heterocyclic compounds.^34^ The reactivity of compound 4 toward selected acid hydrazides, namely semicarbazide hydrochloride and 2-cyanoacetohydrazide, was subsequently investigated (Scheme 3). In this regard, the triheterocyclic compound 4 acted as a key precursor for the construction of tetra- and penta-heterocyclic systems. Thus, heating the ethyl formimidate derivative 4 with semicarbazide hydrochloride in acetic acid containing fused sodium acetate yielded the corresponding benzochromenotriazolopyrimidine derivative 8 as the sole product in 87% yield. The structure of compound 8 was determined from its spectroscopic data; notably, the IR spectrum lacked the characteristic C≡N absorption band.

Similarly, reaction of 4 with 2-cyanoacetohydrazide in refluxing 1,4-dioxane yielded acetonitrile derivative 9 as yellow crystals in 78% yield. Its structure was proposed from analytical and spectral data: the IR spectrum exhibited a characteristic *ν*(C≡N) absorption at 2262 cm^−1^, and the ^1^H NMR spectrum displayed a singlet signal at *δ* 4.52 ppm, attributable to the –CH_2_CN protons, confirming the successful cyclization.

Importantly, the base-catalyzed condensation of active methylene candidates with aromatic aldehydes yielded the corresponding Knoevenagel products. In a similar manner, the reaction of compound 9 with 3,4-dimethoxybenzaldehyde in refluxing ethanol in the presence of a catalytic amount of piperidine led to the formation of the desired arylidene derivative 10 (84%), as illustrated in Scheme 3. The structure of compound 10 was verified through elemental analysis and spectroscopic data.

## 3. Antiproliferative activity

The synthesized compounds (2–10) were assessed for their antiproliferative activity on three human cancer cell lines, HCT-116, MCF-7, and HepG2, alongside the normal human lung fibroblast cell line WI-38. The cytotoxic potency was expressed as IC_50_ values (µM), while the selectivity index (SI) was calculated relative to the normal cell line. The results were compared with the reference anticancer drug doxorubicin.

Among the tested compounds, derivatives 6, 7, and 10 demonstrated the most potent cytotoxic effects. In particular, compound 7 emerged as the most active candidate, displaying IC_50_ values of 5.98 ± 0.4 µM, 6.52 ± 0.4 µM, and 8.51 ± 0.6 µM against HCT-116, MCF-7, and HepG2 cells, respectively. These values approach those of doxorubicin, suggesting that compound 7 possesses strong anticancer potential. Importantly, compound 7 showed relatively low toxicity toward WI-38 cells (IC_50_ = 47.42 ± 3.8 µM), resulting in the highest selectivity index (SI = 6.7) among the tested compounds. This indicates that compound 7 preferentially targets cancer cells over normal cells, a desirable property for anticancer drug candidates.

Similarly, compounds 6 and 10 also demonstrated notable cytotoxic activities. Compound 6 exhibited IC_50_ values of 12.33 ± 1.4 µM, 10.66 ± 0.7 µM, and 7.26 ± 0.5 µM against HCT-116, MCF-7, and HepG2 cells, respectively, with an SI value of 5.4. Compound 10 also showed strong inhibition of cancer cell growth, particularly against MCF-7 (IC_50_ = 9.25 ± 0.6 µM) and HCT-116 (IC_50_ = 11.72 ± 1.2 µM), while maintaining relatively low toxicity toward WI-38 cells (IC_50_ = 67.32 ± 4.4 µM), leading to an SI of 5.6. These findings suggest that compounds 6 and 10 possess promising anticancer selectivity and may serve as valuable lead structures for further optimization (Table 1).

**Table 1:** Antiproliferative activity (IC_50_ in µM)^a^ of the newly synthesized compounds evaluated against colon (HCT-116), breast (MCF-7), and liver (HepG2) cancer cell lines and normal fibroblasts (WI-38), along with their corresponding selectivity indices (SI)

Compounds	HCT-116	MCF-7	HepG2	WI-38	SI^b^
2	65.17 ± 3.6	47.12 ± 2.8	71.24 ± 4.2	52.46 ± 4.2	0.9
3	56.88 ± 3.4	88.14 ± 4.6	>100	36.16 ± 3.8	0.4
4	75.38 ± 4.7	62.64 ± 3.4	98.60 ± 4.8	44.54 ± 3.8	0.5
5	54.32 ± 3.2	71.44 ± 4.4	68.16 ± 3.6	54.84 ± 4.2	0.8
6	12.33 ± 1.4	10.66 ± 0.7	7.26 ± 0.5	55.23 ± 4.4	5.4
7	5.98 ± 0.4	6.52 ± 0.4	8.51 ± 0.6	47.42 ± 3.8	6.7
8	61.49 ± 3.3	55.21 ± 3.4	75.86 ± 4.2	28.61 ± 1.8	0.4
9	92.54 ± 4.9	>100	81.63 ± 4.4	35.87 ± 2.2	0.3
10	11.72 ± 1.2	9.25 ± 0.6	15.14 ± 1.4	67.32 ± 4.4	5.6
DOX	5.23 ± 0.3	4.17 ± 0.2	4.50 ± 0.2	6.71 ± 0.5	1.4

a IC_50_ values are presented as the mean ± standard deviation (SD) from three independent experiments.

b SI: selectivity index, calculated as IC_50_ for WI-38 divided by the mean IC_50_ for the cancer cell lines.

In contrast, compounds 2, 3, 4, 5, 8, and 9 displayed moderate to weak cytotoxic activities, with IC_50_ values generally exceeding 50 µM against most tested cancer cell lines. Compound 3 showed relatively weak activity, particularly against HepG2 cells, where the IC_50_ exceeded 100 µM, indicating limited efficacy. Similarly, compounds 8 and 9 exhibited poor anticancer activity and low selectivity indices (0.4 and 0.3, respectively), suggesting that these derivatives lack sufficient potency and selectivity for further consideration.

## 4. ADME profiles

A computational ADME assessment was conducted using the SwissADME platform to evaluate the physicochemical properties, drug-likeness, and predicted oral bioavailability of compounds 2–10. Drug-likeness was assessed according to Lipinski's Rule of Five and Veber's criteria, widely recognized guidelines for evaluating the potential of compounds as orally active drug candidates.

Most synthesized derivatives exhibited physicochemical properties compatible with orally active small molecules. Compounds 2–5 and 8–9 possess molecular weights ranging from 359.37 to 449.46 g mol^−1^, remaining within the recommended Lipinski threshold (<500 g mol^−1^). Compounds 6, 7, and 10 showed higher molecular weights (554.62–597.62 g mol^−1^), resulting in a single Lipinski violation. Nevertheless, their hydrogen-bonding capacities remained within acceptable limits (HBA = 5–9 and HBD = 0–2), indicating adequate polarity for intermolecular interactions without excessive hydrophilicity. The calculated lipophilicity values (log *P* = 3.09–5.27) indicate moderate hydrophobicity across the series, a property generally favorable for passive membrane diffusion. Most compounds fall within the recommended lipophilicity range (log *P* ≤ 5), with compound 10 slightly exceeding this value. All derivatives exhibited a predicted bioavailability score of 0.55, indicating a moderate probability of oral absorption.

According to Veber's criteria, TPSA values ranged from 71.71 to 123.91 Å^2^, well below the recommended threshold (≤140 Å^2^), supporting favorable intestinal permeability. The number of rotatable bonds (3–7) indicates moderate molecular flexibility, which may facilitate ligand–target interactions while preserving structural stability. Importantly, no Veber's rule violations were detected (Table 2, SI).

**Table 2:** Physicochemical and pharmacokinetic properties affecting the bioavailability of the evaluated compounds

Compound	MW	HBA	HBD	TPSA	Rotatable bonds	Bioavailability score	Log *P* ≤ 5	Lipinski violations	Veber violations
2	385.42	5	2	80.22	3	0.55	3.68	0	0
3	359.37	5	1	71.71	3	0.55	3.72	0	0
4	414.45	6	0	73.07	6	0.55	4.48	0	0
5	400.43	5	2	95.38	3	0.55	3.09	0	0
6	554.62	7	2	123.91	6	0.55	4.40	1	0
7	571.63	6	1	96.29	5	0.55	4.98	1	0
8	426.42	6	1	90.74	3	0.55	3.32	0	0
9	449.46	7	0	94.56	4	0.55	3.79	0	0
10	597.62	9	0	113.02	7	0.55	5.27	1	0

Among the evaluated derivatives, compound 4 displays one of the most balanced physicochemical profiles (MW = 414.45 g mol^−1^, log *P* = 4.48, TPSA = 73.07 Å^2^, and six rotatable bonds), reflecting an optimal balance between lipophilicity, polarity, and conformational flexibility that may favor membrane permeability and pharmacokinetic performance.

To further evaluate absorption and brain penetration properties, the BOILED-Egg model was employed. This predictive model correlates lipophilicity (*W* log *P*) and TPSA to estimate passive GI absorption and blood–brain barrier permeability. In the BOILED-Egg diagram, the white area indicates compounds with a high probability of human intestinal absorption (HIA), while the yellow region (yolk) represents molecules predicted to cross the central nervous system (CNS).

**Fig. 4:**
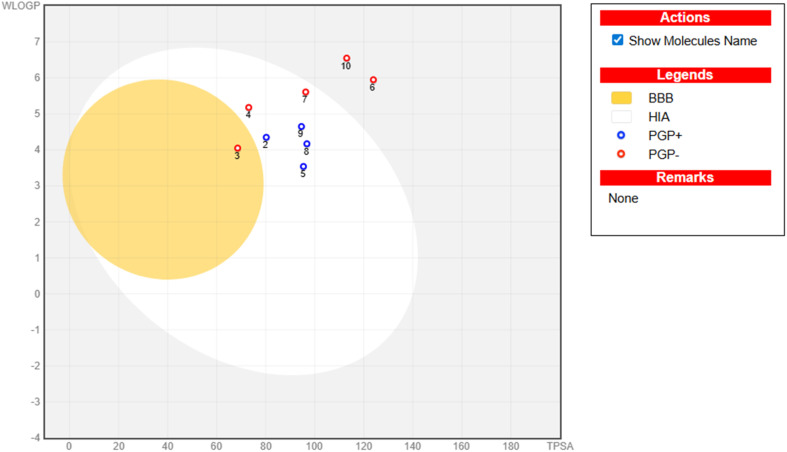
BOILED-Egg model illustrating the predicted gastrointestinal absorption and brain penetration potential of the synthesized candidates.

The BOILED-Egg model was additionally used to predict gastrointestinal absorption and blood–brain barrier (BBB) permeability based on lipophilicity and TPSA. As illustrated in Fig. 4, most compounds (2 and 4–10) fall within the white region of the diagram, indicating a high probability of human intestinal absorption. Interestingly, compound 3 appears in the yellow region (yolk), suggesting a potential ability to penetrate the blood–brain barrier. Such behavior may indicate possible central nervous system exposure, whereas the remaining derivatives are predicted to have limited BBB permeability, which may be advantageous for peripherally acting therapeutic agents by reducing the risk of neurological side effects.

The BOILED-Egg analysis also provides insight into interactions with *P*-glycoprotein, a key efflux transporter involved in drug absorption and distribution. Several derivatives are predicted to behave as non-substrates, indicating a reduced probability of efflux-mediated elimination and potentially improved intracellular retention following absorption.

Overall, the integrated ADME and BOILED-Egg analyses indicate that the synthesized compounds possess generally favorable pharmacokinetic characteristics, including acceptable drug-likeness and predicted oral absorption. Most derivatives exhibit limited blood–brain barrier (BBB) permeability, whereas compound 3 is predicted to penetrate the BBB. These findings suggest that careful optimization of molecular size, lipophilicity, and polarity within this scaffold may yield compounds with promising pharmacokinetic profiles suitable for further biological evaluation.

## 5. Molecular docking

**Fig. 5:**
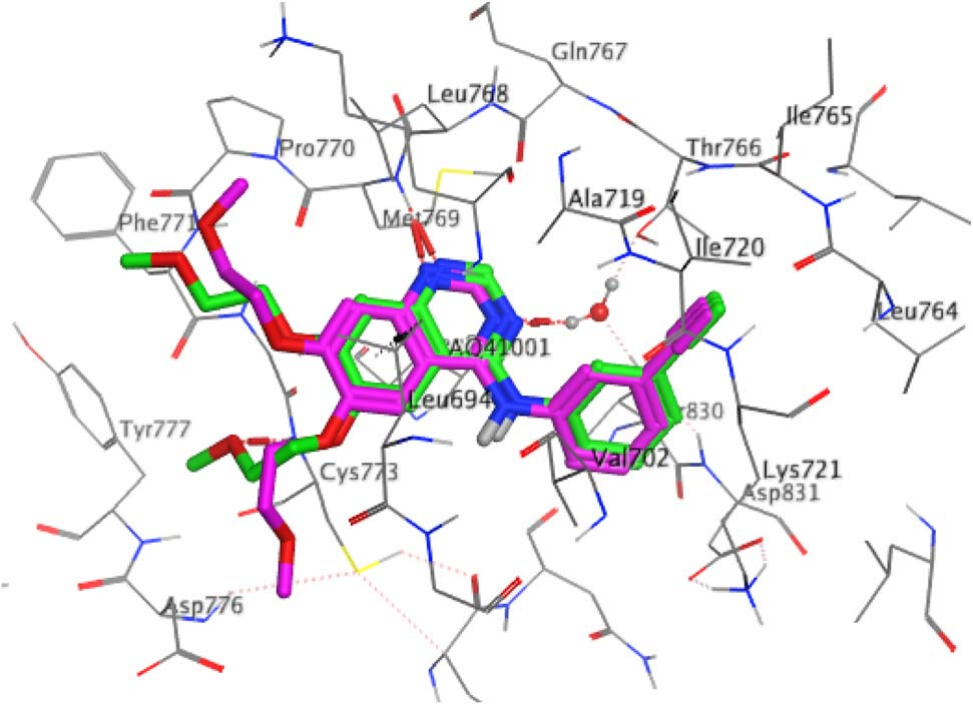
Superimposition of the docked AQ4 ligand (green) with the original ligand (pink) showing a RMSD value of 1.34 Å and a binding score of −9.12 kcal mol^−1^.

Molecular docking simulations were conducted to evaluate the binding affinity and interaction patterns of the prepared compounds toward the epidermal growth factor receptor (EGFR) tyrosine kinase using the crystal structure PDB ID: 4HJO. The docking protocol was validated by re-docking the co-crystallized ligand into the active site of the receptor. The obtained RMSD value of 1.34 Å confirmed that the docking procedure was able to reproduce the experimentally observed binding mode with acceptable accuracy, indicating the reliability of the applied docking parameters (Fig. 5).

The anticancer drug gefitinib, a clinically approved EGFR inhibitor, was used as a reference ligand to compare the binding affinity and interaction behavior of the benzochromene derivatives. Gefitinib achieved a docking score of −8.249 kcal mol^−1^ and established key hydrogen bonds with LEU 694 (A) and MET 769 (A), along with hydrophobic π–H interactions within the ATP-binding pocket. These residues are essential for stabilizing inhibitors within the EGFR kinase domain.

The docking scores of the investigated compounds ranged from −5.941 to −9.067 kcal mol^−1^, suggesting variable but generally favorable binding affinities toward the EGFR active site. Notably, most compounds established interactions with essential residues located within the ATP-binding region, including LYS 692, LEU 694, MET 769, CYS 773, and ASP 776, which are crucial for ligand recognition and stabilization (Table 3, SI).

**Table 3:** Docking scores and interaction patterns of the synthesized compounds with the EGFR tyrosine kinase

Ligands	*S* (kcal mol^−1^)	rmsd_refine (Å)	Binding site		Interaction	Distance (Å)	*E* (kcal mol^−1^)
			Ligand^a^	Receptor			
2	−6.333	1.248	N 31	LYS 692	H-Acceptor	2.92	−10.4
			6-Ring	LEU 694	Pi-H	4.32, 4.07	−1.0, −0.8
3	−5.941	1.092	O 1	LYS 692	H-Acceptor	3.08	−10.4
			N 5	LEU 694	H-Acceptor	3.50	−0.9
			O 1	LYS 692	Ionic	3.08	−3.9
			6-Ring	LEU 694	Pi-H	4.11	−0.6
4	−6.731	1.686	6-Ring	LEU 694	Pi-H	4.02, 3.69	−0.7, −1.0
			6-Ring	GLY 772	Pi-H	4.00	−0.7
5	−6.031	1.850	O 12	LYS 692	H-Acceptor	2.94	−0.8
			N 31	ASP 776	Ionic	3.85	−0.8
			6-Ring	LEU 694	Pi-H	4.45, 3.98	−0.6, −0.8
6	−7.937	0.933	O 49	MET 769	H-Acceptor	3.51	−0.6
			6-Ring	LYS 692	Pi-cation	3.67, 4.31	−1.1
			6-Ring	LEU 694	Pi-H	3.97	−0.9
7	−9.067	1.747	N 1	ARG 817	H-Donor	2.74	−3.4
			5-Ring	THR 830	Pi-H	4.36	−1.6
8	−6.519	1.403	6-Ring	LEU 694	Pi-H	4.04, 3.75	−1.0, −1.3
			6-Ring	GLY 772	Pi-H	3.89	−1.0
9	−6.712	1.094	N 30	MET 769	H-Acceptor	3.55	−1.6
			6-Ring	CYS 773	Pi-H	3.82, 3.46	−0.8
			5-Ring	CYS 773	Pi-H	4.32	−0.7
10	−8.175	1.610	N 28	MET 769	H-Acceptor	3.17	−3.7
			6-Ring	LYS 692	Pi-cation	3.98	−1.4
Gefitinib reference	−8.249	1.383	N 42	LEU 694	H-Donor	3.20	−5.9
			N 14	MET 769	H-Acceptor	3.04	−4.8
			6-Ring	LEU 694	Pi-H	3.63	−0.7
Co-crystallized ligand	−8.674	2.039	N 2 43	MET 769	H-Acceptor	3.02	−4.8
			6-Ring	LEU 694	Pi-H	3.63	−0.8

a Repeated entries of “6-ring” represent the different ligand rings or functional groups interacting at distinct positions within the EGFR binding site.

**Fig. 6:**
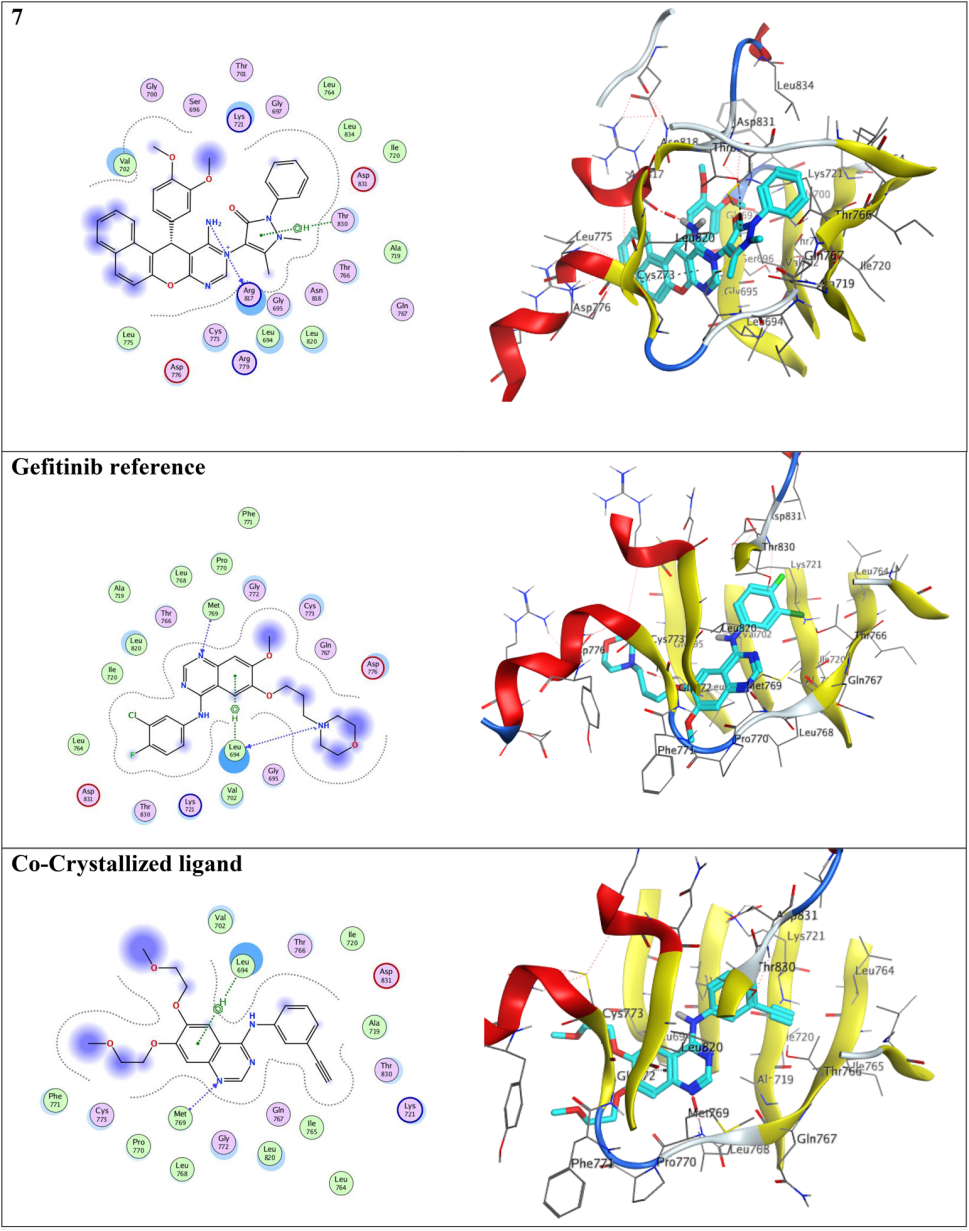
Schematic of the benzochromenopyrimidine derivative 7 illustrating its 2D and 3D interactions within the active site of the EGFR tyrosine kinase.

Among the evaluated compounds, compound 7 showed the most promising binding affinity, achieving a docking score of −9.067 kcal mol^−1^, surpassing that of the reference drug gefitinib. The ligand formed a strong hydrogen-bond donor interaction with ARG 817 at a distance of 2.74 Å, accompanied by a π–H interaction with THR 830. These interactions contribute significantly to stabilizing the ligand within the EGFR binding pocket and indicate a favorable binding orientation that may enhance inhibitory potential, as illustrated in Table 3 and Fig. 6. Compound 10 also demonstrated a promising binding profile, with a docking score of −8.175 kcal mol^−1^, comparable to that of gefitinib. The ligand established a hydrogen bond with MET 769 at 3.17 Å, together with a π–cation interaction with LYS 692 at 3.98 Å. Such interactions are frequently observed in potent EGFR inhibitors and suggest that compound 10 effectively occupies the ATP-binding site.

Similarly, compound 6 displayed a favorable docking score of −7.937 kcal mol^−1^ and a low RMSD value (0.933 Å), indicating a stable and reliable docking pose. The compound formed a hydrogen-bond interaction with MET 769 and multiple π–cation interactions with LYS 692, along with π–H interactions with LEU 694. These interactions emphasize the importance of aromatic systems in promoting hydrophobic and electrostatic contacts within the kinase active site.

Compounds 2, 3, and 5 exhibited moderate docking scores (−6.333, −5.941, and −6.031 kcal mol^−1^, respectively). Their binding modes were primarily stabilized through hydrogen-bond interactions with LYS 692 and π–H interactions with LEU 694. In addition, compound 3 formed an ionic interaction with LYS 692, while compound 5 showed an ionic interaction with ASP 776, suggesting that electrostatic contacts also contribute to ligand stabilization within the EGFR binding cavity.

Compounds 8 and 9 presented docking scores of −6.519 and −6.712 kcal mol^−1^, respectively. Their binding orientations were predominantly stabilized by hydrophobic π–H interactions with residues such as LEU 694, GLY 772, and CYS 773. Furthermore, compound 9 established a hydrogen-bond acceptor interaction with MET 769, which further enhanced the stabilization of the ligand within the active site. The interaction analysis also showed that several synthesized compounds mimic the key interactions observed for gefitinib, particularly those involving MET 769 and LEU 694, confirming that these residues are critical anchors within the EGFR ATP-binding pocket. The presence of aromatic rings and heteroatoms in the synthesized molecules appears to facilitate the formation of hydrogen bonds, π–H interactions, and π–cation contacts with these residues, thereby enhancing their binding affinity.

Importantly, while compound 7 exhibited a docking score numerically higher than that of gefitinib, the docking score alone is not sufficient to conclusively determine promising binding affinity. Therefore, additional parameters, such as RMSD values and detailed interaction profiles, were considered to support the reliability of the predicted binding modes. All reported RMSD values (<2 Å) indicate acceptable and stable docking conformations. Furthermore, several synthesized compounds mimic key interactions observed for gefitinib, particularly those involving MET 769 and LEU 694, which are critical anchors within the EGFR ATP-binding pocket. It should also be emphasized that molecular docking provides a static representation of ligand–protein interactions and does not fully account for solvent effects, protein flexibility, or binding kinetics. Consequently, further studies, such as molecular dynamics simulations and experimental kinetic validation, are required for a more comprehensive assessment of binding stability and inhibitory potential.

Overall, the docking results indicate that hydrogen bonding, hydrophobic π–interactions, and electrostatic contacts play fundamental roles in stabilizing ligand binding within the EGFR kinase domain. Among the tested compounds, 7, 10, and 6 demonstrated the most favorable binding profiles, with docking scores comparable to or better than that of the reference inhibitor gefitinib. These findings suggest that these compounds may represent promising scaffolds for further development as potential EGFR tyrosine kinase inhibitors.

## 6. Structure–activity relationship

The benzochromene derivatives 2–5 showed weak to moderate cytotoxic activity (IC_50_ > 50 µM), indicating that the basic benzochromene scaffold with simple substituents provides limited interactions within the EGFR binding pocket. Docking results suggest that these compounds mainly form weak hydrophobic π–H interactions with residues such as LEU 694.

Introducing nitrogen-rich heterocyclic systems significantly enhanced activity. Compounds 6 and 7 displayed strong antiproliferative effects, likely owing to the presence of additional heteroatoms that facilitate hydrogen bonding and electrostatic interactions with key residues such as LYS 692 and MET 769. Among them, compound 7 showed the highest activity and promising binding affinity, attributed to its extended heteroaromatic system that improves both hydrogen bonding and hydrophobic stabilization within the EGFR active site. ADME predictions indicate that compounds 6 and 7 maintain moderate lipophilicity and acceptable oral bioavailability (bioavailability score = 0.55), supporting their potential as orally active leads.

In contrast, compounds 8 and 9 exhibited weak activity, possibly due to less favorable orientations of their heterocyclic moieties within the binding pocket. Compound 10 demonstrated notable activity, which may be related to the presence of the cyano group and additional heterocyclic features that enhance interactions with residues such as MET 769 and LYS 692. Despite a slightly higher log *P* (5.27) and molecular weight (597.62 g mol^−1^), its predicted oral absorption remains moderate, and limited BBB penetration may reduce potential CNS-related side effects. Overall, the results indicate that the incorporation of nitrogen-rich fused heterocycles, electron-withdrawing groups, and hydrogen-bonding functionalities enhances anticancer activity. Compounds 6, 7, and 10 emerge as the most promising candidates, balancing potent EGFR interactions with favorable pharmacokinetic profiles.

## 7. Conclusion

Benzochromene-based derivatives (2–10) were successfully synthesized and structurally characterized, and their antiproliferative activities were evaluated against HCT-116, MCF-7, and HepG2 cancer cell lines, as well as normal WI-38 fibroblasts. Several compounds demonstrated promising anticancer activity, with good selectivity toward cancer cells. Among them, compounds 6, 7, and 10 exhibited the most potent cytotoxic effects. Notably, compound 7 showed the highest activity, with IC_50_ values comparable to the reference drug doxorubicin and relatively low toxicity toward normal WI-38 cells, resulting in a favorable selectivity index. Compounds 6 and 10 also displayed significant antiproliferative activity and acceptable selectivity profiles.

Molecular docking studies targeting the epidermal growth factor receptor (EGFR) tyrosine kinase suggested that the most active compounds (7, 10, and 6) may bind favorably within the EGFR ATP-binding pocket, showing docking scores comparable to that of gefitinib and forming key interactions with residues such as Lys692, Met769, and Leu694. However, these findings are based on computational predictions, and further experimental validation, such as enzymatic inhibition assays, is required to confirm their activity against EGFR. In addition, ADME and BOILED-Egg analyses indicated that these compounds possess generally favorable pharmacokinetic properties, including acceptable oral absorption, moderate lipophilicity, and limited blood–brain barrier penetration (with the exception of compound 3), supporting their potential for oral bioavailability.

Overall, the present study highlights benzochromene derivatives as promising scaffolds for anticancer drug development. Among the tested compounds, compound 7 emerges as a potential lead candidate for further optimization and biological investigation, particularly with respect to its mechanism of action and target validation.

## 8. Materials and methods

### 8.1. Chemistry

All melting points were determined using a Griffin and George melting-point apparatus (Griffin & Georgy Ltd, Wembley, Middlesex, UK). IR spectra were recorded with a Pye Unicam SP1200 spectrophotometer (Pye Unicam Ltd, Cambridge, UK) using the KBr pellet method. ^1^H NMR spectra were acquired with a 500 MHz JEOL NMR spectrometer with tetramethylsilane (TMS) as the internal standard (chemical shifts reported in *δ*), and ^13^C NMR spectra were recorded at 120 MHz. Elemental analyses were performed at the Microanalytical Unit, Faculty of Science, Ain Shams University, using a PerkinElmer 2400 CHN analyzer (Waltham, MA), yielding satisfactory results within ±0.4% for all compounds.

#### 8.1.1. 3-Amino-1-(3,4-dimethoxyphenyl)-1*H*-benzo[*f*]chromene-2-carbonitrile 1

A mixture of β-naphthol (1.44 g, 10 mmol), malononitrile (0.66 g, 10 mmol), and 3,4-dimethoxybenzaldehyde (1.66 mL, 10 mmol) in absolute ethanol (30 mL) with a catalytic amount of piperidine (0.5 mL) was refluxed for 10 hours. The resulting precipitate formed during heating was collected by filtration, dried, and recrystallized from ethanol to yield compound 1 as white crystals; yield 93%; mp 210 °C–212 °C (lit. 218 °C–220 °C);^62^ the slight deviation from the reported value is attributed to differences in instrumentation and heating conditions. IR (KBr, *ν*, cm^−1^): 3433, 3343 (NH_2_), 3071 (aromatic C–H), 2963, 2927 (aliphatic C–H), 2187 (C≡N), 1648 (C=N). Anal. calcd. for C_22_H_18_N_2_O_3_ (358.13): C, 73.73; H, 5.06; N, 7.82. Found: C, 73.85; H, 5.22; N, 7.68.

#### 8.1.2. 12-(3,4-Dimethoxyphenyl)-10,12-dihydro-11*H*-benzo[5,6]chromeno[2,3-*d*]pyrimidin-11-imine 2

Enaminonitrile 1 (3.58 g, 10 mmol) was dissolved in formamide (20 mL) and heated under reflux for 2 hours. After cooling, the mixture was poured onto crushed ice, and the resulting solid was filtered, washed with water, dried, and recrystallized from benzene to yield compound 2 as white crystals; yield 78%; mp 248 °C–250 °C; IR (KBr, *ν*, cm^−1^): 3337, 3171 (NH_2_), 2933, 2837 (aliphatic C–H), 1658 (C=N). ^1^H-NMR (500 MHz, DMSO-*d*_6_) *δ*_ppm_: 3.58 (s, 3H, OCH_3_), 3.65 (s, 3H, OCH_3_), 5.94 (s, 1H, C_4_H-pyran), 6.75 (d, 1H, Ar–H, *J* = 8.5 Hz), 6.86, 6.87 (dd, 1H, Ar–H, *J* = 1.5, 1.5 Hz), 7.18 (brs, 2H, NH_2_, exchangeable with D_2_O), 7.24 (d, 1H, Ar–H, *J* = 1.5 Hz), 7.34 (s, 1H, Ar–H), 7.43 (t, 1H, Ar–H, *J* = 7.5 Hz), 7.50 (d, 1H, Ar–H, *J* = 9 Hz), 7.57 (t, 1H, Ar–H, *J* = 7.5 Hz), 7.91 (d, 2H, Ar–H, *J* = 9 Hz), 8.10 (s, 1H, Ar–H). ^13^C-NMR (125 MHz, DMSO-*d*_6_) *δ*_ppm_: 33.8, 55.3, 55.5, 97.2, 111.9, 117.6, 119.9, 123.2, 124.8, 127.0, 128.6, 129.2, 147.4, 147.7, 148.2, 156.2, 161.9, 162.4. Anal. calcd. for C_23_H_19_N_3_O_3_ (385.14): C, 71.68; H, 4.97; N, 10.90. Found: C, 71.73; H, 4.81; N, 10.79.

#### 8.1.3. 1-(3,4-Dimethoxyphenyl)-3-hydroxy-1*H*-benzo[*f*]chromene-2-carbonitrile 3

A mixture of enaminonitrile 1 (3.58 g, 10 mmol) and formic acid (20 mL) was heated under reflux for 10 h. The precipitated solid formed during reflux was collected by filtration, dried, and recrystallized from ethanol to afford compound 3 as white crystals; yield 80%; mp 238 °C–240 °C; IR (KBr, *ν*, cm^−1^): 3078 (aromatic C–H), 2957, 2892 (aliphatic C–H), 2254 (C≡N), 1678 (C=O_lactone_, keto form). ^1^H-NMR (500 MHz, DMSO-*d*_6_) *δ*_ppm_: 3.66 (s, 3H, OCH_3_), 3.72 (s, 3H, OCH_3_), 5.53 (s, 1H, C4-Hpyran + 1H, OH), 6.57, 6.99 (dd, 1H, Ar–H, *J* = 1.5 Hz), 6.85 (d, 1H, Ar–H, *J* = 8 Hz), 7.08 (d, 1H, Ar–H, *J* = 2.5 Hz), 7.49–7.52 (m, 1H, Ar–H), 7.57 (t, 1H, Ar–H, *J* = 7 Hz), 7.98 (d, 1H, Ar–H, *J* = 9 Hz), 8.05 (d, 1H, Ar–H, *J* = 9 Hz). ^13^C-NMR (125 MHz, DMSO-*d*_6_) *δ*_ppm_: 40.8, 55.3, 55.4, 112.1, 117.0, 117.2, 119.3, 123.4, 125.7, 127.7, 128.6, 128.9, 129.8, 130.4, 130.8, 148.2, 148.7, 162.0. Anal. calcd. for C_22_H_17_NO_4_ (359.12): C, 73.53; H, 4.77; N, 3.90. Found: C, 73.44; H, 4.69; N, 3.98.

#### 8.1.4. Ethyl (*E*)-*N*-(2-cyano-1-(3,4-dimethoxyphenyl)-1*H*-benzo[*f*]chromen-3-yl)formimidate 4

Enaminonitrile 1 (3.58 g, 10 mmol) was mixed with triethyl orthoformate (30 mL) and acetic anhydride (15 mL) and refluxed for 10 hours. The resulting precipitate was collected by filtration, washed with light petroleum ether (40 °C–60 °C), and recrystallized from ethanol to afford compound 4 as pale-yellow crystals; yield 77%; mp 184 °C–186 °C; IR (KBr, *ν*, cm^−1^): 3058 (aromatic C–H), 2979, 2834 (aliphatic C–H), 2207 (C≡N), 1652 (C=N). ^1^H-NMR (500 MHz, DMSO-*d*_6_) *δ*_ppm_: 1.30 (t, 3H, C̲H̲_3_CH_2_, *J* = 7.5 Hz), 3.65 (s, 3H, OCH_3_), 3.67 (s, 3H, OCH_3_), 4.31 (q, 2H, CHE_3_C̲H̲_2_, *J* = 7 Hz), 5.50 (s, 1H, C4-Hpyran), 6.63, 6.65 (dd, 1H, Ar–H, *J* = 2, 1.5 Hz), 6.82 (d, 1H, Ar–H, *J* = 8 Hz), 6.96 (s, 1H, Ar–H), 7.41–7.46 (m, 3H, Ar–H), 7.86 (d, 1H, Ar–H, *J* = 8 Hz), 7.90–7.92 (m, 1H, Ar–H), 7.96 (d, 1H, Ar–H, *J* = 8.5 Hz), 8.70 (s, 1H, Ar–H). ^13^C-NMR (125 MHz, DMSO-*d*_6_) *δ*_ppm_: 13.9, 55.4, 55.5, 63.9, 81.3, 111.4, 112.0, 117.1, 119.7, 123.9, 125.1, 127.1, 128.5, 129.8, 131.1, 146.9, 147.8, 148.7, 156.6, 161.8. Anal. calcd. for C_25_H_22_N_2_O_4_ (414.16): C, 72.45; H, 5.35; N, 6.76. Found: C, 72.51; H, 5.29; N, 6.63.

#### 8.1.5. 12-(3,4-Dimethoxyphenyl)-11-imino-11*H*-benzo[5,6]chromeno[2,3-*d*]pyrimidin-10(12*H*)-amine 5

Formimidate derivative 4 (4.14 g, 10 mmol) was stirred with hydrazine hydrate (98%) (0.5 mL, 10 mmol) in absolute ethanol (20 mL) at room temperature for 6 hours. The resulting precipitate was collected and recrystallized from a light petroleum ether (60 °C–80 °C)/benzene mixture to yield the product 5 as white crystals; yield 82%; mp 116 °C–118 °C; IR (KBr, *ν*, cm^−1^): 3445, 3350, 3304 (NH, NH_2_), 3034 (aromatic C–H), 2935, 2836 (aliphatic C–H), 1652 (C=N). ^1^H-NMR (500 MHz, DMSO-*d*_6_) *δ*_ppm_: 3.59 (s, 3H, OCH_3_), 3.67 (s, 3H, OCH_3_), 5.72 (brs, 2H, NH_2_, exchangeable with D_2_O), 5.58 (s, 1H, C4-Hpyran), 6.72 (d, 1H, Ar–H, *J* = 8 Hz), 6.84 (s, 1H, Ar–H), 7.29 (s, 1H, Ar–H), 7.34 (s, 1H, Ar–H), 7.41 (t, 1H, Ar–H, *J* = 7 Hz), 7.45–7.47 (m, 1H, Ar–H), 7.52 (t, 1H, Ar–H, *J* = 7 Hz), 7.88 (d, 2H, *J* = 8.5 Hz), 8.09 (s, 1H, N=CH), 8.25 (brs, 1H, NH, exchangeable with D_2_O). ^13^C-NMR (120 MHz, DMSO-*d*_6_) *δ*_ppm_: 35.4, 55.3, 55.5, 99.7, 117.4, 120.4, 124.8, 126.9, 128.5, 129.1, 130.5, 130.9, 147.3, 147.4, 148.1, 150.1, 155.1. Anal. calcd. for C_23_H_20_N_4_O_3_ (400.15): C, 68.99; H, 5.03; N, 13.99. Found: C, 68.84; H, 5.17; N, 13.78.

#### 8.1.6. *N*-(12-(3,4-Dimethoxyphenyl)-11-imino-11*H*-benzo[5,6]chromeno[2,3-*d*]pyrimidin-10(12*H*)-yl)-4-methylbenzenesulfonamide 6

Formimidate derivative 4 (4.14 g, 10 mmol) was refluxed with *p*-toluenesulfonohydrazide (10 mmol) in absolute ethanol (30 mL) for 8 hours. The precipitate formed during heating was collected by filtration, dried, and recrystallized from ethanol/1,4-dioxane mixture to afford 6 as white crystals; yield 77%; mp 186 °C–188 °C; IR (KBr, *ν*, cm^−1^): 3328 (NH), 3081 (aromatic C–H), 2931, 2832 (aliphatic C–H), 1643 (C=N). ^1^H-NMR (500 MHz, DMSO-*d*_6_) *δ*_ppm_: 2.18 (s, 3H, CH_3_), 3.63 (s, 3H, OCH_3_), 3.64 (s, 3H, OCH_3_), 5.99 (s, 1H, C4-Hpyran), 6.72–6.77 (m, 2H, Ar–H), 6.85 (s, 1H, Ar–H), 7.21 (s, 1H, Ar–H), 7.25 (d, 2H, Ar–H), 7.46 (t, 1H, Ar–H, *J* = 7 Hz), 7.54–7.91 (m, 2H, Ar–H), 7.94 (t, 2H, Ar–H, *J* = 7 Hz), 8.10 (d, 1H, ArH), 8.21 (brs, 1H, NH, exchangeable with D_2_O), 8.90 (s, 1H, N=CH), 8.99 (brs, 1H, NH, exchangeable with D_2_O). ^13^C-NMR (120 MHz, DMSO-*d*_6_) *δ*_ppm_: 20.6, 33.8, 55.3, 55.4, 98.5, 111.5, 111.8, 116.5, 117.3, 119.8, 123.1, 125.4, 125.8, 127.4, 128.7, 129.6, 129.9, 134.3, 140.9, 146.7, 152.4, 158.4. Anal. calcd. for C_30_H_26_N_4_O_5_S (554.16): C, 64.97; H, 4.73; N, 10.10; S, 5.78. Found: C, 64.86; H, 4.61; N, 10.23; S, 5.59.

#### 8.1.7. 4-(12-(3,4-Dimethoxyphenyl)-11-imino-11*H*-benzo[5,6]chromeno[2,3-*d*]pyrimidin-10(12*H*)-yl)-1,5-dimethyl-2-phenyl-1,2-dihydro-3*H*-pyrazol-3-one 7

A mixture of formimidate derivative 4 (4.14 g, 10 mmol) and 4-aminoantipyrine (10 mmol) in 30 mL of butanol was refluxed for 10 hours. After concentrating the reaction mixture, the resulting solid was filtered, dried, and recrystallized from 1,4-dioxane to afford 7 as yellow crystals; yield 68%; mp 180 °C–182 °C; IR (KBr, *ν*, cm^−1^): 3223 (NH), 3060 (aromatic C–H), 2933, 2834 (aliphatic C–H), 1651 (C=N). ^1^H-NMR (500 MHz, DMSO-*d*_6_) *δ*_ppm_: 1.86 (s, 3H, CH_3_), 3.08 (s, 3H, NCH_3_), 3.58 (s, 3H, OCH_3_), 3.66 (s, 3H, OCH_3_), 6.18 (s, 1H, C4-Hpyran), 6.73 (s, 2H, Ar–H), 7.34 (t, 1H, Ar–H, *J* = 7 Hz), 7.42–7.45 (m, 4H, Ar–H), 7.51–7.55 (m, 4H, Ar–H), 7.93 (t, 2H, Ar–H, *J* = 8 Hz), 8.14 (d, 1H, ArH, *J* = 6.5 Hz), 8.68 (brs, 1H, NH, exchangeable with D_2_O). ^13^C-NMR (120 MHz, DMSO-*d*_6_) *δ*_ppm_: 10.2, 33.4, 55.3, 55.5, 98.8, 112.1, 117.6, 119.7, 123.7, 129.1, 129.2, 130.6, 130.8, 147.3, 147.7, 148.1, 153.3, 155.8, 160.6, 161.7, 162.5. Anal. calcd. for C_34_H_29_N_5_O_4_ (571.22): C, 71.44; H, 5.11; N, 12.25. Found: C, 71.66; H, 5.07; N, 12.11.

#### 8.1.8. 14-(3,4-Dimethoxyphenyl)-14*H*-benzo[5,6]chromeno[3,2-*e*][1,2,4]triazolo[1,5-*c*]pyrimidin-2(3*H*)-one 8

Formimidate derivative 4 (4.14 g, 10 mmol) and semicarbazide hydrochloride (10 mmol) were combined in 20 mL of acetic acid containing a catalytic amount of fused sodium acetate (0.5 g) and refluxed for 10 hours. Upon cooling, the mixture was poured into 50 mL of water, and the precipitated solid was filtered, dried, and recrystallized from an ethanol/1,4-dioxane mixture to yield 8 as white crystals; yield 87%; mp > 300 °C; IR (KBr, *ν*, cm^−1^): 3423 (NH), 3071 (aromatic C–H), 2929, 2833 (aliphatic C–H), 1660 (C=O), 1636 (C=N). ^1^H-NMR (500 MHz, DMSO-*d*_6_) *δ*_ppm_: 3.57 (s, 3H, OCH_3_), 3.66 (s, 3H, OCH_3_), 6.07 (s, 1H, C4-Hpyran), 6.71 (d, 2H, Ar–H, *J* = 8 Hz), 7.19 (s, 1H, Ar–H), 7.42 (t, 1H, Ar–H, *J* = 7 Hz), 7.48–7.51 (m, 1H, Ar–H), 7.55 (d, 1H, Ar–H, *J* = 9 Hz), 7.92 (d, 1H, ArH, *J* = 8 Hz), 7.97 (d, 1H, ArH, *J* = 9 Hz), 8.01 (d, 1H, ArH, *J* = 8 Hz), 9.25 (s, 1H, ArH), 12.64 (brs, 1H, NH, exchangeable with D_2_O). ^13^C-NMR (120 MHz, DMSO-*d*_6_) *δ*_ppm_: 36.3, 55.3, 55.5, 100.8, 111.7, 112.2, 117.5, 120.0, 125.0, 127.3, 128.6, 129.8, 147.7, 147.9, 148.4, 153.1, 139.9. Anal. calcd. for C_24_H_18_N_4_O_4_ (426.13): C, 67.60; H, 4.25; N, 13.14. Found: C, 67.88; H, 4.11; N, 13.29.

#### 8.1.9. 2-(14-(3,4-Dimethoxyphenyl)-14*H*-benzo[5,6]chromeno[3,2-*e*][1,2,4]triazolo[1,5-*c*]pyrimidin-2-yl)acetonitrile 9

A mixture of formimidate derivative 4 (4.14 g, 10 mmol) and 2-cyanoacetohydrazide (10 mmol) in 30 mL of dry 1,4-dioxane was refluxed for 10 hours. After cooling, the formed solid was filtered, dried, and recrystallized from 1,4-dioxane to give compound 9 as pale-yellow crystals; yield 78%; mp 198 °C–200 °C; IR (KBr, *ν*, cm^−1^): 3057 (aromatic C–H), 2953, 2842 (aliphatic C–H), 2262 (C≡N), 1635 (C=N). ^1^H-NMR (500 MHz, DMSO-*d*_6_) *δ*_ppm_: 3.54 (s, 3H, OCH_3_), 3.55 (s, 3H, OCH_3_), 4.52 (s, 2H, C̲H̲_2_CN), 6.20 (s, 1H, C4-Hpyran), 6.48, 6.50 (dd, 1H, Ar–H, *J* = 2 Hz), 6.66 (d, 1H, Ar–H, *J* = 8 Hz), 7.40–7.48 (m, 3H, Ar–H), 7.57 (d, 1H, Ar–H, *J* = 9 Hz), 7.92 (d, 1H, ArH, *J* = 8 Hz), 7.98 (d, 1H, ArH, *J* = 9 Hz), 8.02 (d, 1H, ArH, *J* = 8 Hz), 9.60 (s, 1H, ArH). ^13^C-NMR (125 MHz, DMSO-*d*_6_) *δ*_ppm_: 18.0, 36.4, 55.2, 55.4, 102.6, 111.7, 112.4, 114.7, 116.6, 117.4, 119.6, 123.7, 125.1, 127.3, 128.5, 129.9, 131.1, 135.3, 140.0, 147.5, 147.9, 148.3, 160.7. Anal. calcd. for C_26_H_19_N_5_O_3_ (449.15): C, 69.48; H, 4.26; N, 15.58. Found: C, 69.55; H, 4.37; N, 15.41.

#### 8.1.10. (*E*)-3-(3,4-Dimethoxyphenyl)-2-(14-(3,4-dimethoxyphenyl)-14*H*-benzo[5,6]chromeno[3,2-*e*][1,2,4]triazolo[1,5-*c*]pyrimidin-2-yl)acrylonitrile 10

Compound 9 (4.49 g, 10 mmol) and 3,4-dimethoxybenzaldehyde (1.66 mL, 10 mmol) were dissolved in 30 mL of absolute ethanol with a catalytic amount of piperidine and heated under reflux for 3 hours. The precipitated solid formed during reflux was filtered, dried, and recrystallized from 1,4-dioxane to afford 10 as yellow crystals; yield 84%; mp 280 °C–282 °C; IR (KBr, *ν*, cm^−1^): 3073, 3032 (CH aromatic), 2996 (CH aliphatic), 2220 (C≡N), 1635 (C=N). ^1^H-NMR (500 MHz, DMSO-*d*_6_) *δ*_ppm_: 3.35 (s, 3H, OCH_3_), 3.53 (s, 3H, OCH_3_), 3.81 (s, 3H, OCH_3_), 3.82 (s, 3H, OCH_3_), 6.20 (s, 1H, C4-Hpyran), 6.41, 6.42 (dd, 1H, Ar–H, *J* = 1.5, 1.5 Hz), 6.65 (d, 1H, Ar–H, *J* = 9 Hz), 7.06 (d, 1H, Ar–H, *J* = 9 Hz), 7.38–7.41 (m, 1H, Ar–H), 7.46 (t, 1H, ArH, *J* = 7 Hz), 7.56 (d, 1H, ArH, *J* = 10 Hz), 7.64 (d, 1H, ArH, *J* = 2.5 Hz), 7.73 (d, 1H, ArH, *J* = 2 Hz), 7.90–7.93 (m, 2H, ArH), 7.98 (d, 1H, ArH, *J* = 9.5 Hz), 8.47 (s, 1H, =NCH), 9.53 (s, 1H, =CH). ^13^C-NMR (120 MHz, DMSO-*d*_6_) *δ*_ppm_: 36.5, 55.2, 55.3, 55.4, 55.7, 97.1, 102.3, 111.8, 117.4, 125.1, 125.5, 130.4, 131.1, 135.2, 140.1, 148.1, 148.2, 148.7, 162.6. Anal. calcd. for C_35_H_27_N_5_O_5_ (597.20): C, 70.34; H, 4.55; N, 11.72. Found: C, 70.47; H, 4.33; N, 11.68.

### 8.2. *In Vitro* cytotoxic activity

Cytotoxic effects of the synthesized compounds were evaluated using three human cancer cell lines, breast adenocarcinoma (MCF-7), colon carcinoma (HCT-116), and hepatocellular carcinoma (HepG2), along with normal human fibroblasts (WI-38). All cell lines were obtained from the American Type Culture Collection (ATCC, Manassas, VA, USA) and cultured in RPMI-1640 medium supplemented with 10% heat-inactivated fetal bovine serum, 100 U mL^−1^ penicillin, and 100 µg mL^−1^ streptomycin, maintained at 37 °C in a humidified atmosphere containing 5% CO_2_.

Exponentially growing cells were detached using trypsin, counted, and seeded into 96-well plates at a density of approximately 2000 cells per well. Following a 24 hours incubation to ensure proper attachment, cells were treated with the synthesized compounds at concentrations ranging from 0.5 to 100 µM for 48 hours, while untreated cells served as controls.

Cell viability was determined using the MTT assay with full procedural details to ensure reproducibility. After treatment, the medium was removed, and 200 µL of MTT solution (5 mg mL^−1^) was added to each well, followed by a 4 hours incubation at 37 °C to allow formazan crystal formation. The MTT solution was then discarded, and the crystals were solubilized in DMSO for 30 minutes with continuous shaking at room temperature, protected from light, using a MaxQ 2000 plate shaker (Thermo Fisher Scientific, MA, USA). Absorbance was recorded at 570 nm using an ELISA plate reader. Cell viability was expressed as a percentage relative to the control group, and IC_50_ values (the concentration required to inhibit 50% of cell proliferation) were calculated.

### 8.3. *In silico* studies

#### 8.3.1. ADME predictions

The pharmacokinetic characteristics of the synthesized benzochromene derivatives, including absorption, distribution, metabolism, and excretion (ADME), were evaluated using the SwissADME online platform (https://www.swissadme.ch). This tool allows for the estimation of essential physicochemical properties and offers computational predictions regarding pharmacokinetics, drug-likeness, and suitability in medicinal chemistry. The chemical structures of the prepared compounds were first converted into SMILES (Simplified Molecular Input Line Entry System) format and then submitted to the server for *in silico* analysis.

#### 8.3.2. Molecular docking

All the synthesized benzochromene-based derivatives were subjected to molecular docking in order to explore their binding modes towards the EGFR receptor (PDB ID: 4HJO) retrieved from the Protein Data Bank (https://www.rcsb.org). The ChemOffice Ultra 2004 software was utilized to construct the chemical structures of the synthesized compounds. Subsequently, the three-dimensional structures of the synthesized derivatives, the reference drug, and the co-crystallized ligand were subjected to geometry optimization and energy minimization using the MMFF94X force field, with a convergence threshold of 0.05 kcal mol^−1^, as performed in the Avogadro 1.2.0. software.^64^ The prepared EGFR protein structure was then used as the docking target for the benzochromene derivatives. To validate the reliability of the docking protocol, the native co-crystallized ligand was re-docked into the active site using the AutoDock Vina software package. For each compound, the five top-ranked docking poses with the lowest binding energies were selected for further evaluation. The binding modes and ligand–receptor interactions within the EGFR active site were subsequently analyzed and visualized using the PyMOL software (https://www.pymol.org).^65^

## Author contributions

Data curation: MHH, FSMA and YMA. Formal analysis: MHH, YMA and AYAZ. Investigation: MHH, FSMA, AYAZ and SA. Writing-original draft: MHH and FSMA. Software: MHH, AYAZ and SA. Methodology: MHH, FSMA and YMA. Validation: MHH, AYAZ and SA. Writing-review and editing: MHH. All authors read and approved the final version of the manuscript.

## Conflicts of interest

The authors declare no conflicts of interest.

## Supplementary Material

RA-016-D6RA02423H-s001

RA-016-D6RA02423H-s002

## Data Availability

All data generated or analyzed during this study are included in the published article and its supplementary information (SI). Supplementary information is available. See DOI: https://doi.org/10.1039/d6ra02423h.
