# Supplementary material for: New benzochromene-based compounds as potential EGFR-TK inhibitors: synthesis, anti-proliferative activity, molecular docking studies, and ADME profiles
Source: RSC Adv. 2026 Apr 30;16(25):22450–64. doi: 10.1039/d6ra02423h (PMC13130050; doi:10.1039/d6ra02423h)
Supplement: RA-016-D6RA02423H-s001 [file RA-016-D6RA02423H-s001.pdf]

Supplementary Information (SI) for RSC Advances.
This journal is © The Royal Society of Chemistry 2026

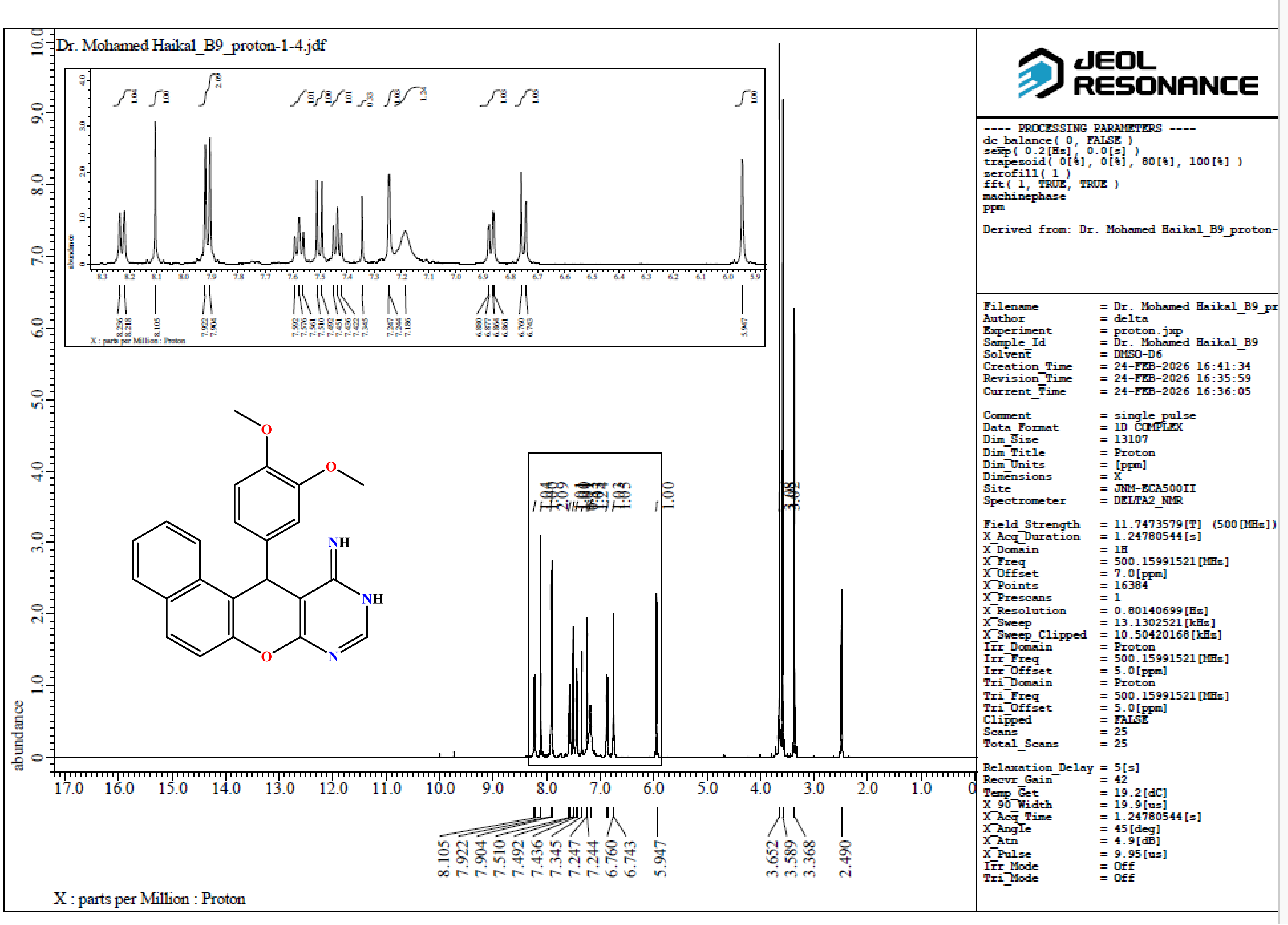

^{1}H-NMR spectrum (DMSO-d_6) of compound 2

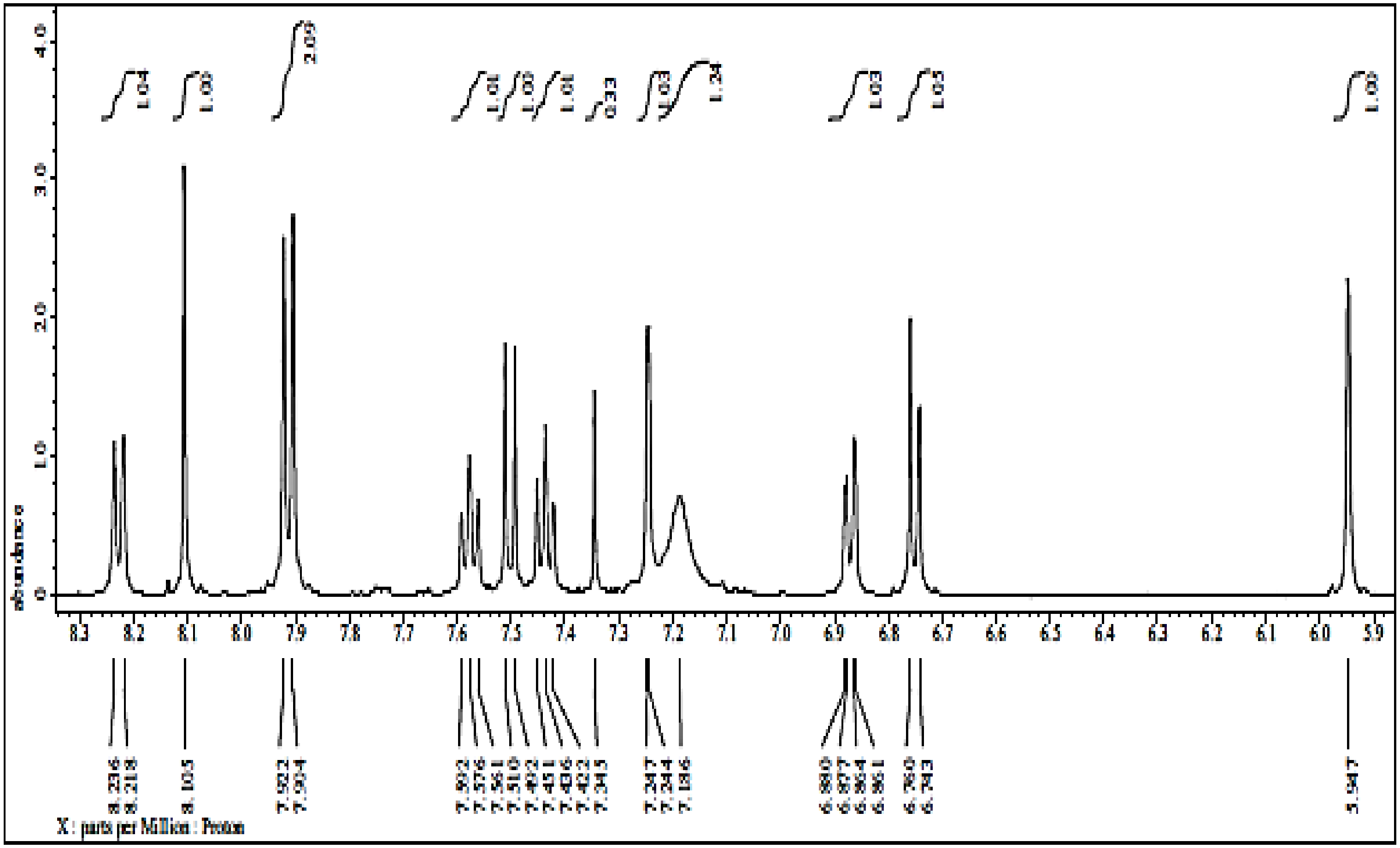

^{1}H-NMR spectrum (DMSO-d_6) of compound 2

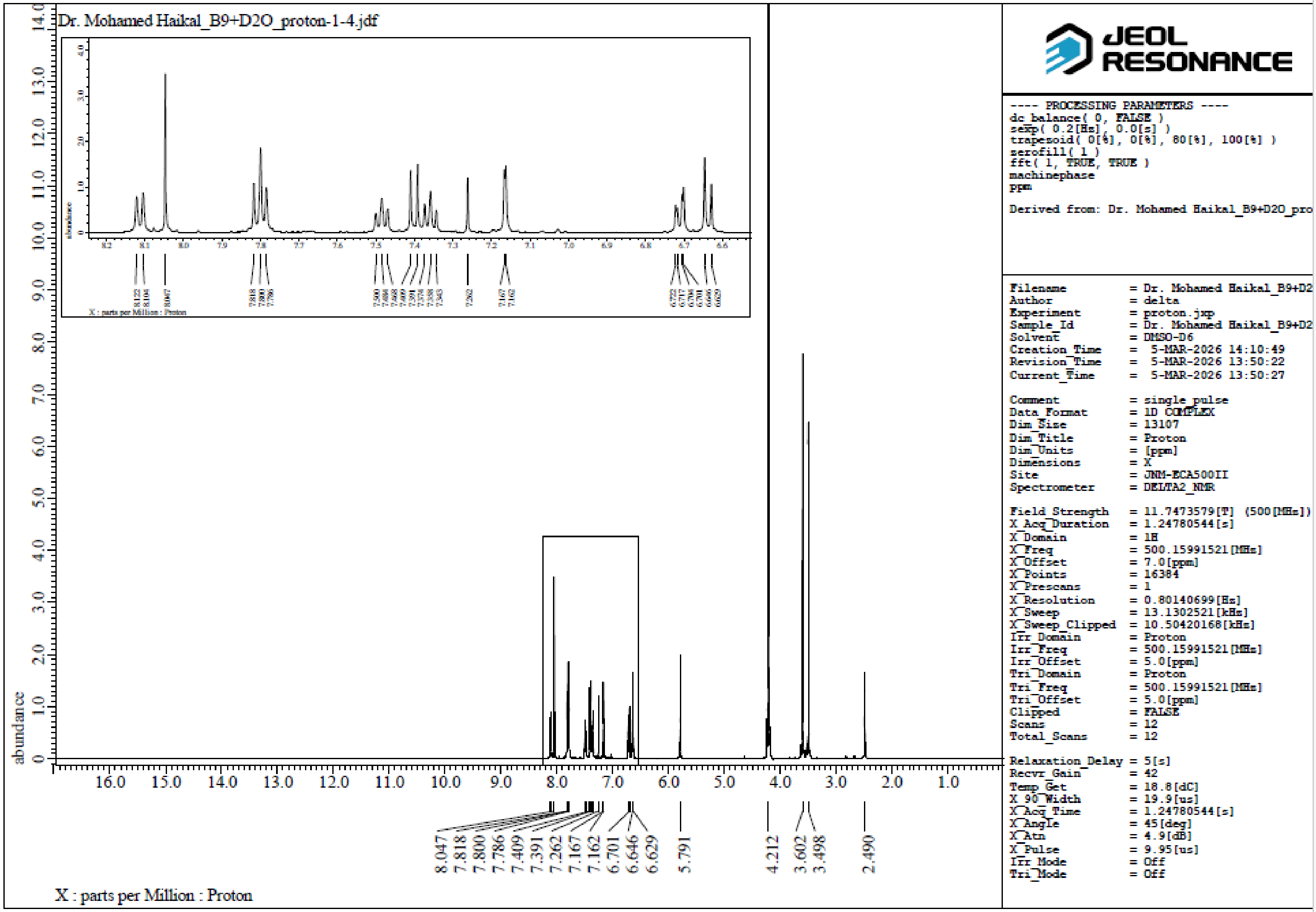

^{1}H-NMR spectrum (DMSO-d_6+ D_2O) of compound 2

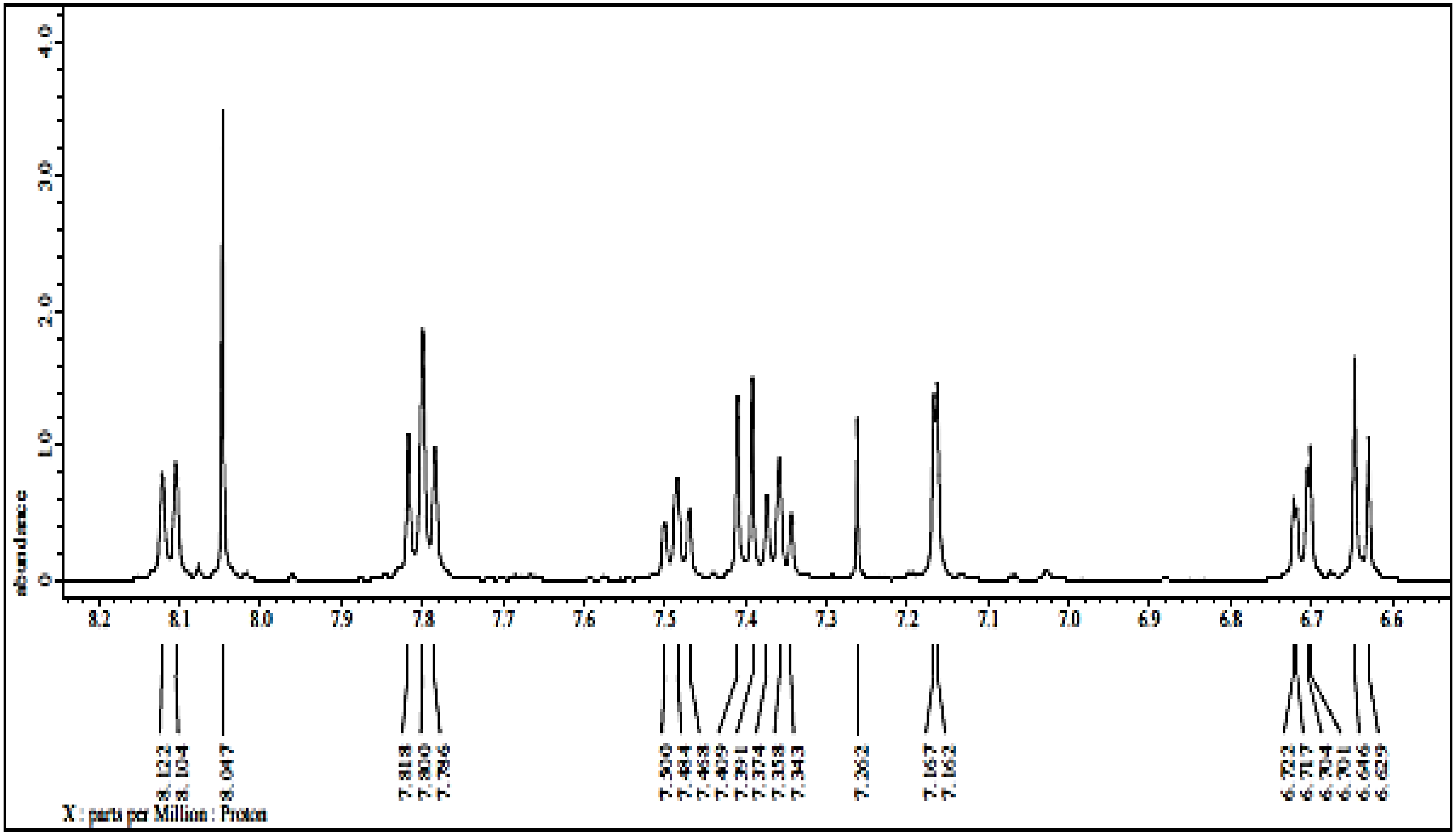

^{1}H-NMR spectrum (DMSO-d_6+ D_2O) of compound 2

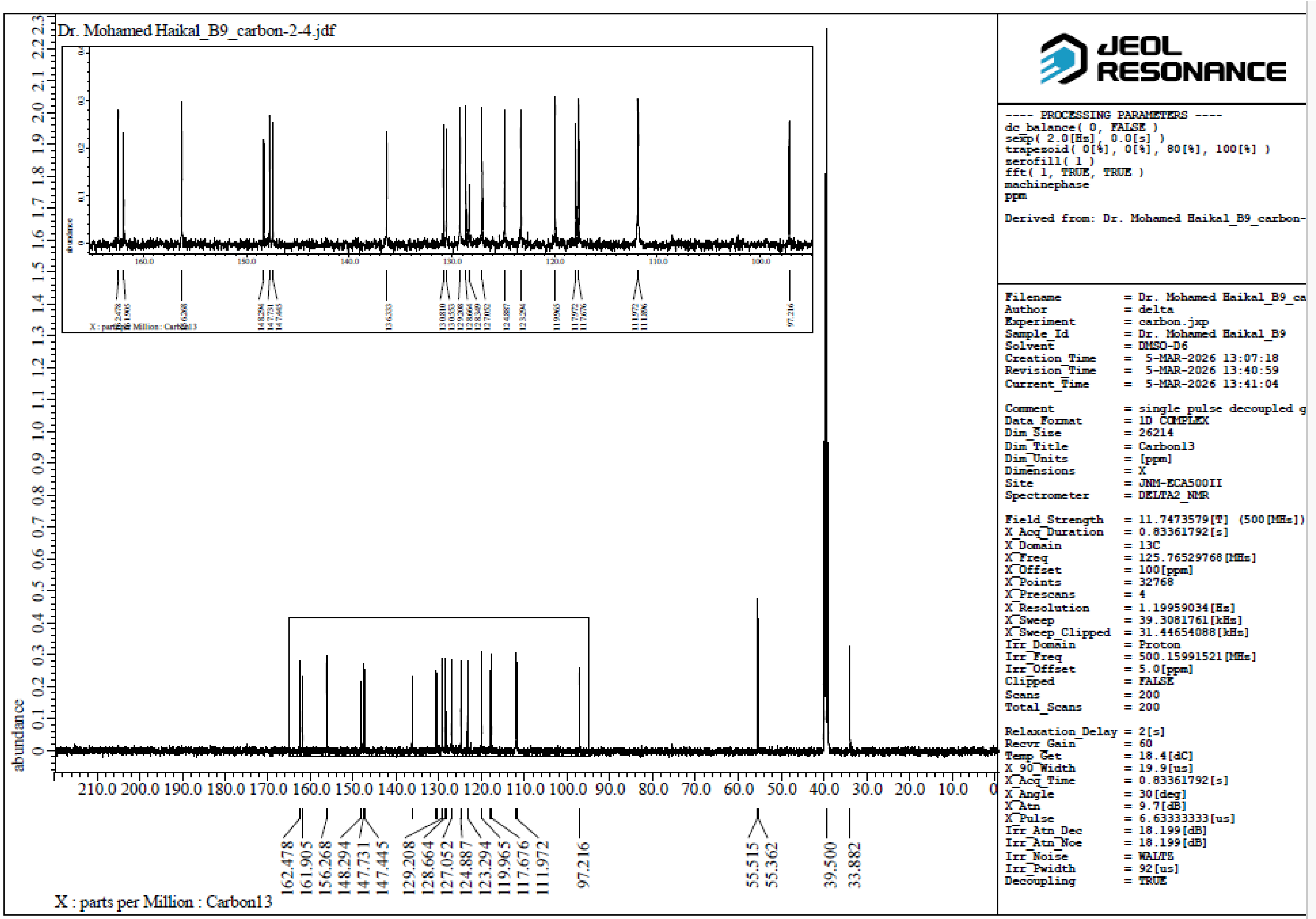

^{13}C-NMR spectrum (DMSO-d_6) of compound 2

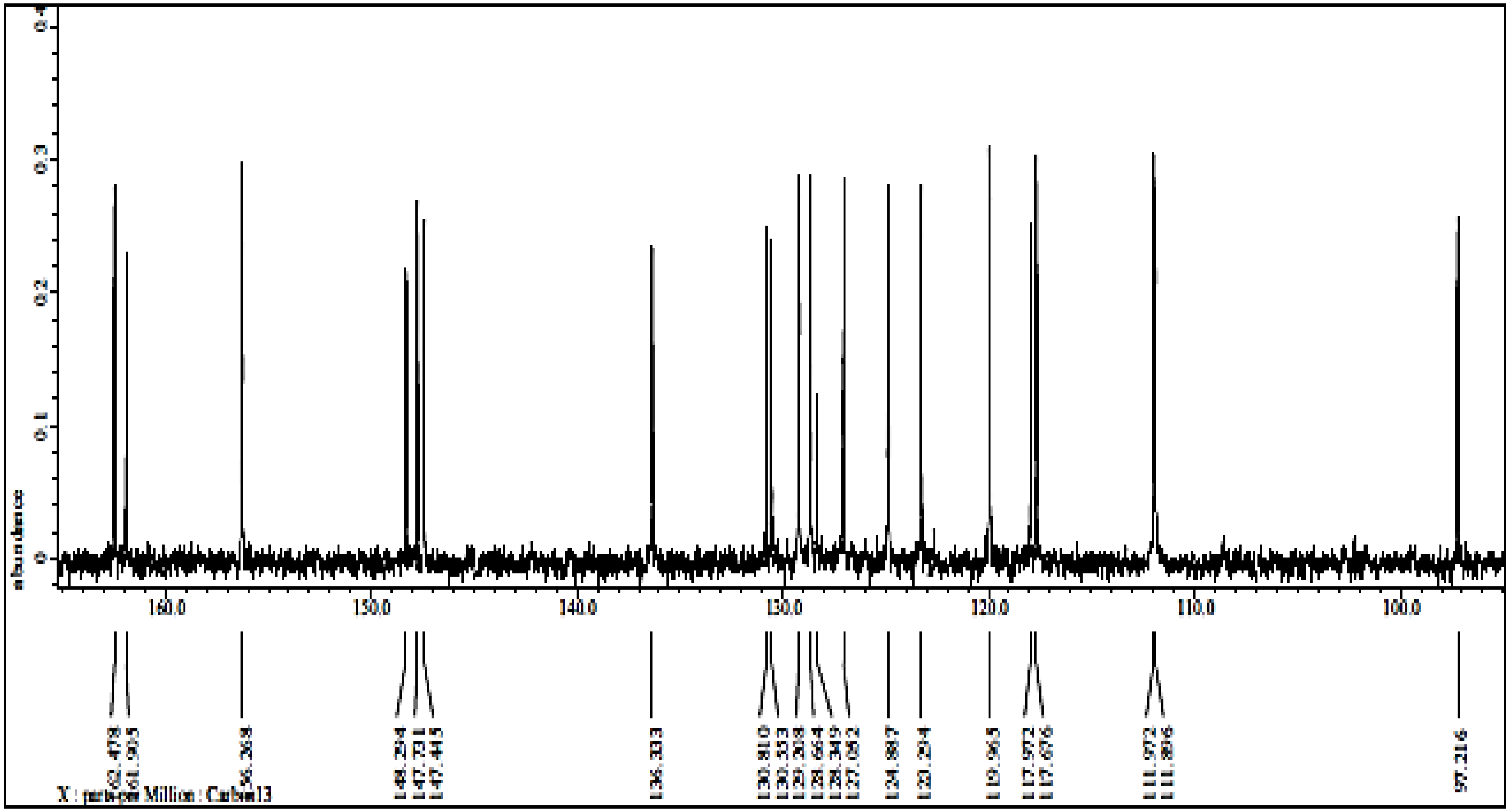

^{13}C-NMR spectrum (DMSO-d_6) of compound 2

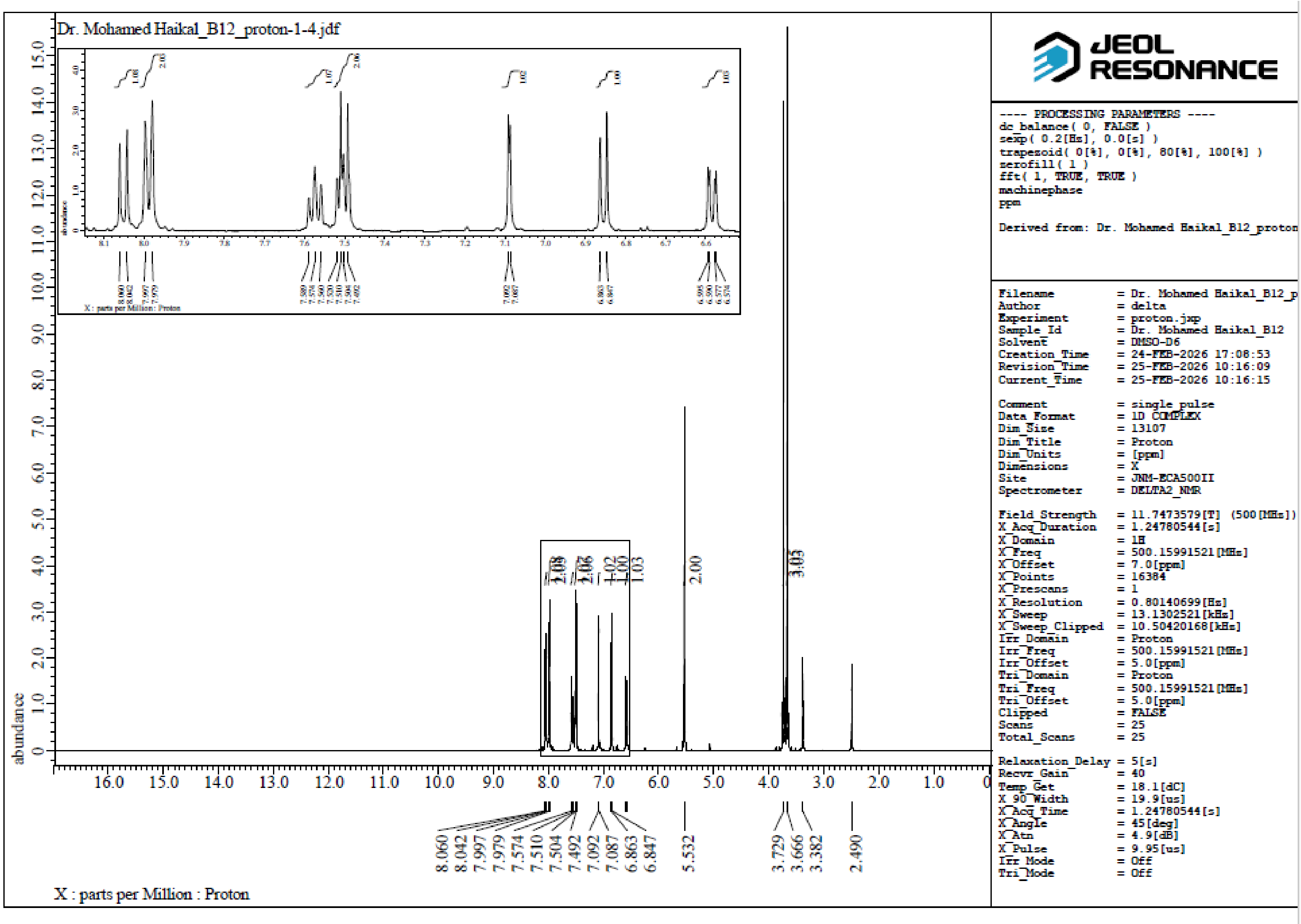

^{1}H-NMR spectrum (DMSO-d_6) of compound 3

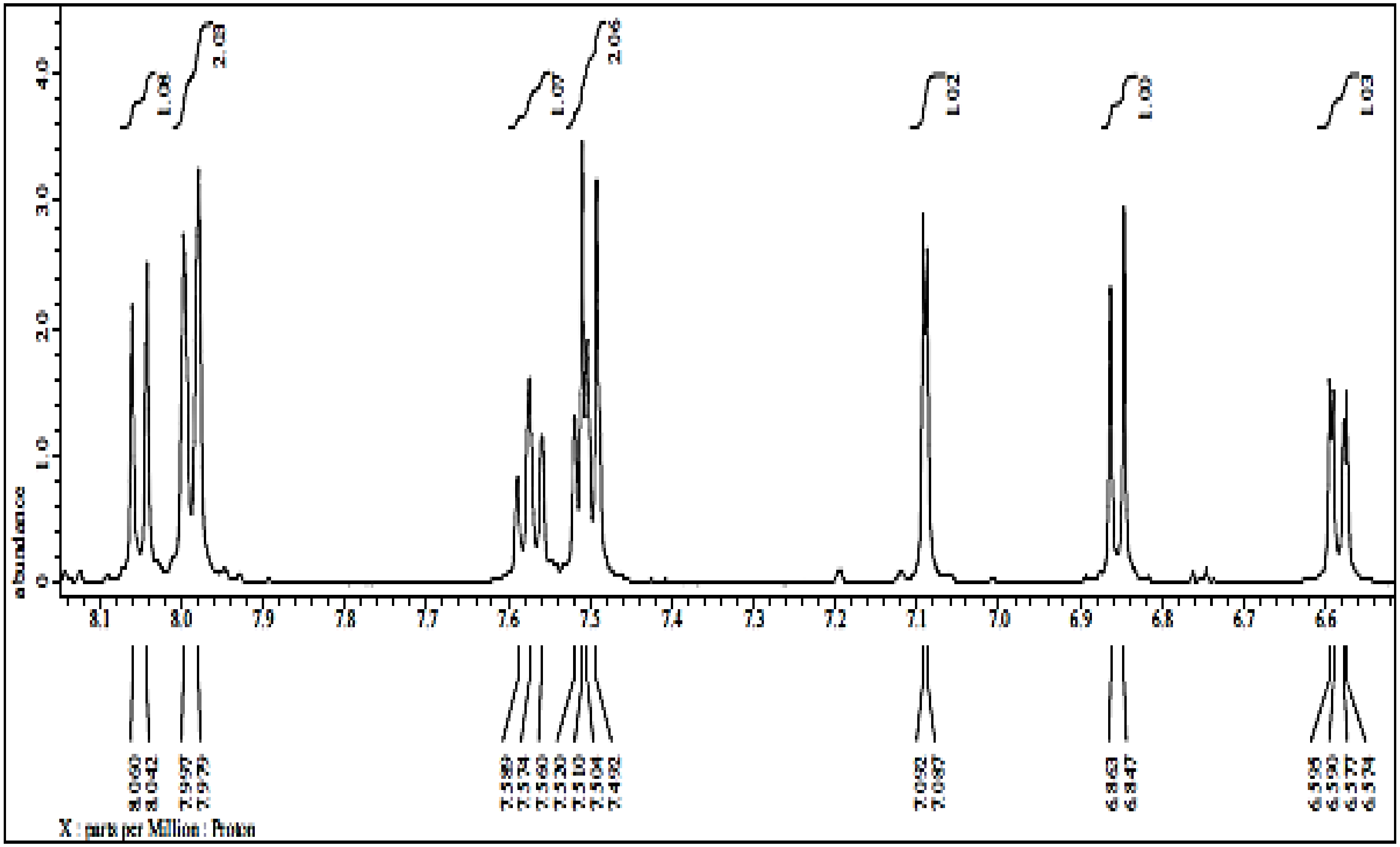

^{1}H-NMR spectrum (DMSO-d_6) of compound 3

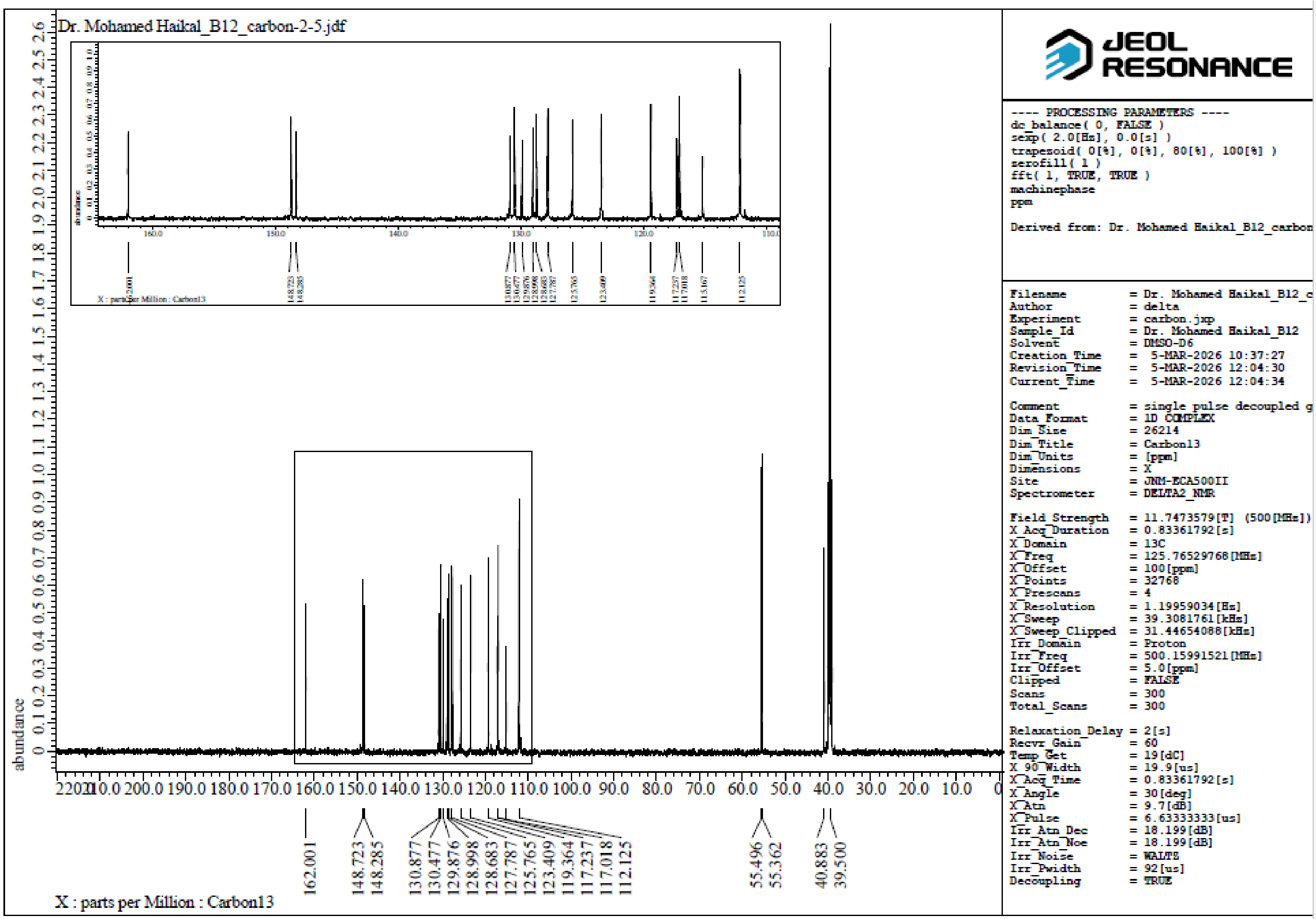

^{13}C-NMR spectrum (DMSO-d_6) of compound 3

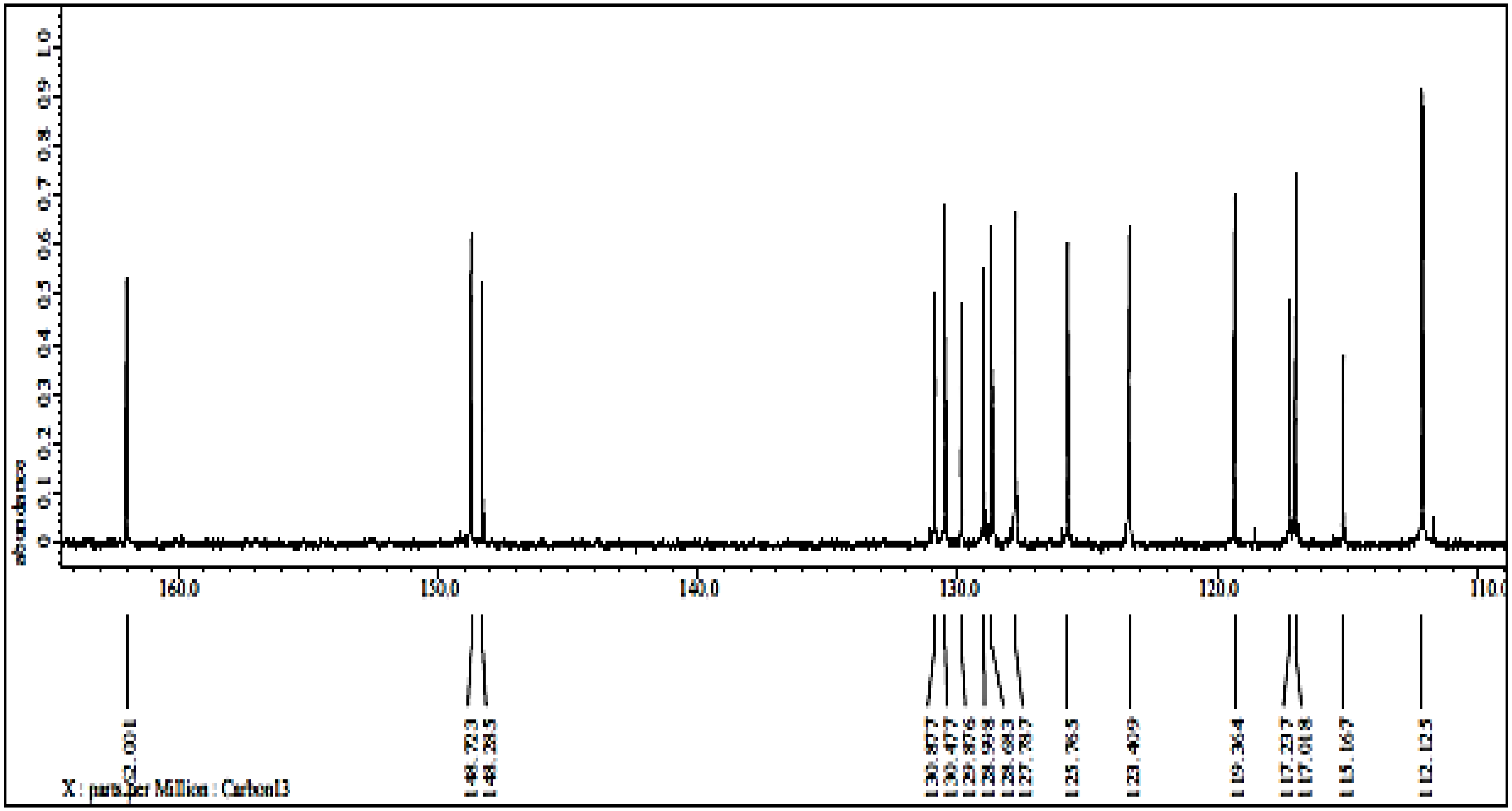

^{13}C-NMR spectrum (DMSO-d_6) of compound 3

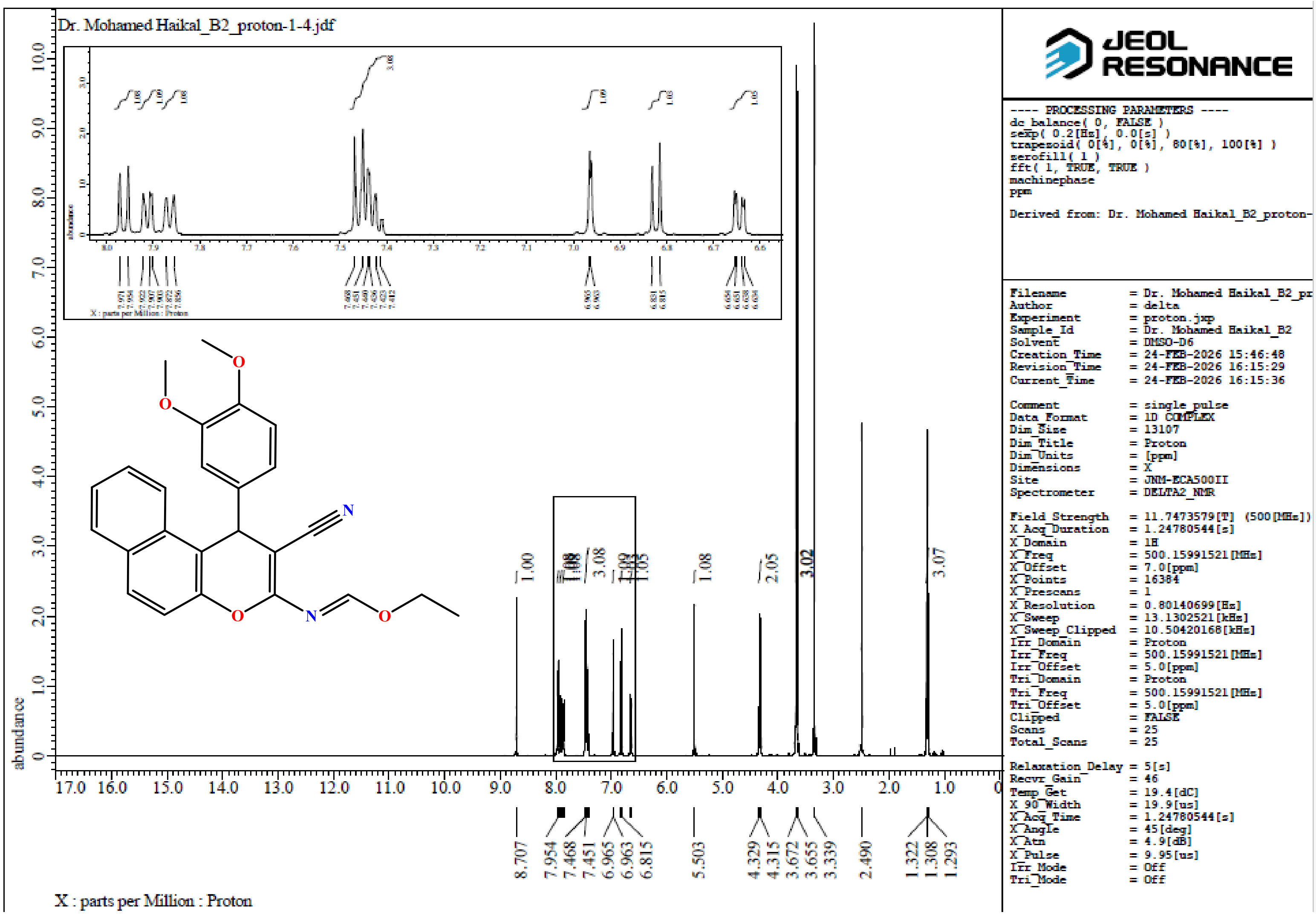

^{1}H-NMR spectrum (DMSO-d_6) of compound 4

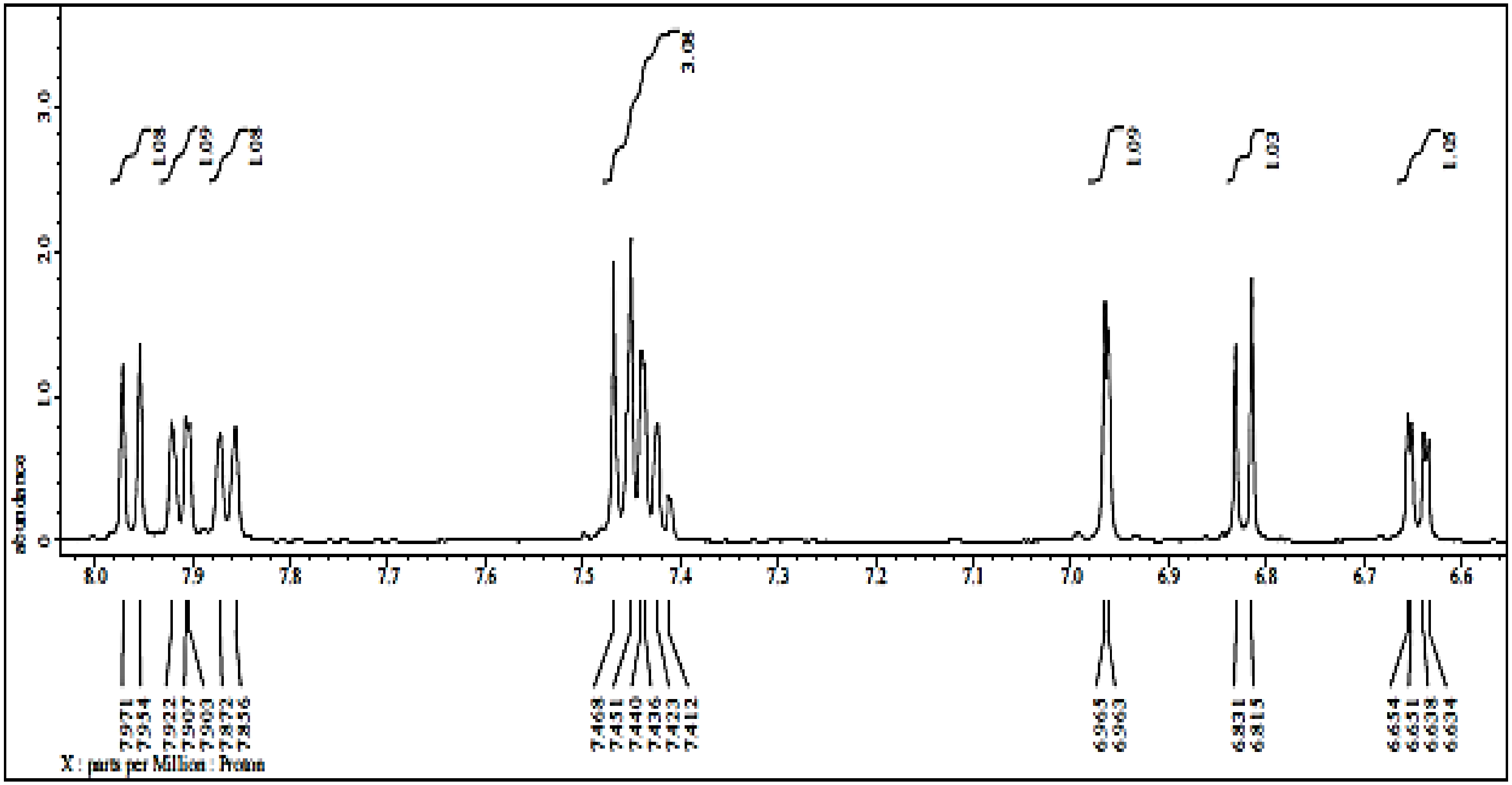

^{1}H-NMR spectrum (DMSO-d_6) of compound 4

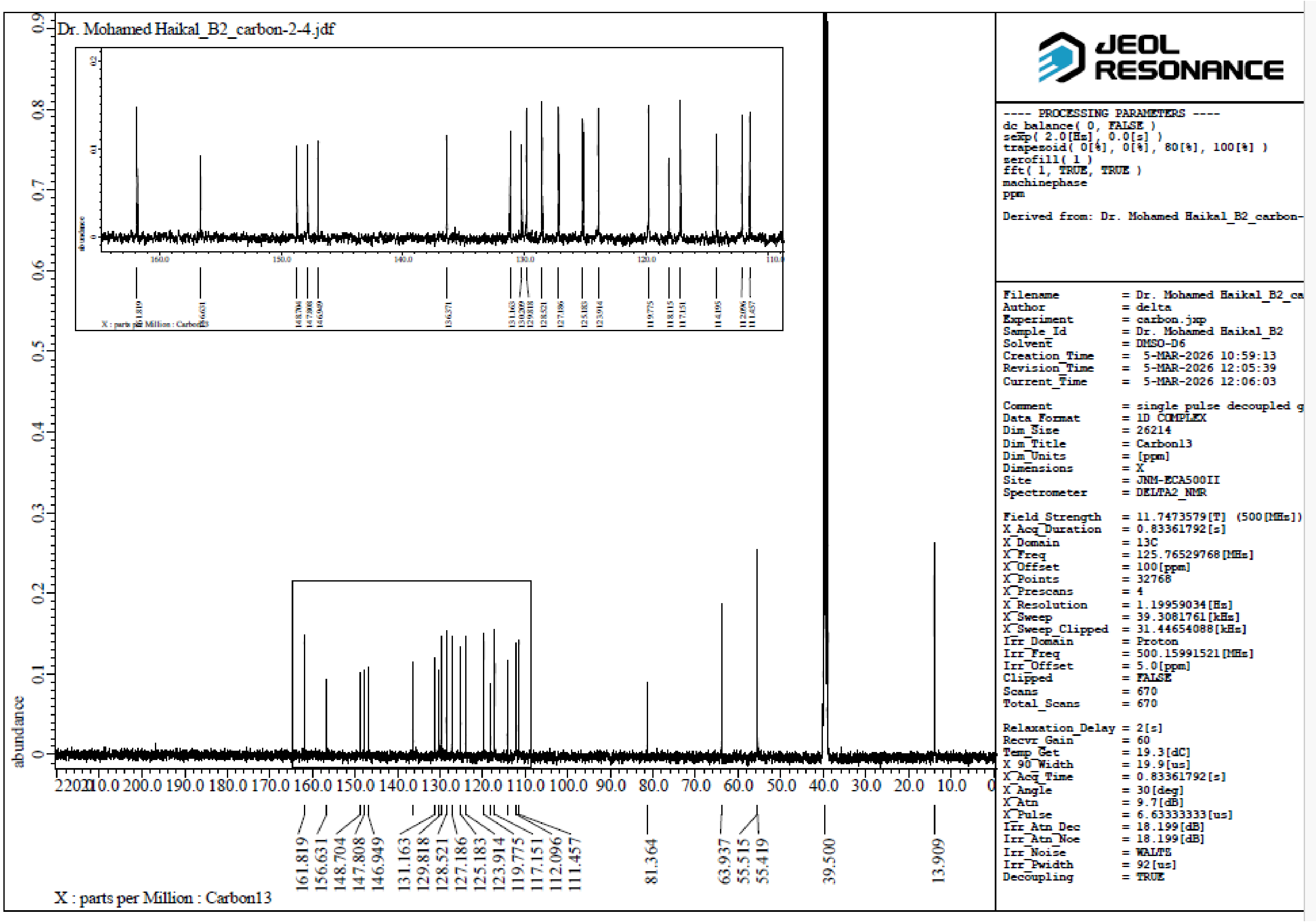

^{13}C-NMR spectrum (DMSO-d_6) of compound 4

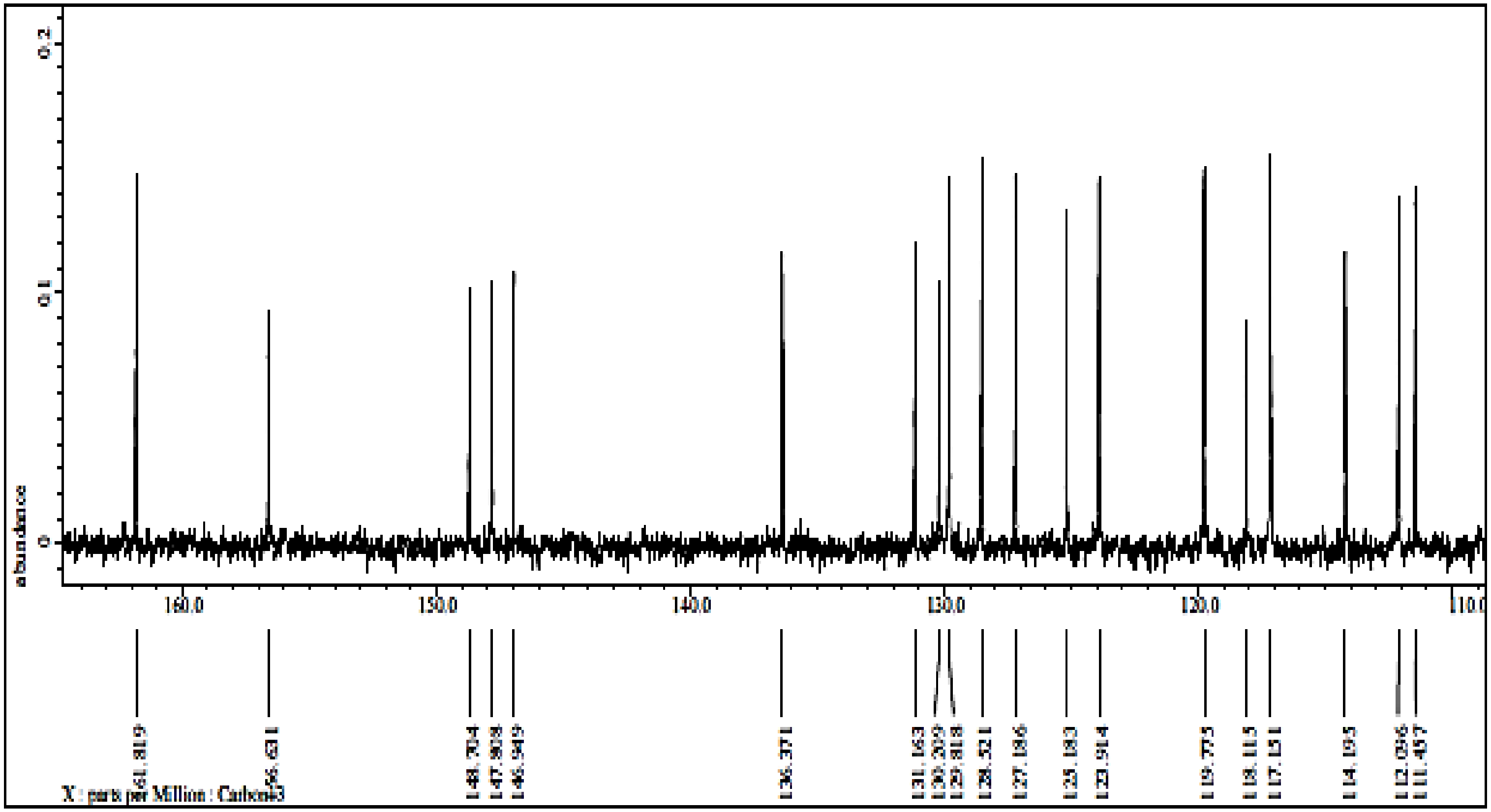

^{13}C-NMR spectrum (DMSO-d_6) of compound 4

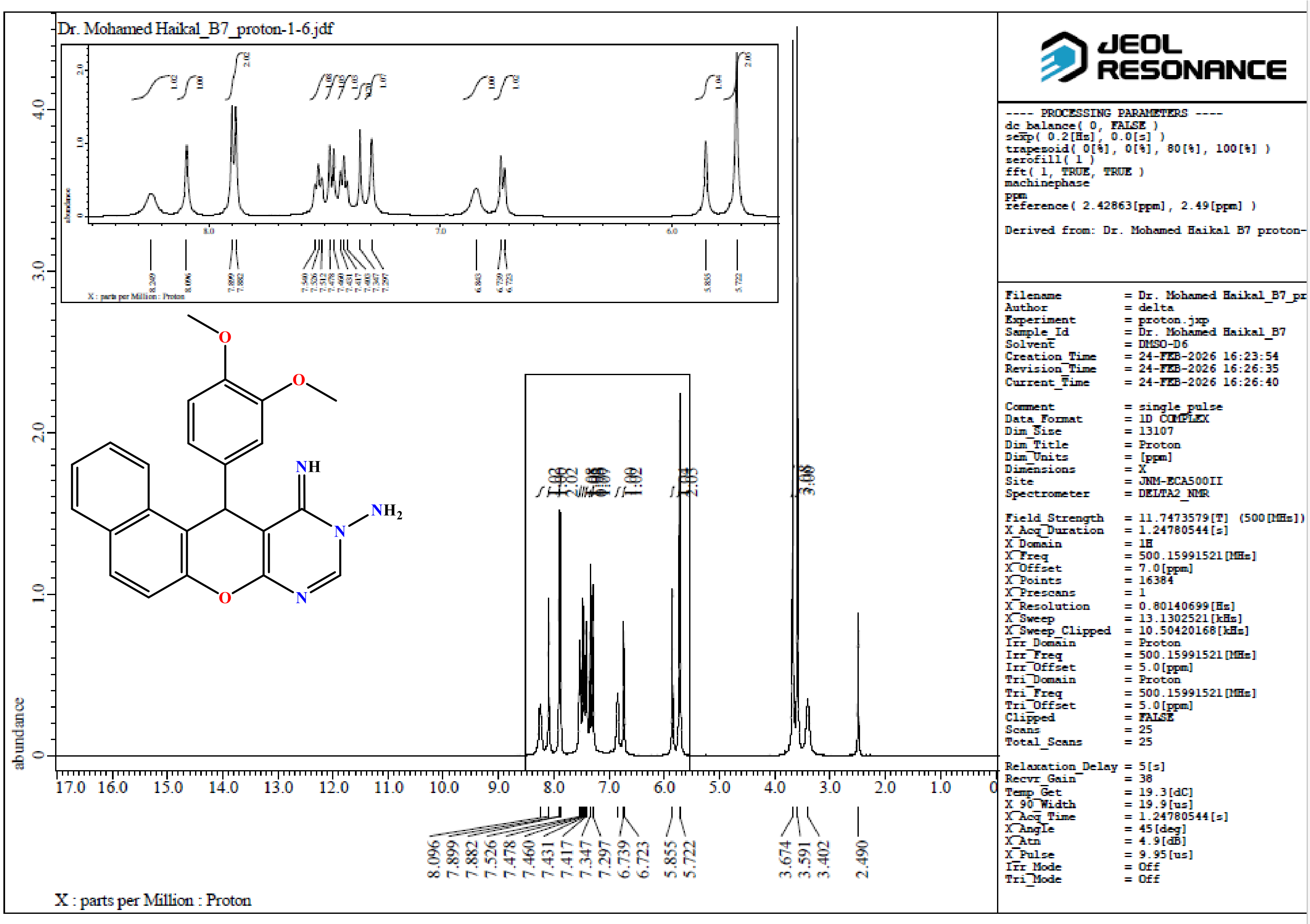

^{1}H-NMR spectrum (DMSO-d_6) of compound 5

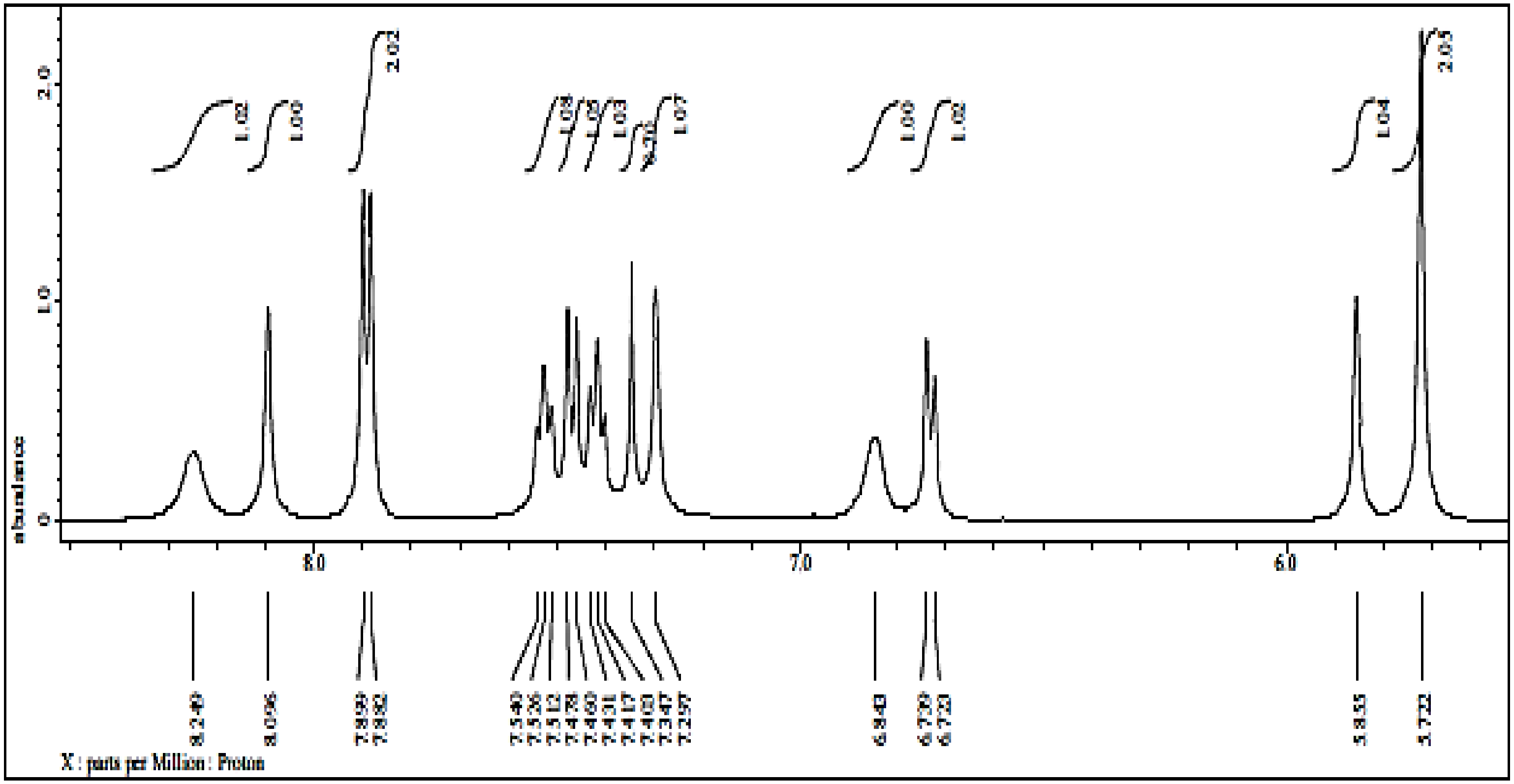

^{1}H-NMR spectrum (DMSO-d_6) of compound 5

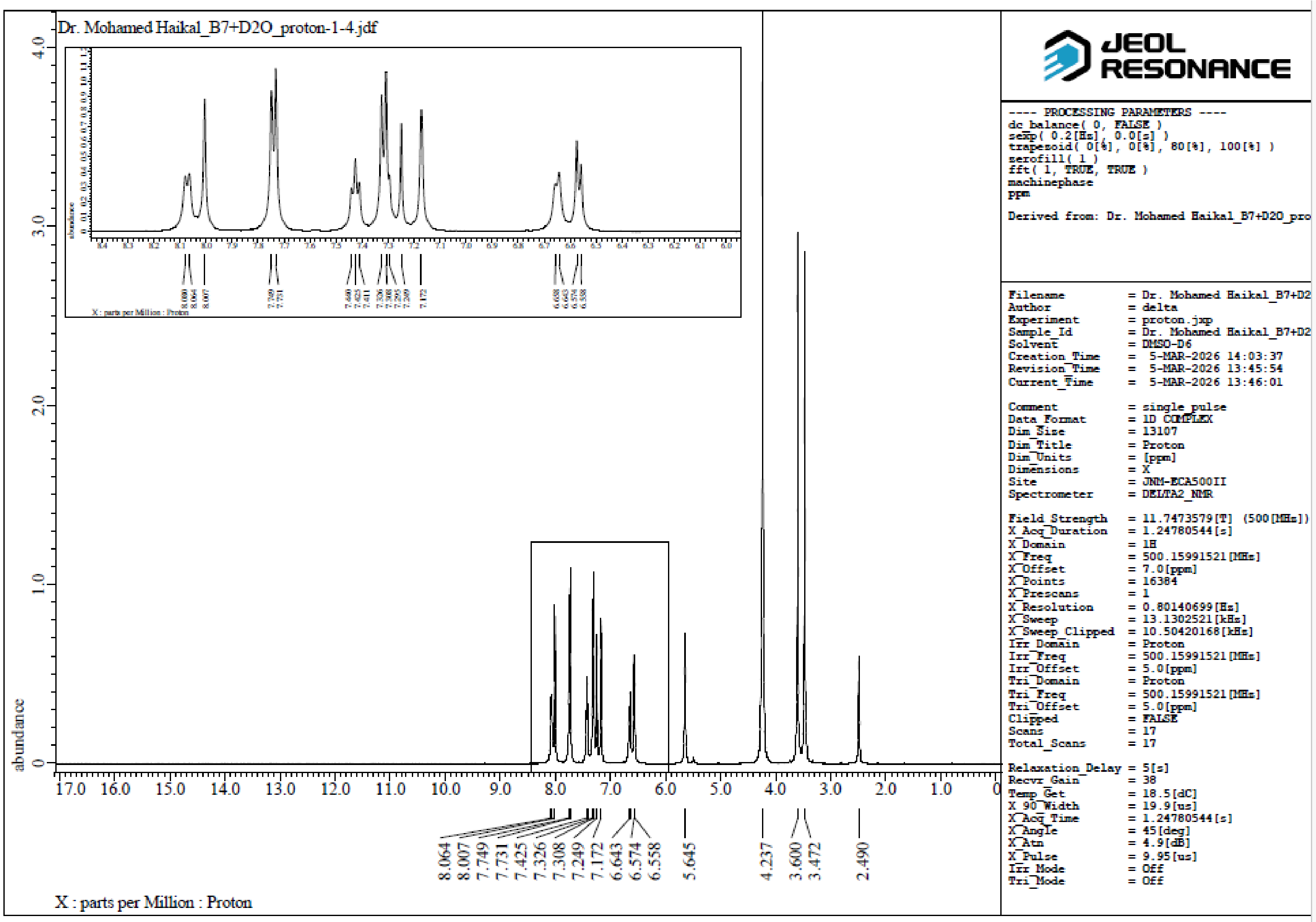

^{1}H-NMR spectrum (DMSO-d_6+ D_2O) of compound 5

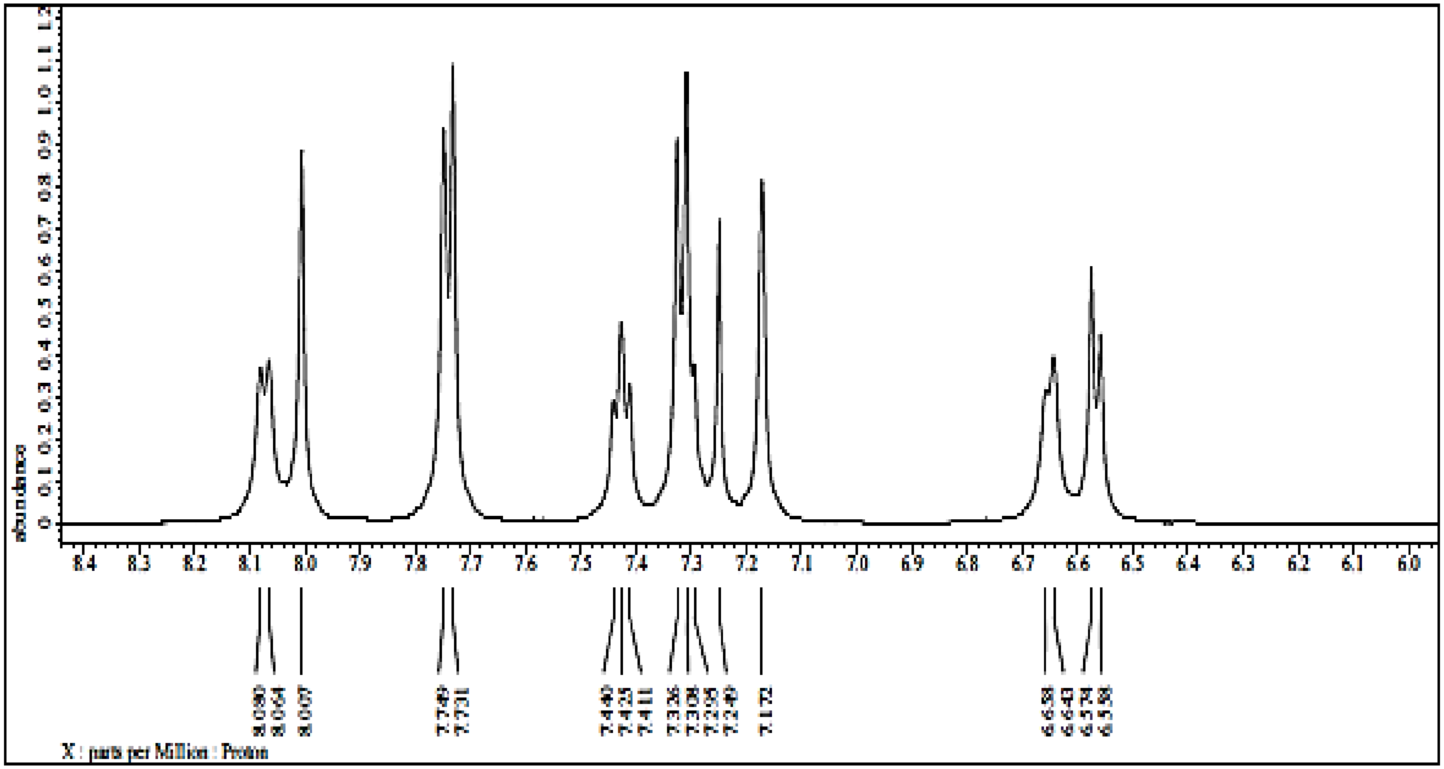

^{1}H-NMR spectrum (DMSO-d_6+ D_2O) of compound 5

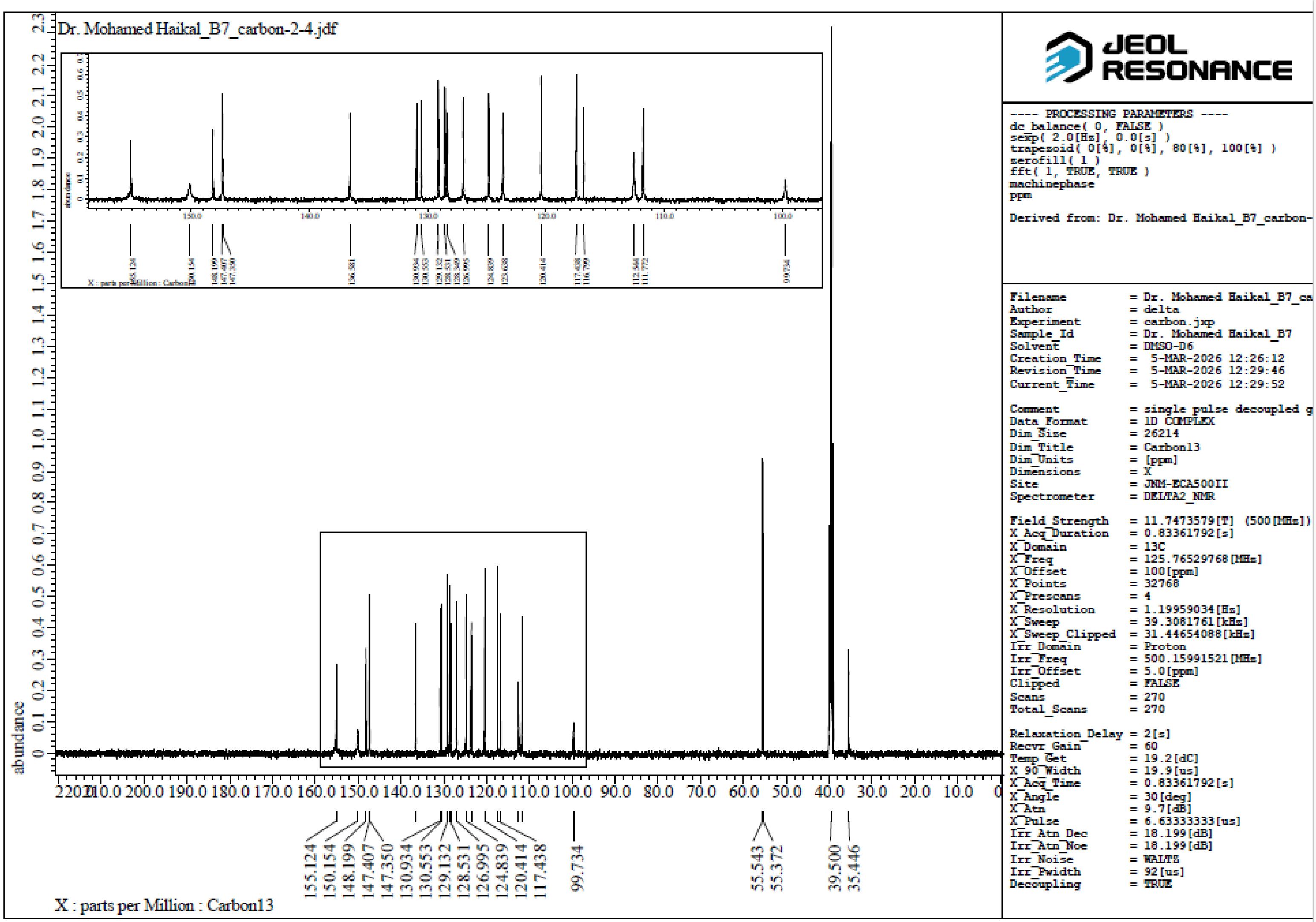

^{13}C-NMR spectrum (DMSO-d_6) of compound 5

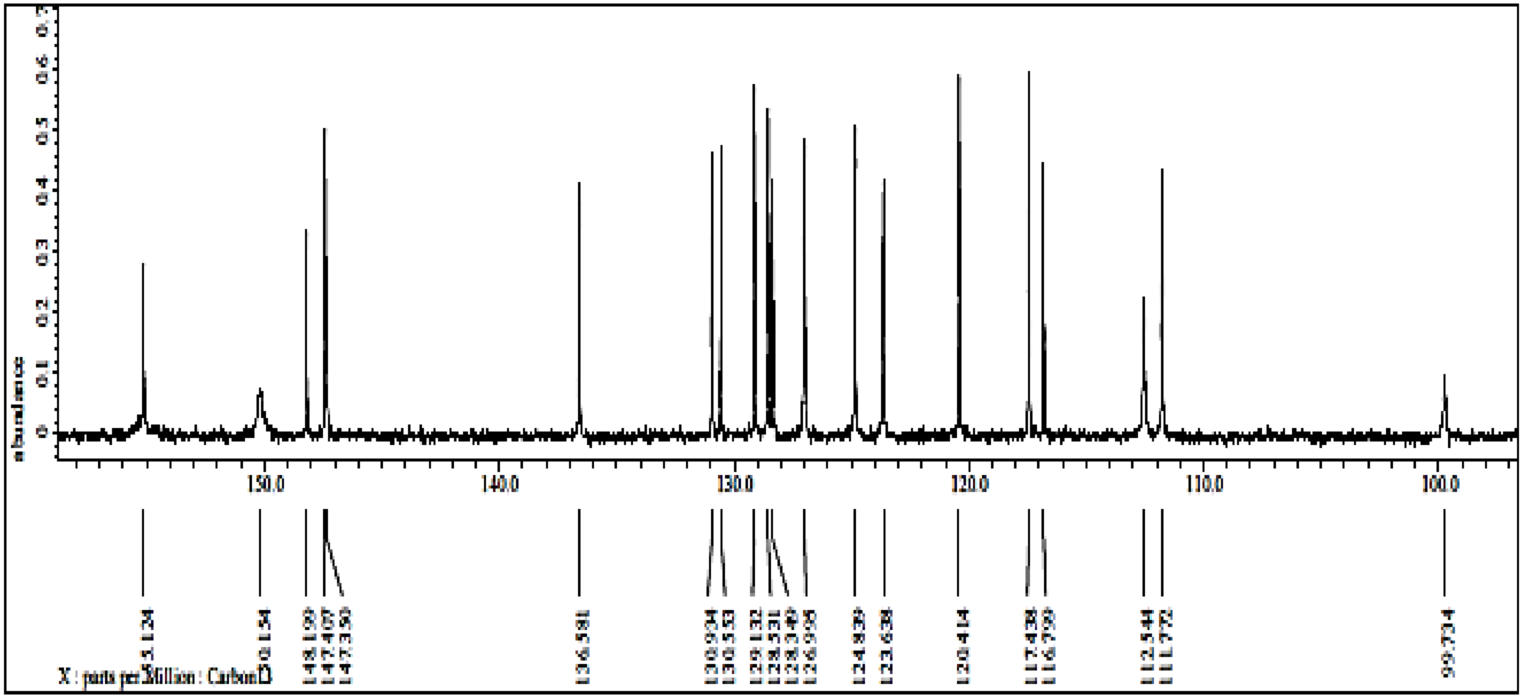

^{13}C-NMR spectrum (DMSO-d_6) of compound 5

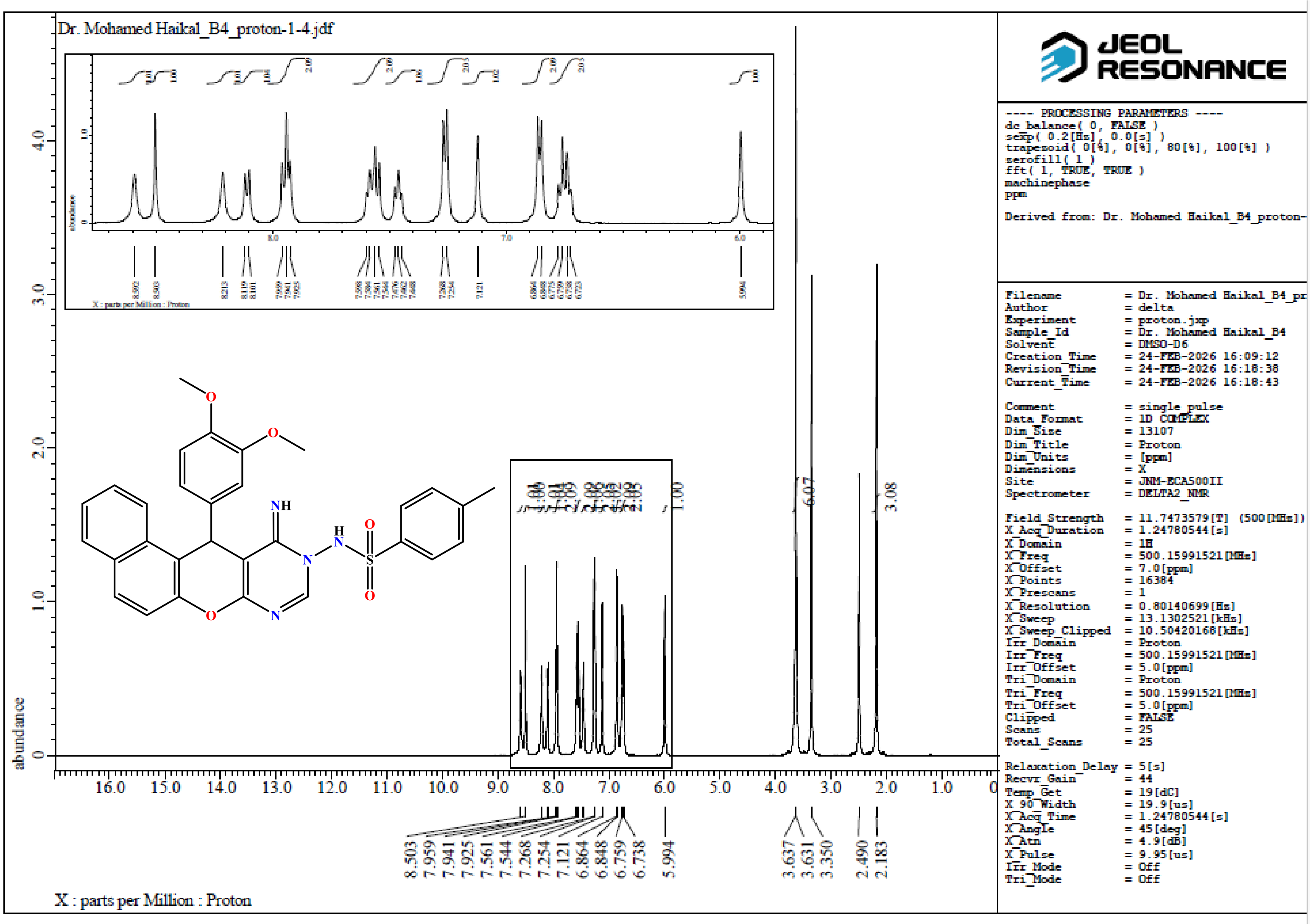

^{1}H-NMR spectrum (DMSO-d_6) of compound 6

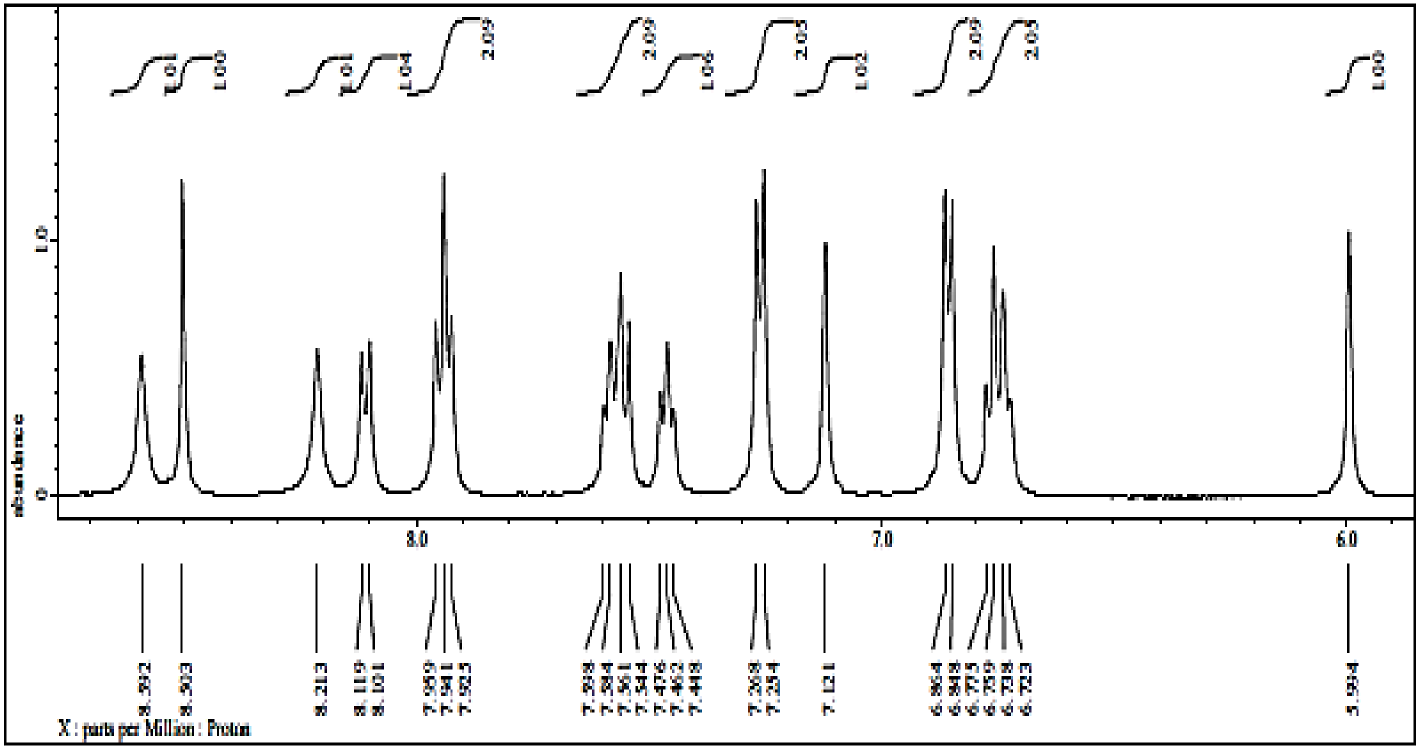

^{1}H-NMR spectrum (DMSO-d_6) of compound 6

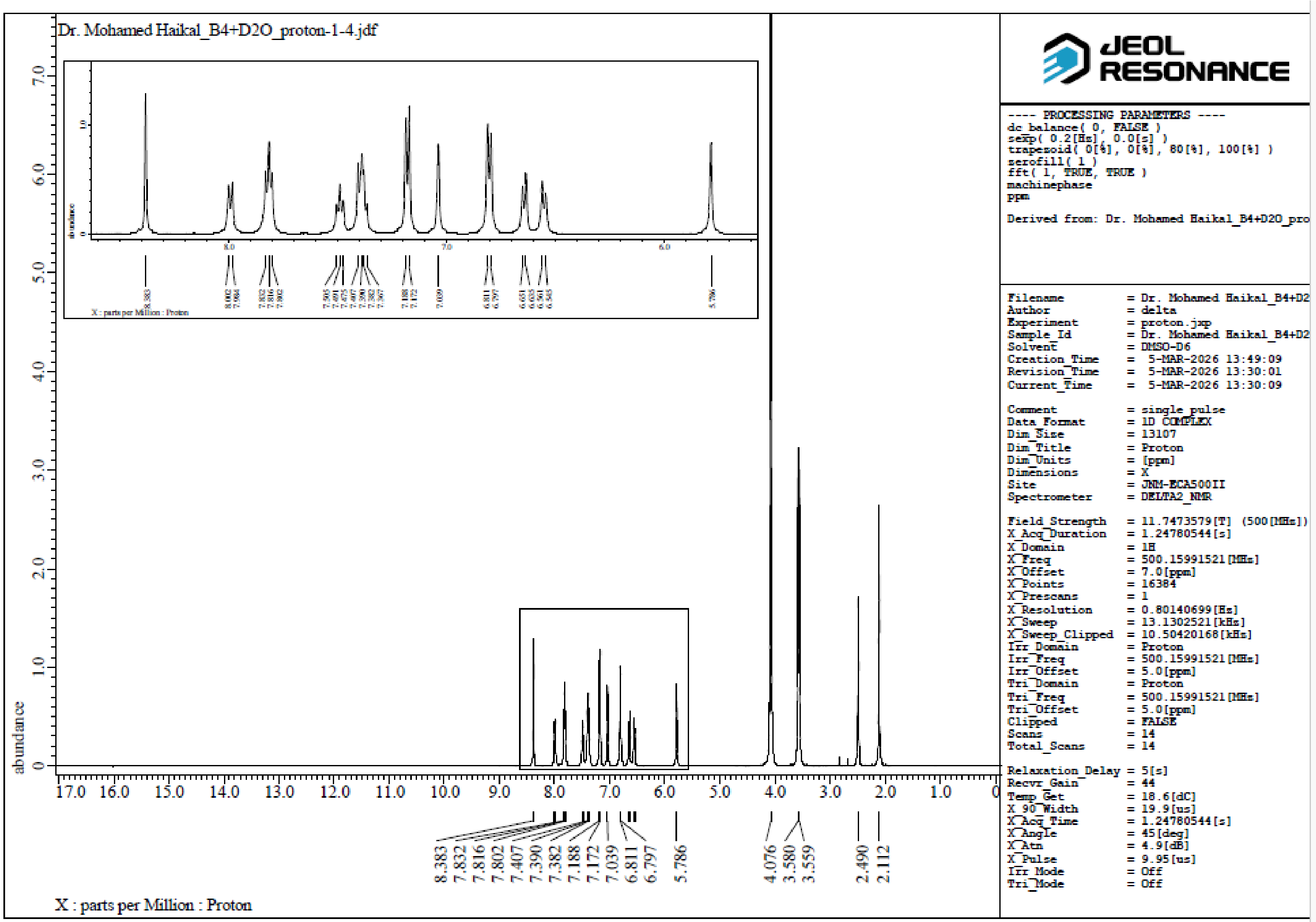

^{1}H-NMR spectrum (DMSO-*d*6+D_2O) of compound 6

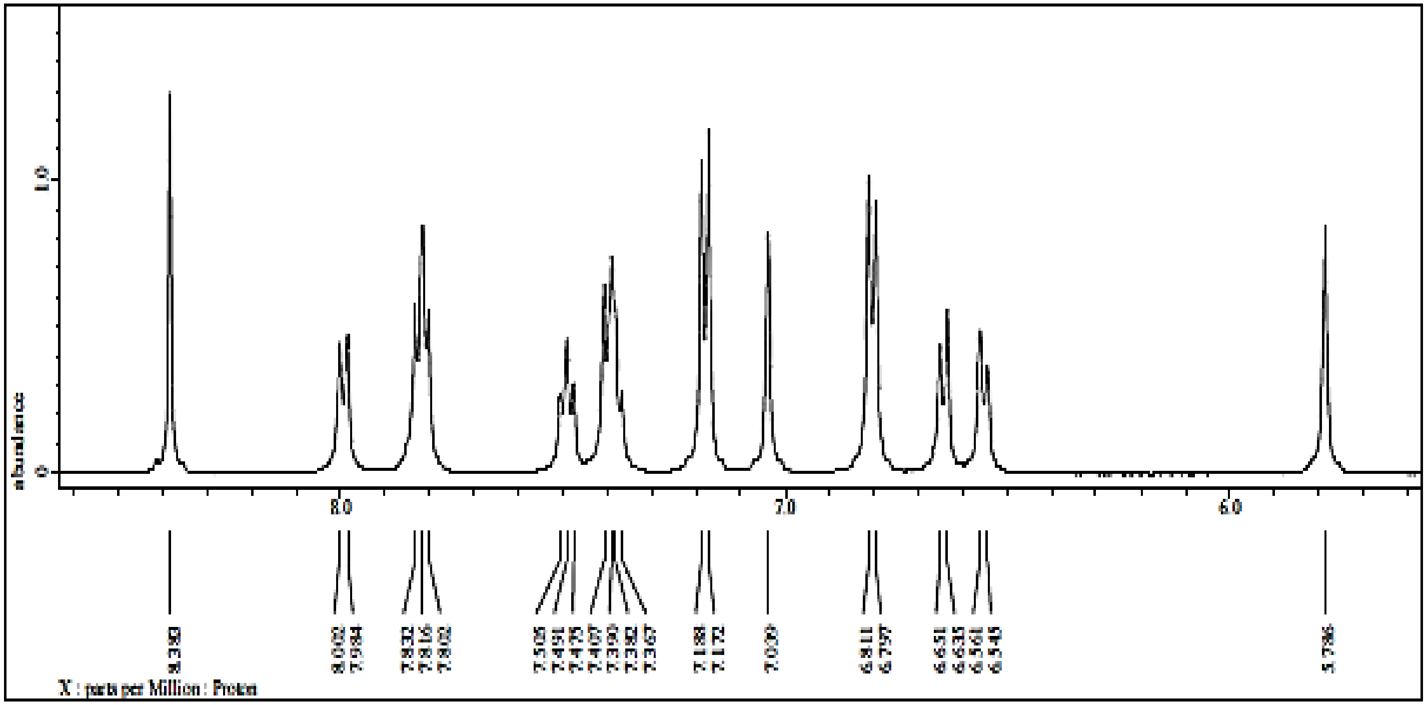

^{1}H-NMR spectrum (DMSO-*d*6+D_2O) of compound 6

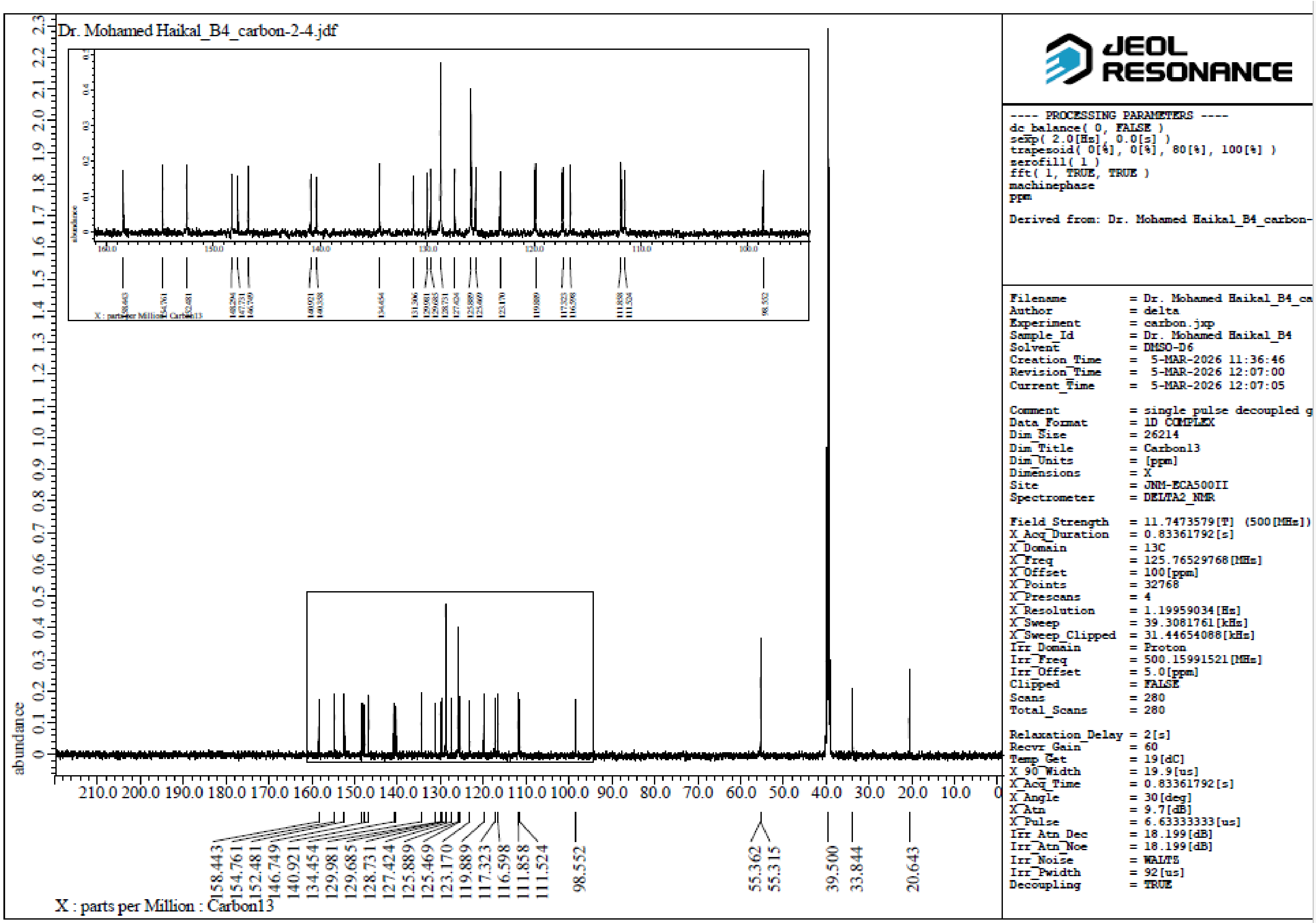

^{13}C-NMR spectrum (DMSO-d_6) of compound 6

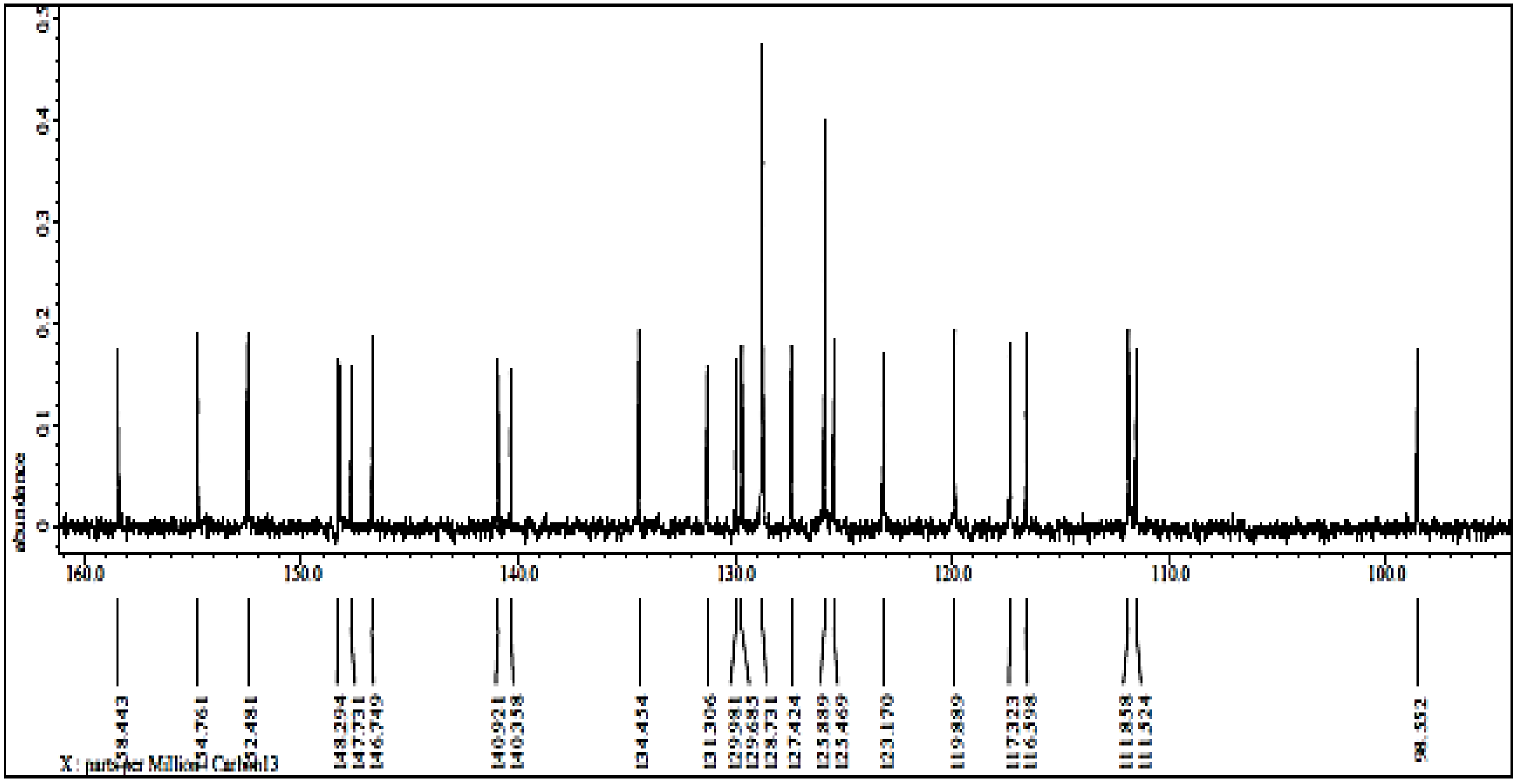

^{13}C-NMR spectrum (DMSO-d_6) of compound 6

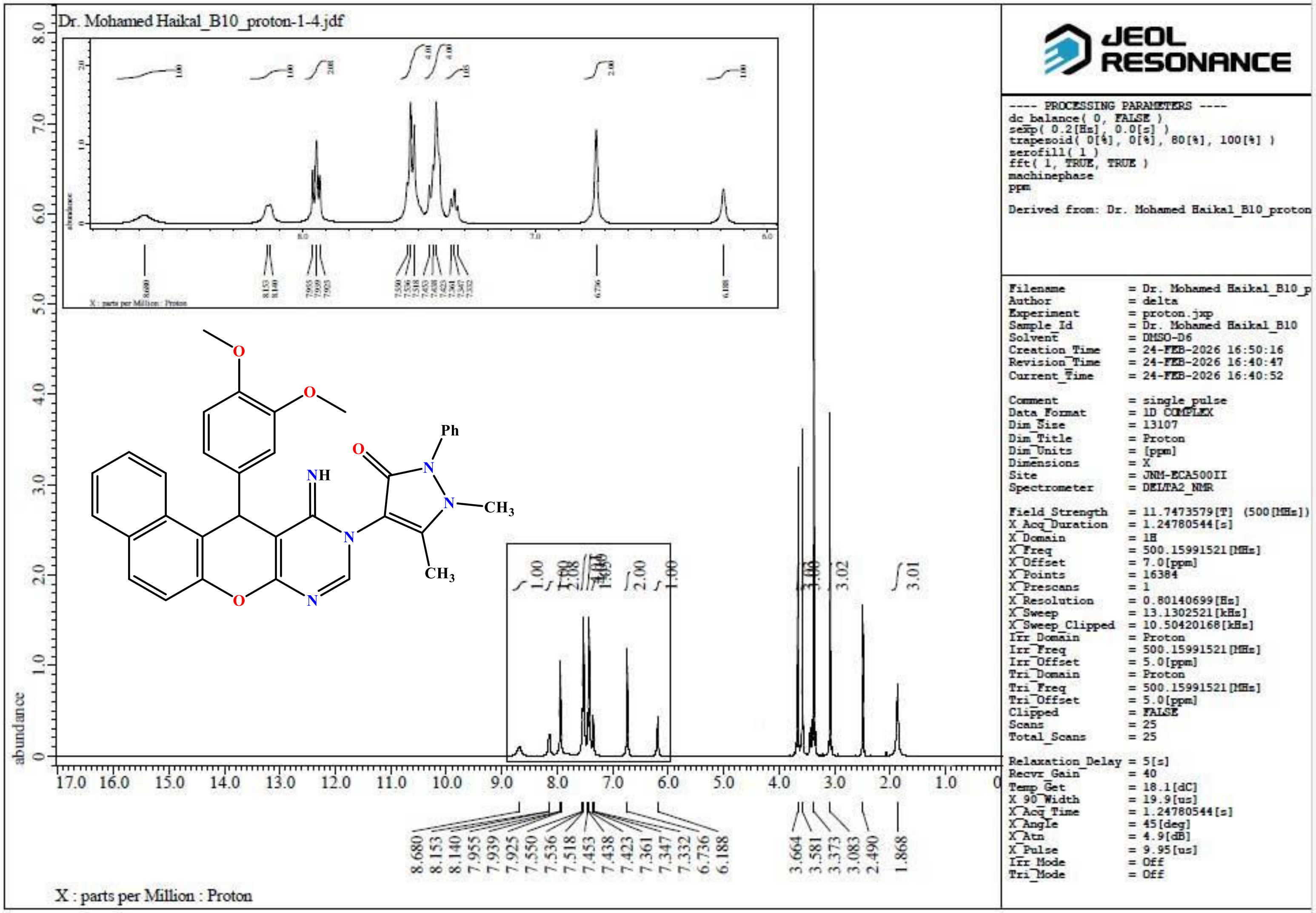

¹H-NMR spectrum (DMSO-d_6) of compound 7

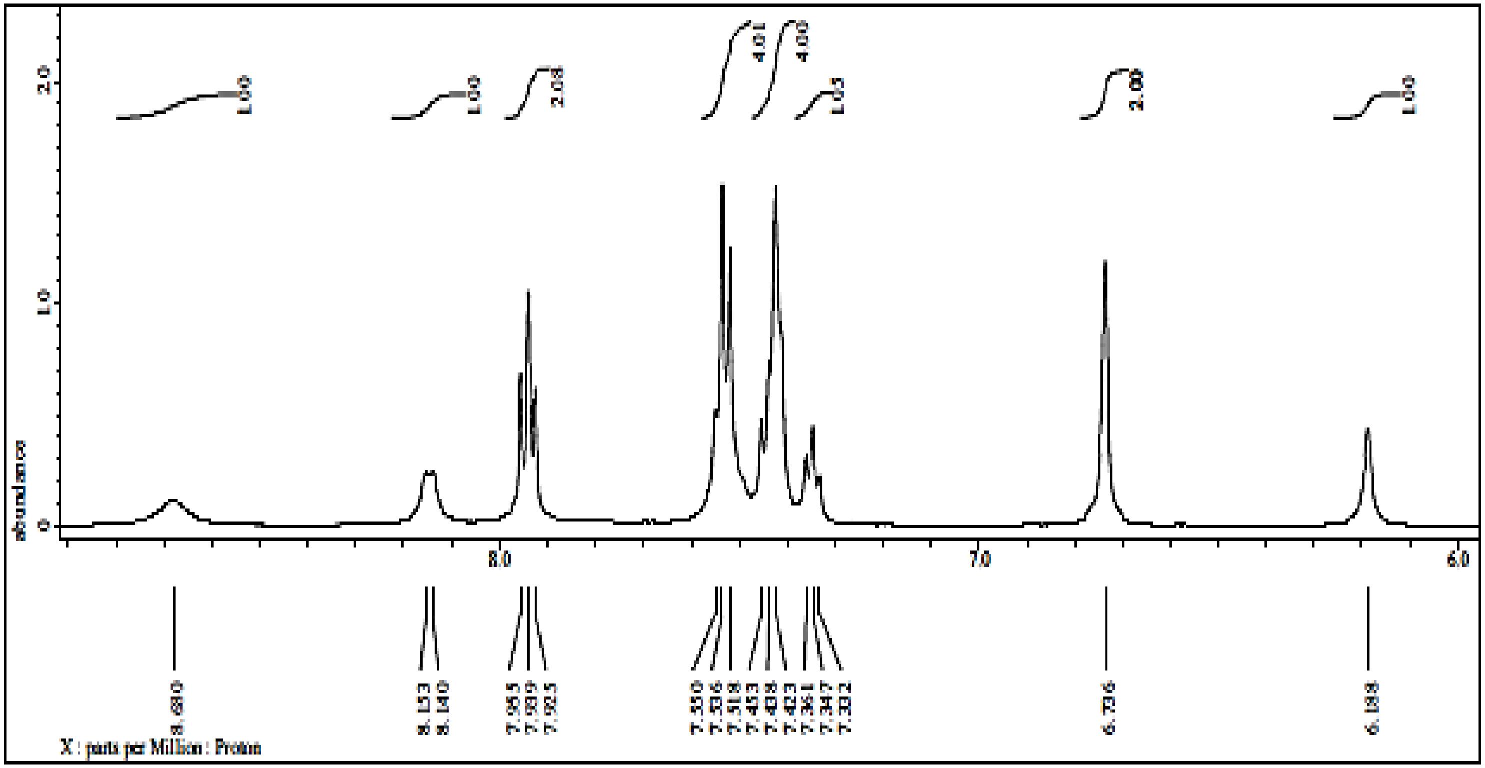

^{1}H-NMR spectrum (DMSO-d_6) of compound 7

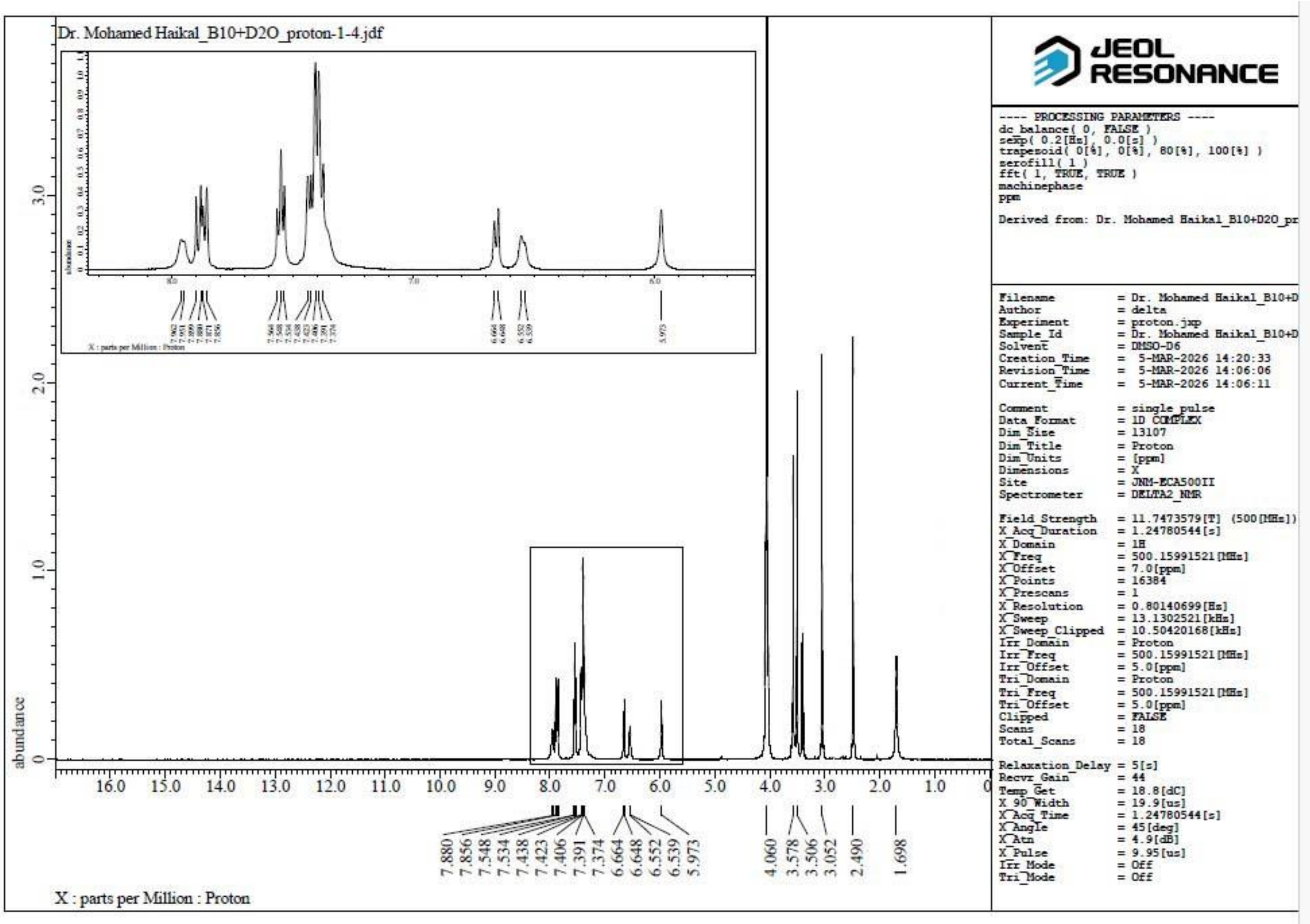

^{1}H-NMR spectrum (DMSO-*d*6+D_2O) of compound 7

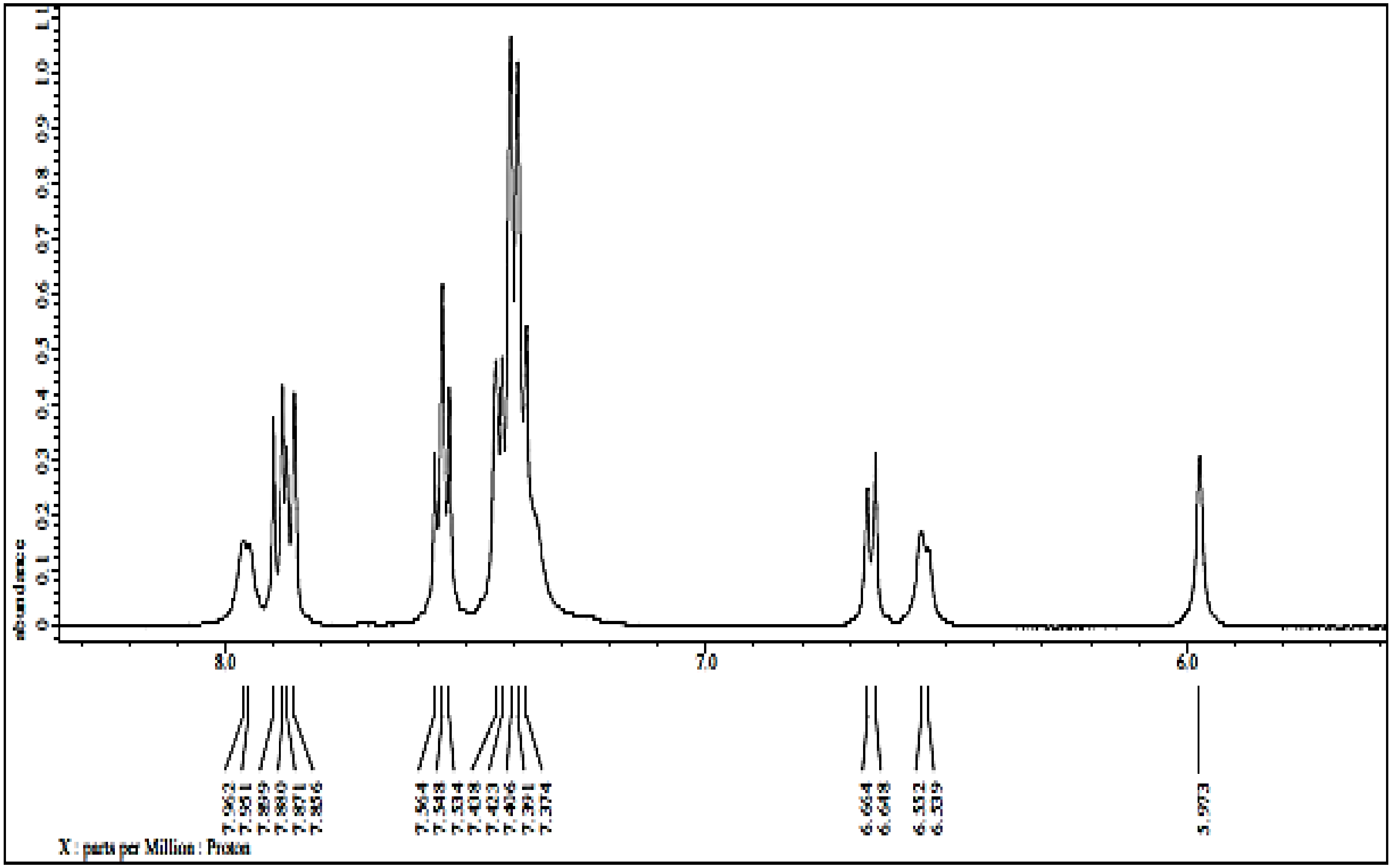

^{1}H-NMR spectrum (DMSO-*d*6+D_2O) of compound 7

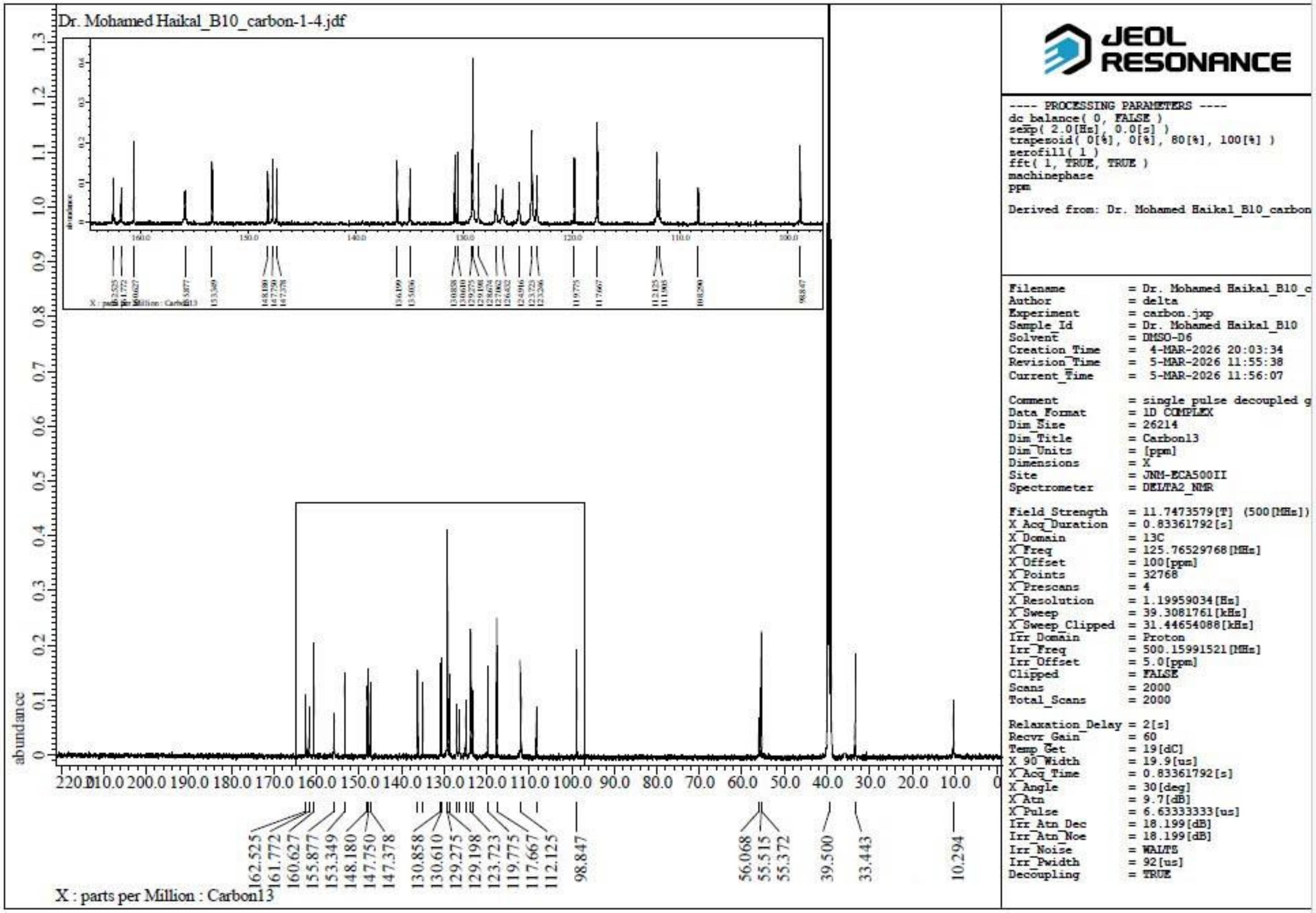

^{13}C-NMR spectrum (DMSO-d_6) of compound 7

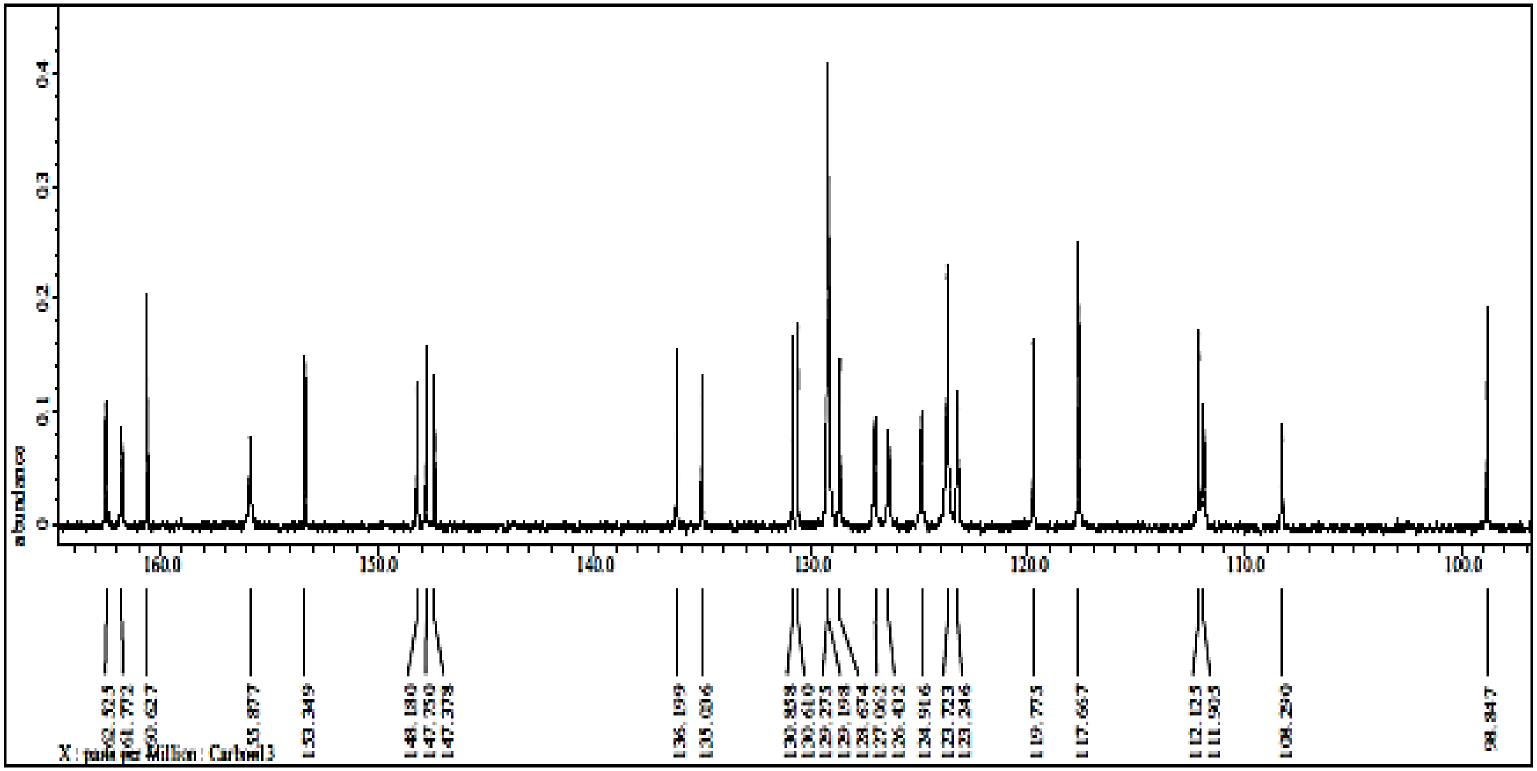

^{13}C-NMR spectrum (DMSO-*d_6*) of compound 7

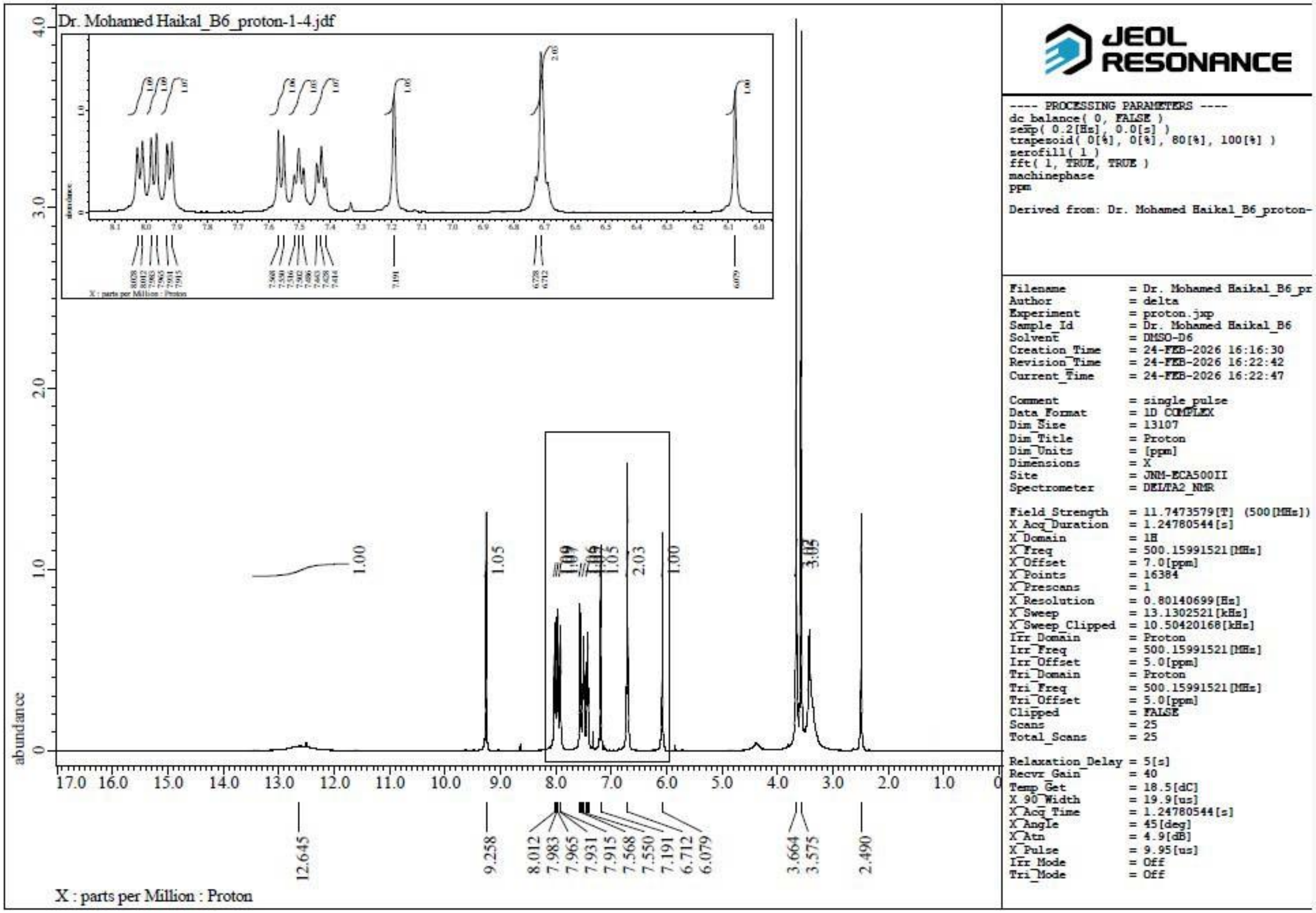

^{1}H-NMR spectrum (DMSO-*d*6) of compound 8

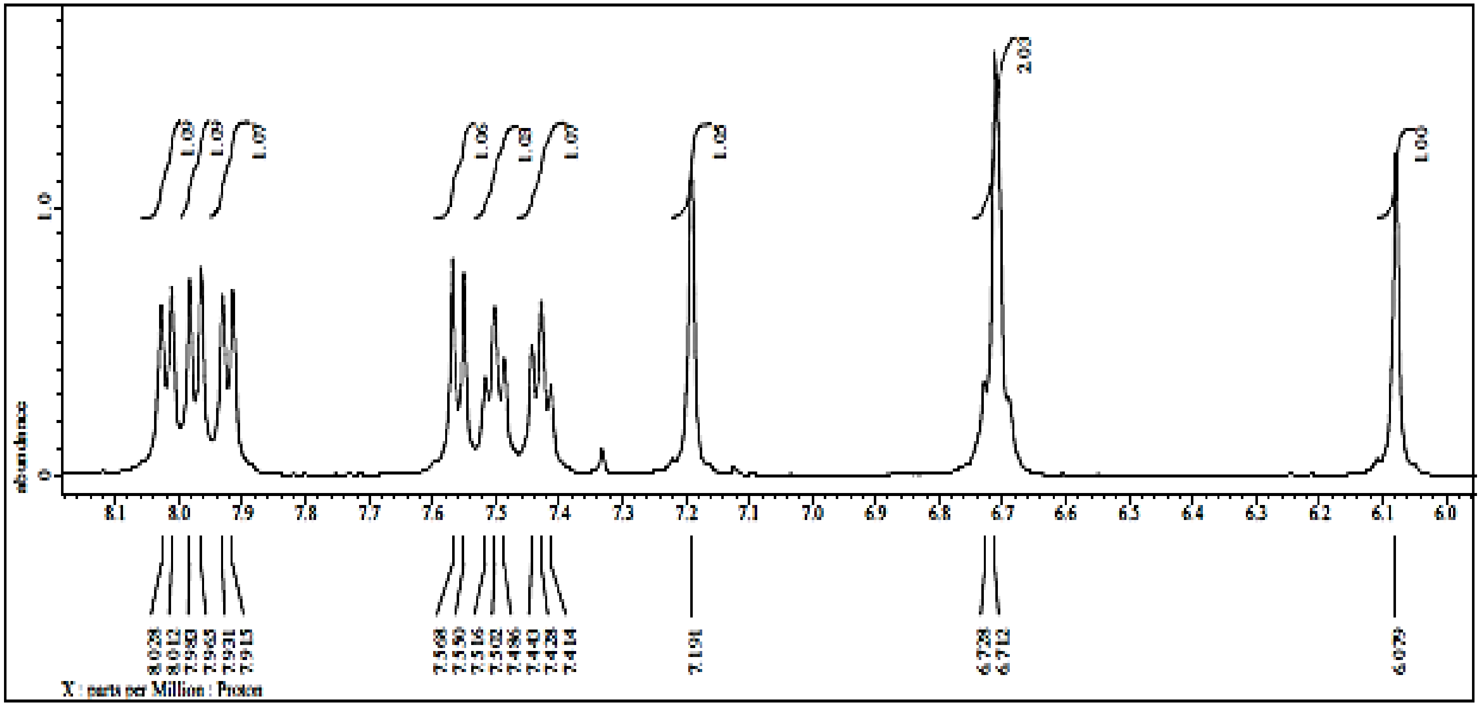

^{1}H-NMR spectrum (DMSO-*d*6) of compound 8

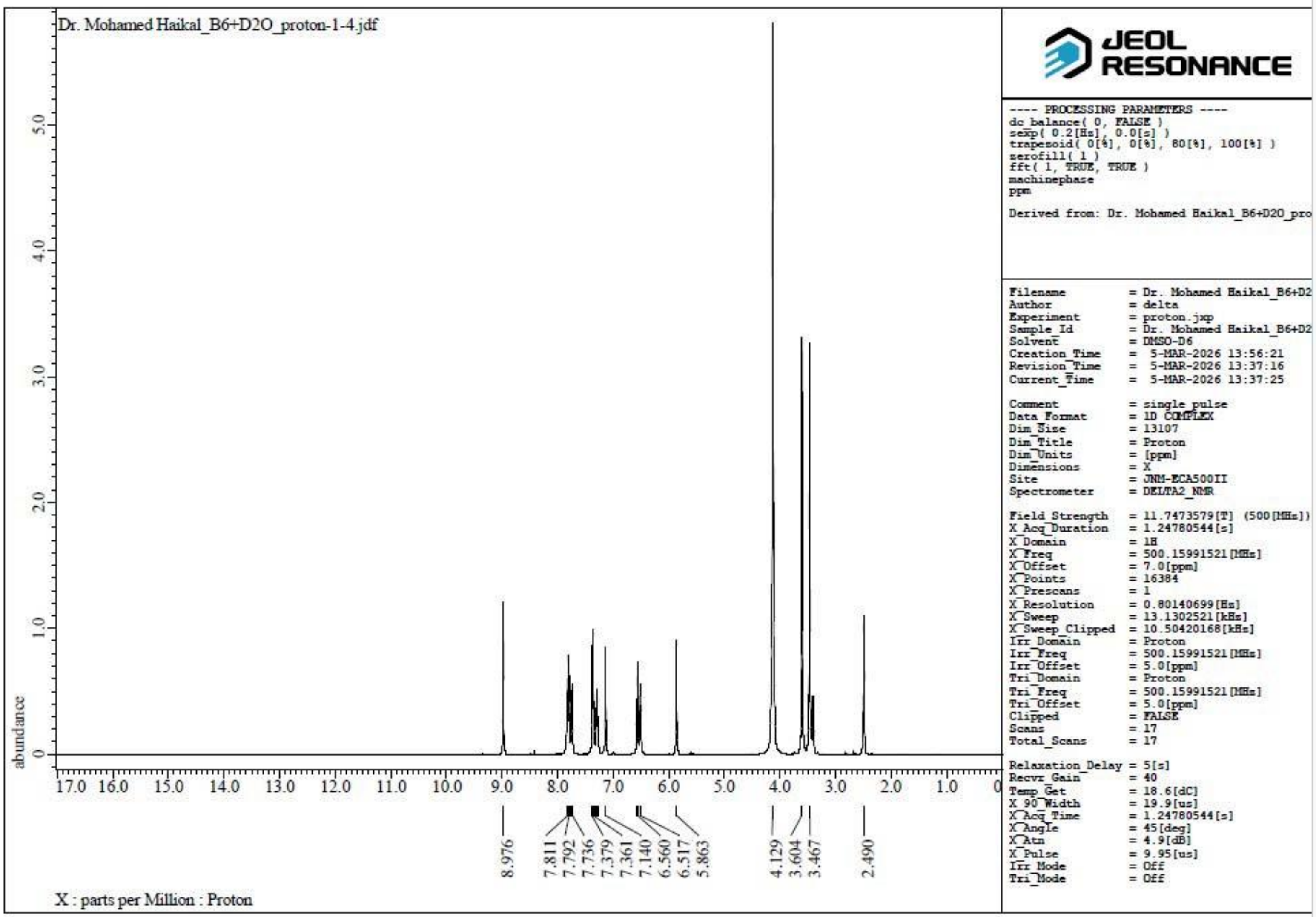

^{1}H-NMR spectrum (DMSO-*d*6+D_2O) of compound 8

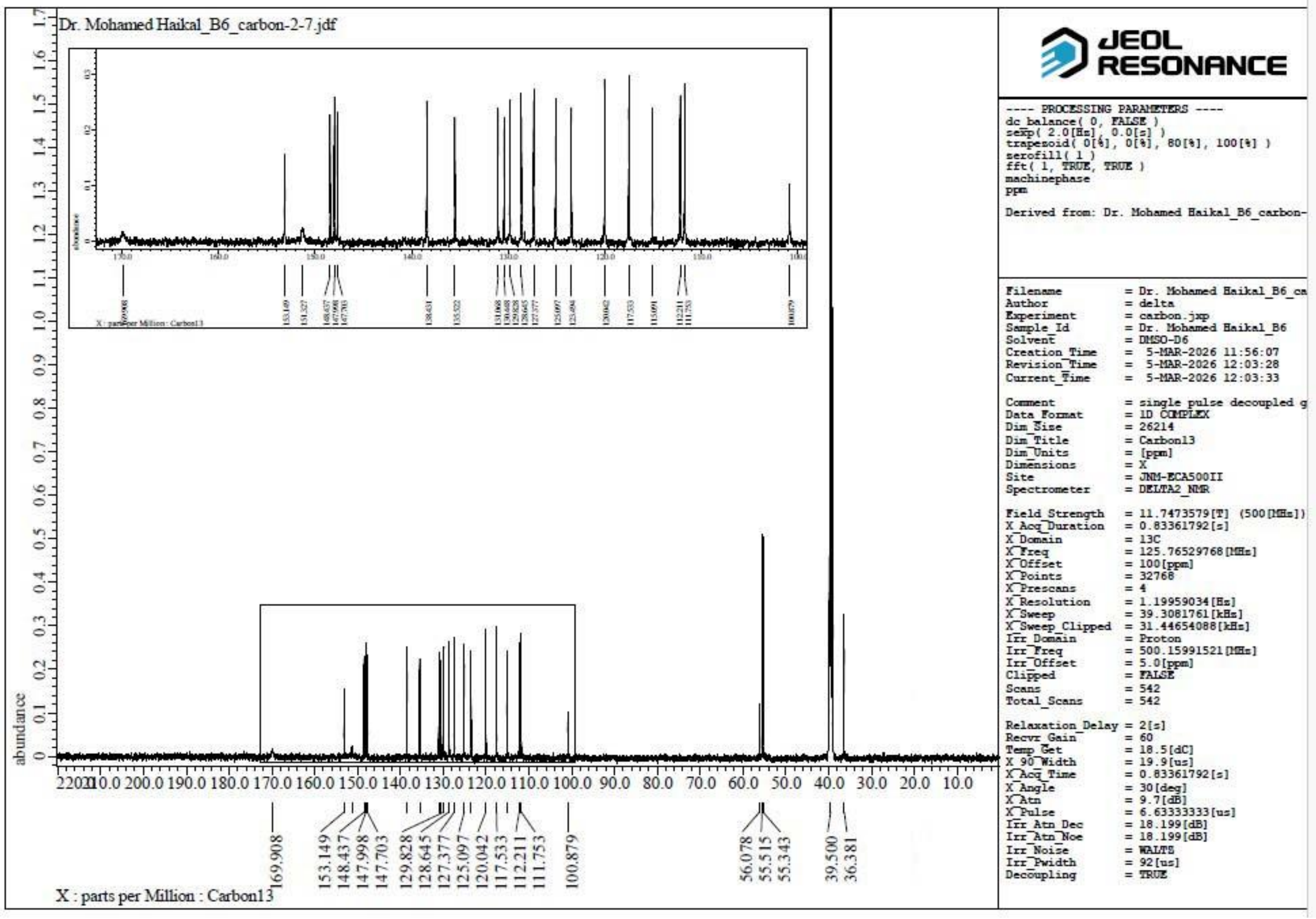

^{13}C-NMR spectrum (DMSO-d_6) of compound 8

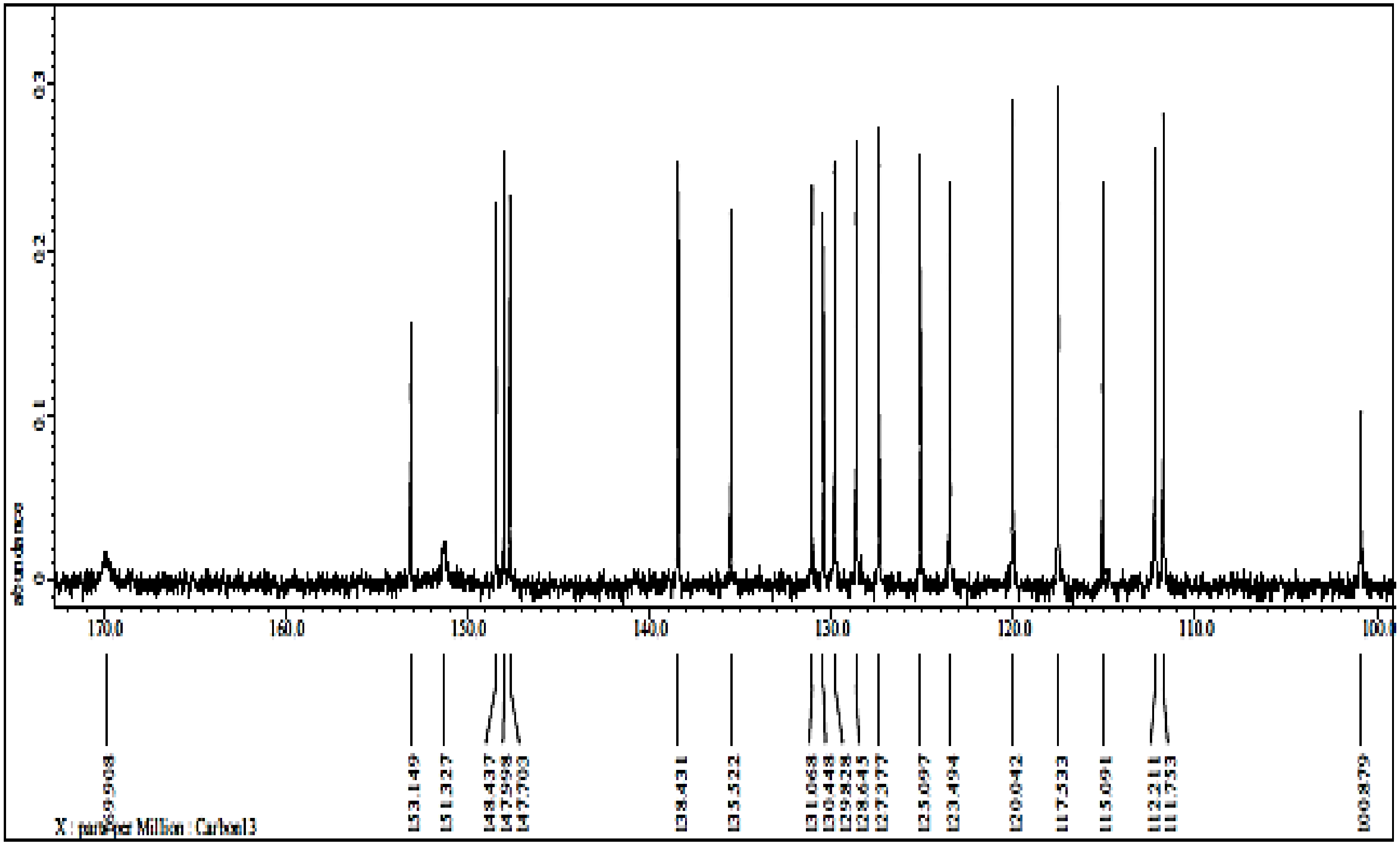

^{13}C-NMR spectrum (DMSO-d_6) of compound 8

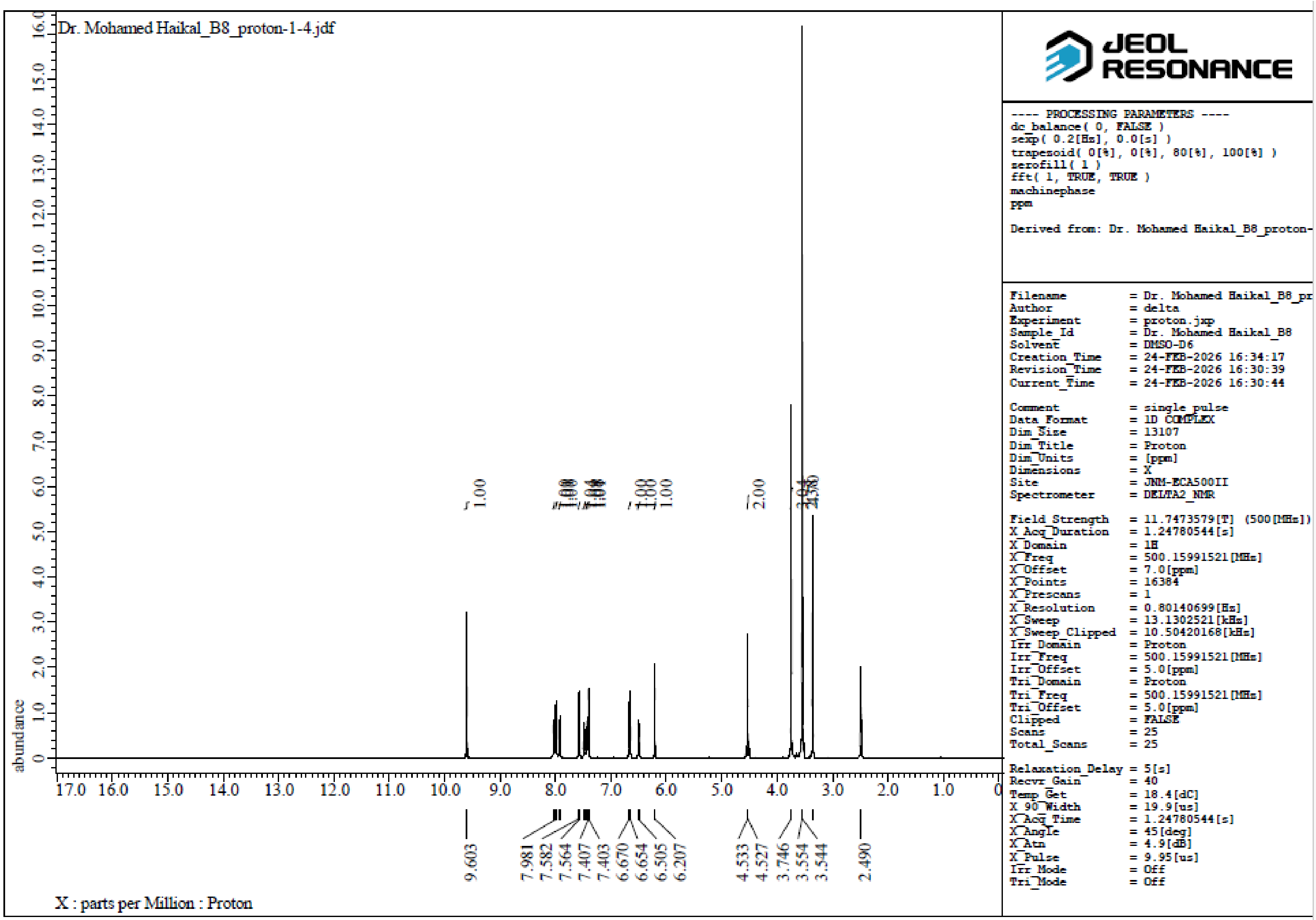

^{1}H-NMR spectrum (DMSO-*d*6) of compound 9

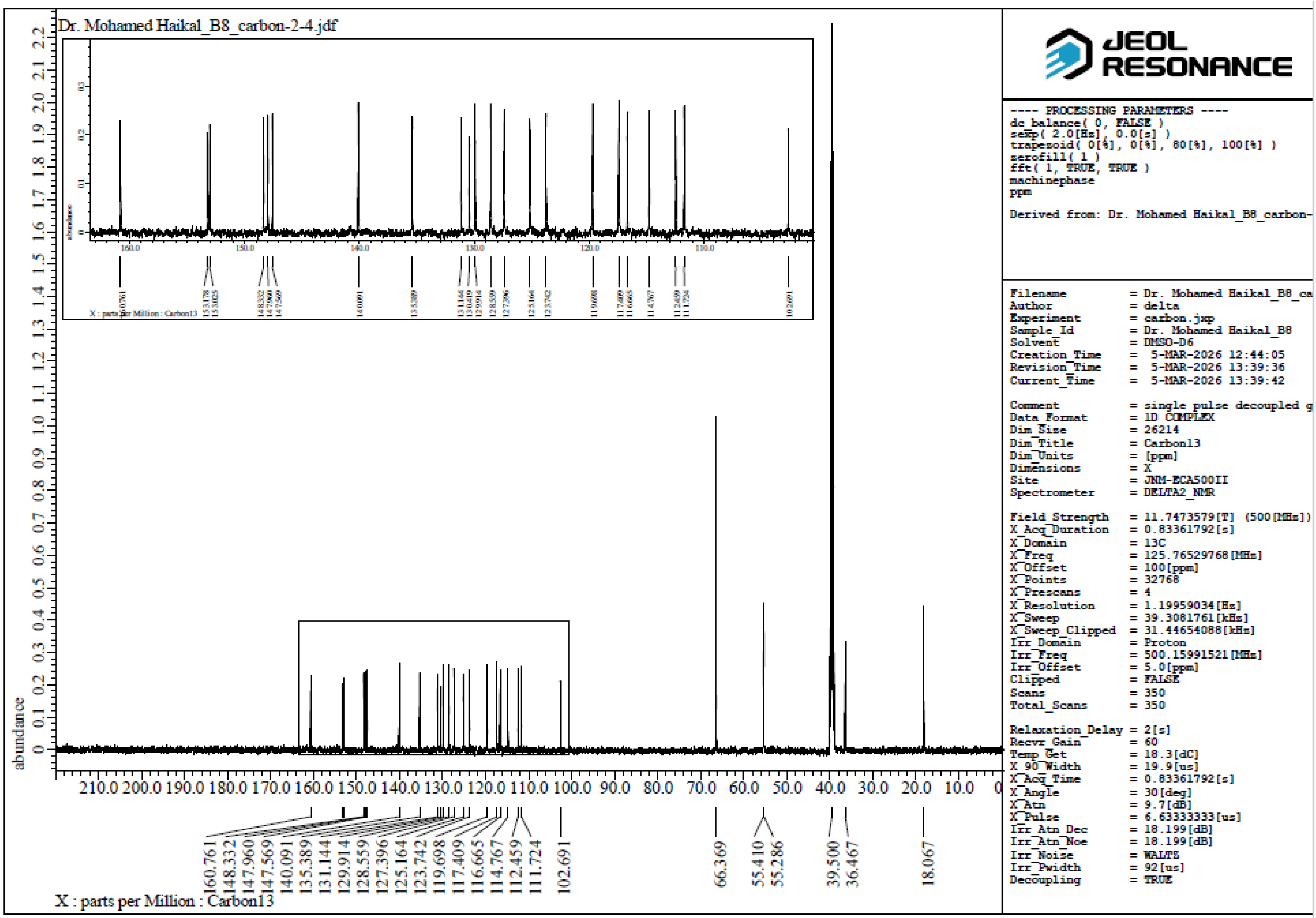

^{13}C-NMR spectrum (DMSO-d_6) of compound 9

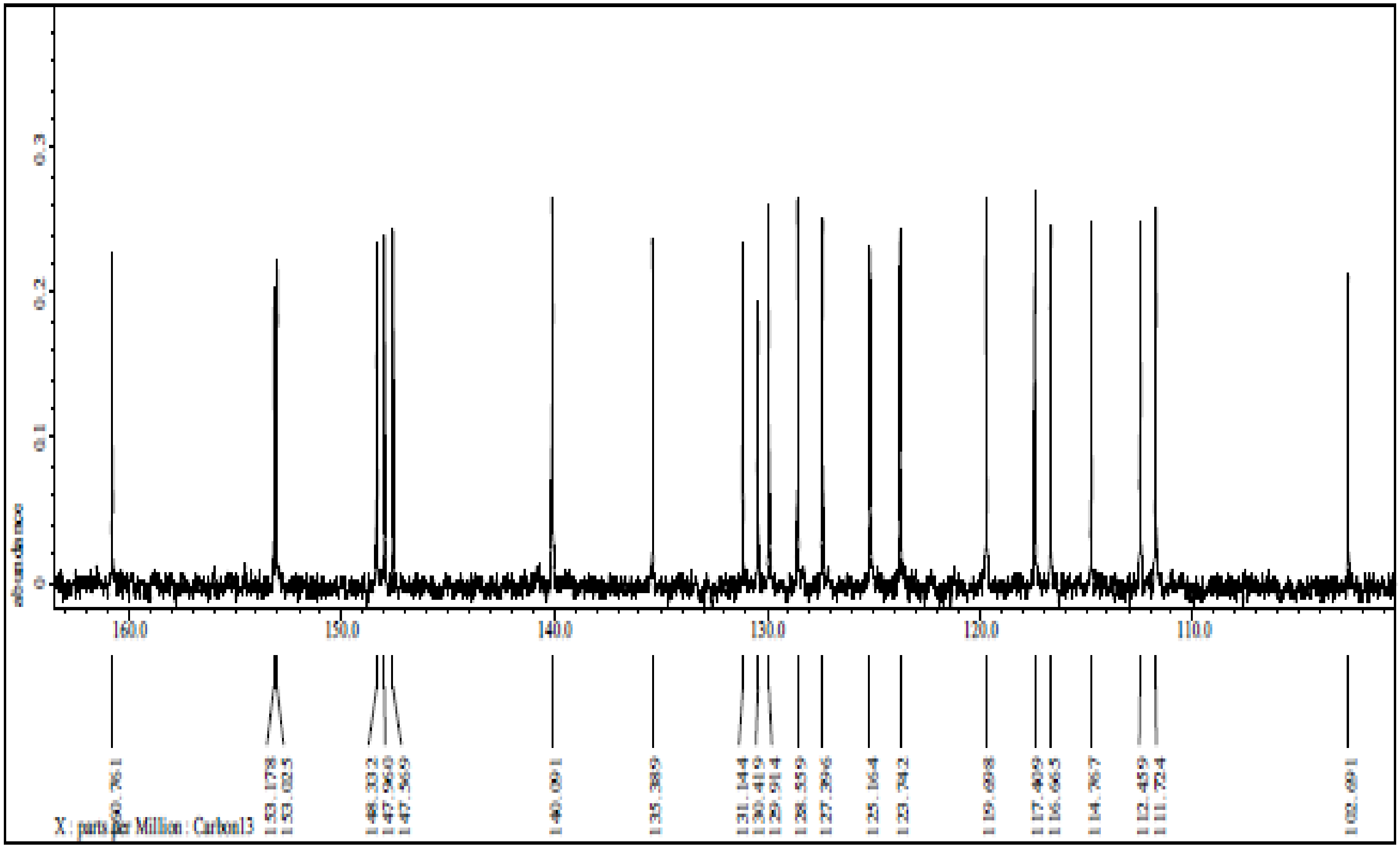

^{13}C-NMR spectrum (DMSO-d_6) of compound 9

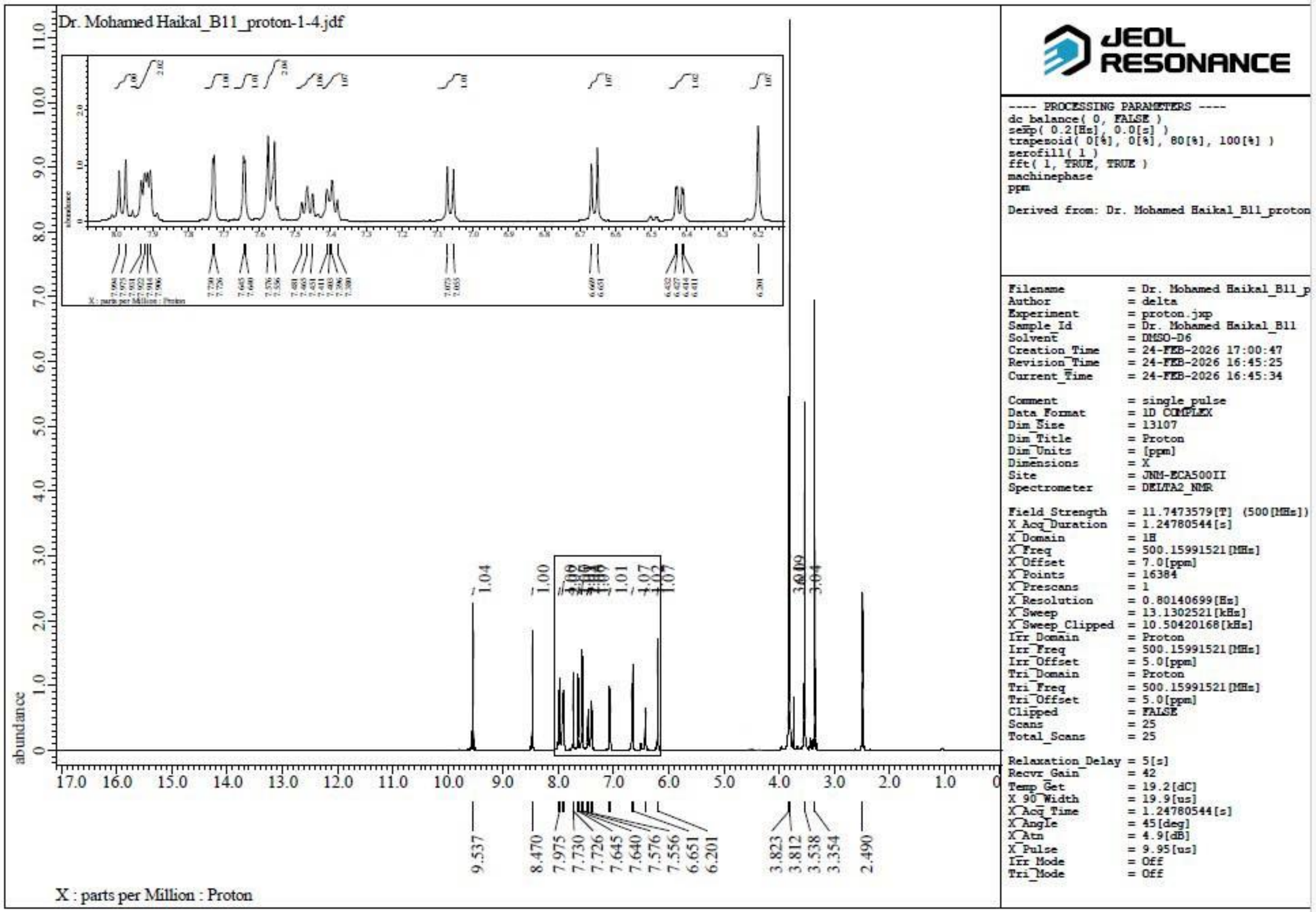

^{1}H-NMR spectrum (DMSO-*d*6) of compound 10

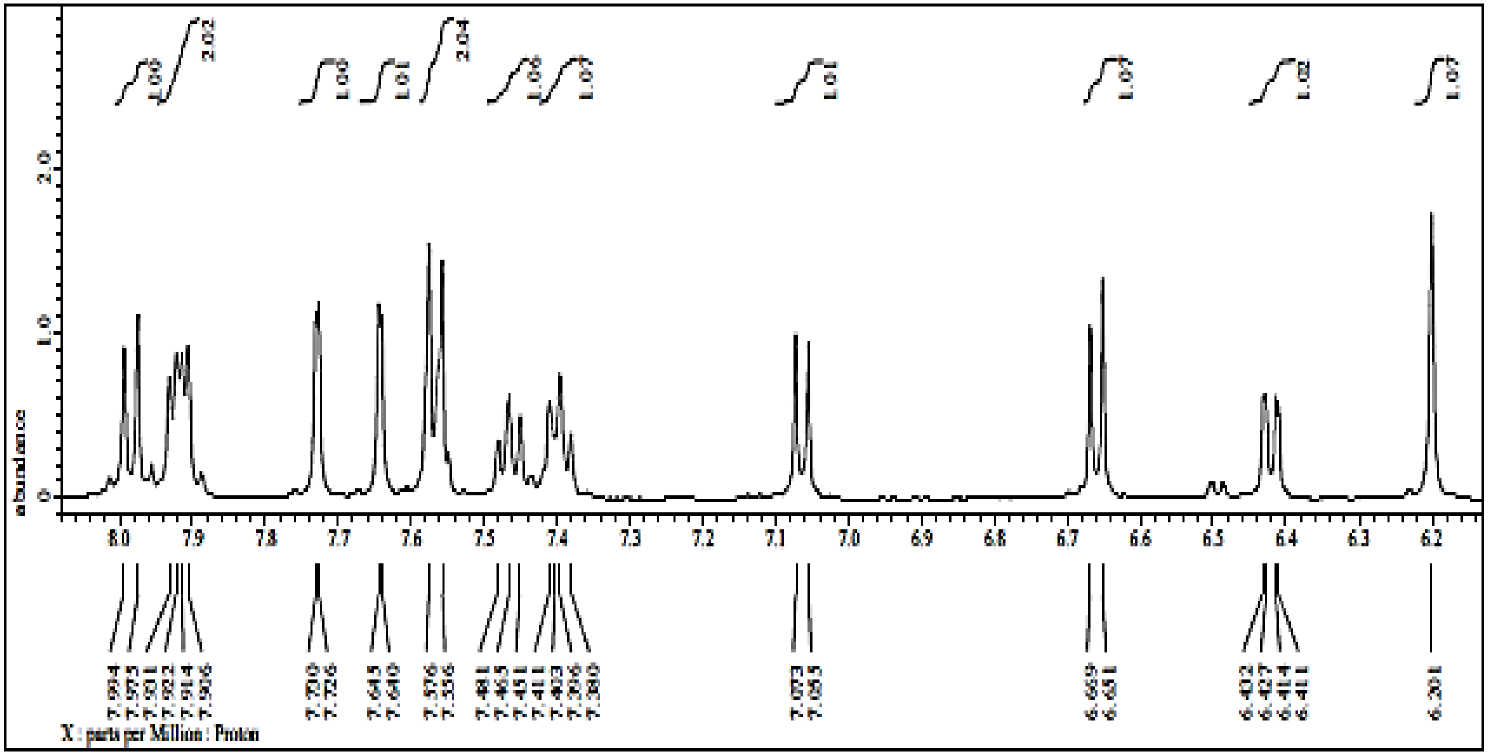

^{1}H-NMR spectrum (DMSO-*d*6) of compound 10

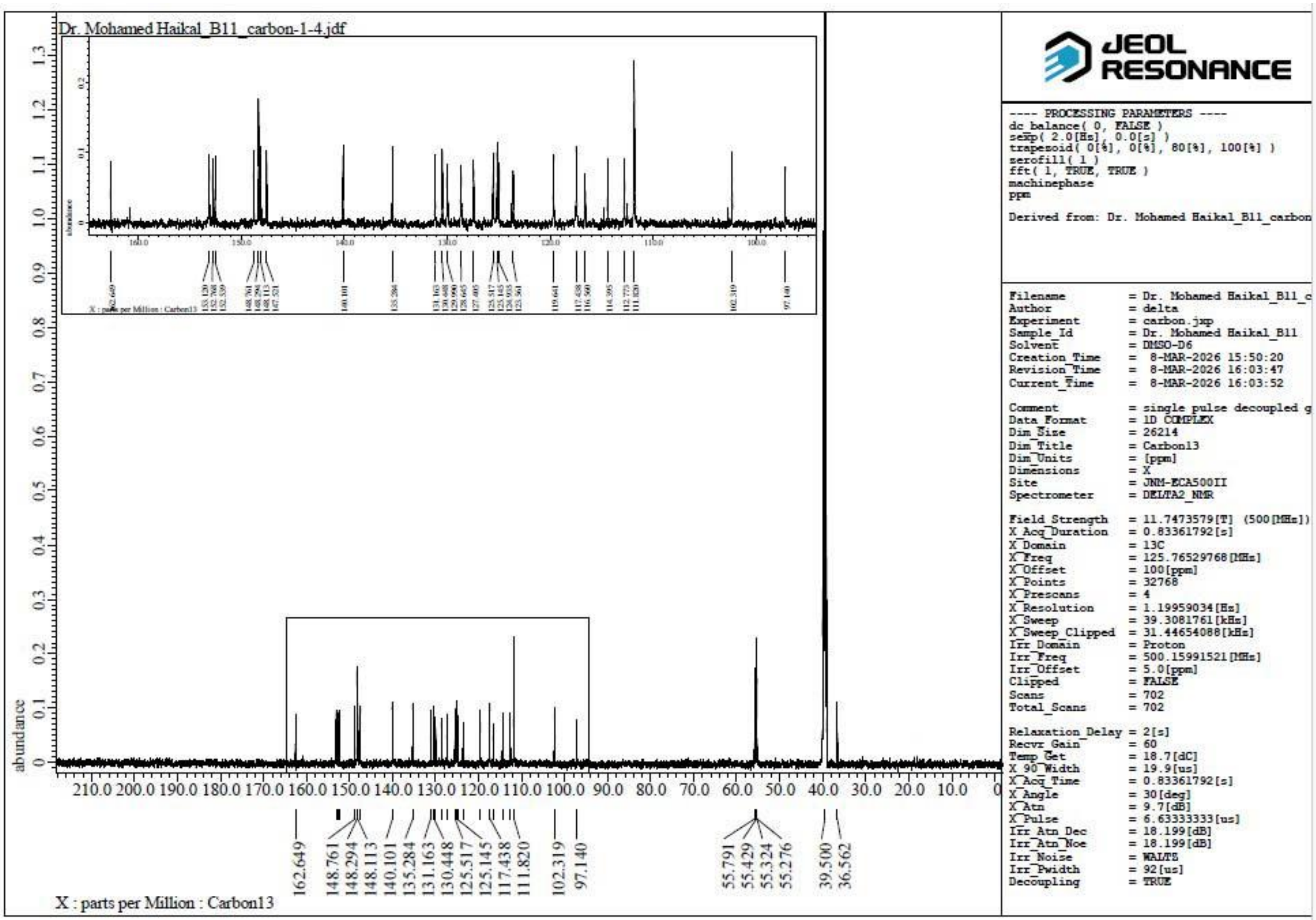

^{13}C-NMR spectrum (DMSO-d_6) of compound 10

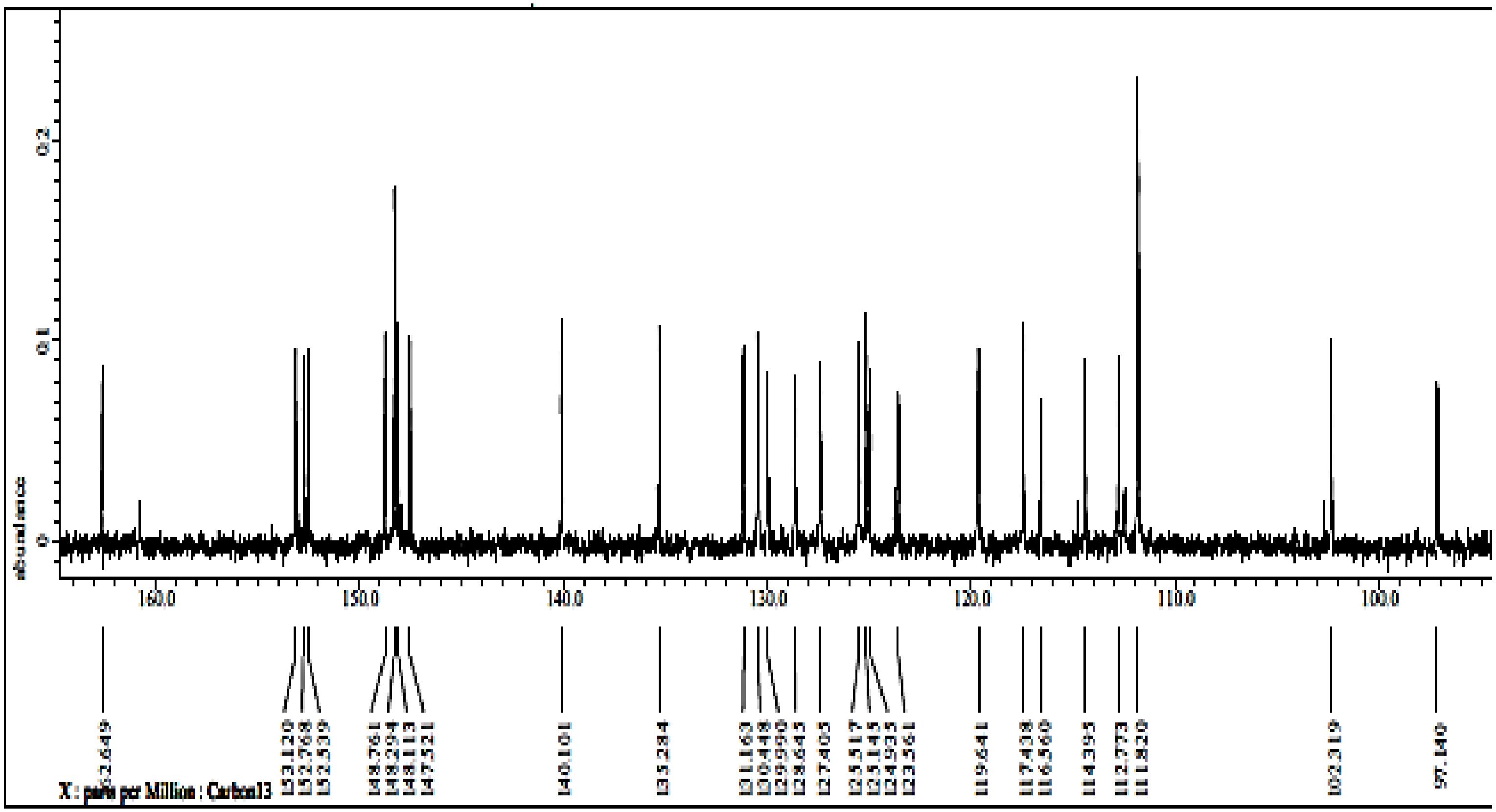

^{13}C-NMR spectrum (DMSO-d_6) of compound 10
