# Supplementary material for: New benzochromene-based compounds as potential EGFR-TK inhibitors: synthesis, anti-proliferative activity, molecular docking studies, and ADME profiles
Source: RSC Adv. 2026 Apr 30;16(25):22450–64. doi: 10.1039/d6ra02423h (PMC13130050; doi:10.1039/d6ra02423h)
Supplement: RA-016-D6RA02423H-s002 [file RA-016-D6RA02423H-s002.pdf]

Supplementary Information (SI) for RSC Advances.
This journal is © The Royal Society of Chemistry 2026

Supplementary Data File

1. SWISSADME profiling of the synthesized candidates

Cpds	Smiles
2	N=C1C(C2C3=CC(OC)=C(OC)C=C3)=C(N=CN1)OC4=C2C5=C(C=C4)C=CC=C5
3	OC1=C(C#N)C(C2=CC(OC)=C(OC)C=C2)C3=C(O1)C=CC4=C3C=CC=C4
4	CCO/C=N/C1=C(C#N)C(C2=CC(OC)=C(OC)C=C2)C3=C(O1)C=CC4=C3C=CC=C4
5	N=C1C(C2C3=CC(OC)=C(OC)C=C3)=C(N=CN1N)OC4=C2C5=C(C=C4)C=CC=C5
6	N=C1C(C2C3=CC(OC)=C(OC)C=C3)=C(N=CN1NS(C4=CC=C(C)C=C4)(=O)=O)OC5=C2C6=C(C=C5)C=CC=C6
7	N=C1C(C2C3=CC(OC)=C(OC)C=C3)=C(N=CN1C4=C(C)N(C)N(C5=CC=CC=C5)C4=O)OC6=C2C7=C(C=C6)C=CC=C7
8	COC1=C(OC)C=CC(C2C(C3=NC4=O)=C(N=CN3N4)OC5=C2C6=C(C=C5)C=CC=C6)=C1
9	COC(C=C1)=C(OC)C=C1C2C(C3=NC(CC#N)=NN3C=N4)=C4OC5=C2C6=C(C=C5)C=CC=C6
10	COC(C=C1)=C(OC)C=C1C2C(C3=NC(/C(C#N)=C/C4=CC(OC)=C(OC)C=C4)=NN3C=N5)=C5OC6=C2C7=C(C=C6)C=CC=C7

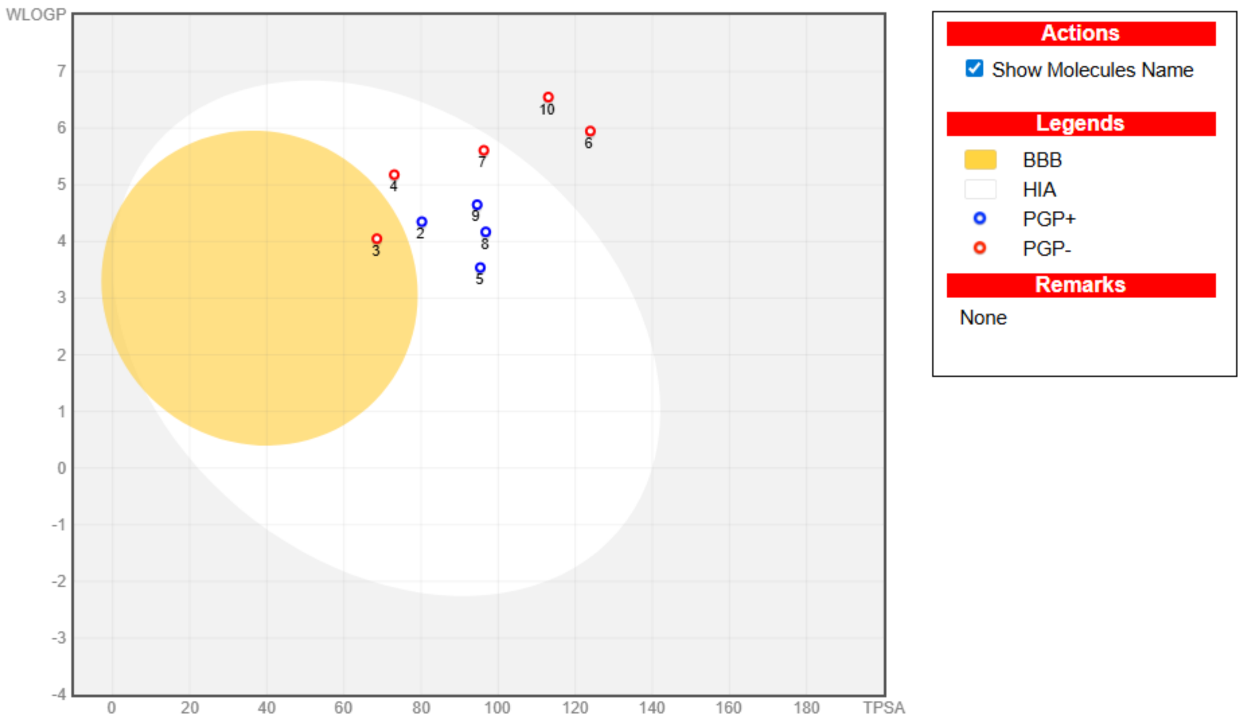
WLOGP
7
6
5
4
3
2
1
0
-1
-2
-3
-4
0
20
40
60
80
100
120
140
160
180
TPSA
10
6
7
4
9
2
8
3
5
Actions
Show Molecules Name
Legends
BBB
HIA
PGP+
PGP-
Remarks
None

2

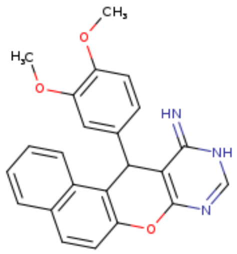

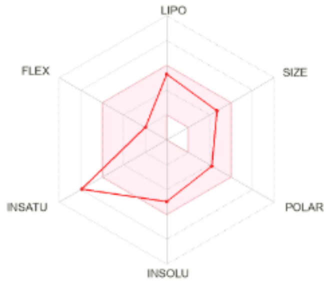

SMILES COc1ccc(cc1OC)C1c2c(nc[nH]c2=N)Oc2c1c1ccccc1cc2

Physicochemical Properties	
Formula	C23H19N3O3
Molecular weight	385.42 g/mol
Num. heavy atoms	29
Num. arom. heavy atoms	22
Fraction Csp3	0.13
Num. rotatable bonds	3
Num. H-bond acceptors	5
Num. H-bond donors	2
Molar Refractivity	109.62
TPSA	80.22 Å²

Lipophilicity	
Log $P_{o/w}$ (iLOGP)	2.83
Log $P_{o/w}$ (XLOGP3)	3.77
Log $P_{o/w}$ (WLOGP)	4.35
Log $P_{o/w}$ (MLOGP)	2.55
Log $P_{o/w}$ (SILICOS-IT)	4.90
Consensus Log $P_{o/w}$	3.68

Water Solubility	
Log *S* (ESOL)	-4.97
Solubility	4.15e-03 mg/ml ; 1.08e-05 mol/l
Class	Moderately soluble
Log *S* (Ali)	-5.15
Solubility	2.74e-03 mg/ml ; 7.11e-06 mol/l
Class	Moderately soluble
Log *S* (SILICOS-IT)	-8.38
Solubility	1.59e-06 mg/ml ; 4.12e-09 mol/l
Class	Poorly soluble

Pharmacokinetics	
GI absorption	High
BBB permeant	No
P-gp substrate	Yes
CYP1A2 inhibitor	Yes
CYP2C19 inhibitor	Yes
CYP2C9 inhibitor	Yes
CYP2D6 inhibitor	Yes
CYP3A4 inhibitor	Yes
Log K_p (skin permeation)	-5.97 cm/s

Druglikeness	
Lipinski	Yes; 0 violation
Ghose	Yes
Veber	Yes
Egan	Yes
Muegge	Yes
Bioavailability Score	0.55

Medicinal Chemistry	
PAINS	0 alert
Brenk	0 alert
Leadlikeness	No; 2 violations: MW>350, XLOGP3>3.5
Synthetic accessibility	4.02

3

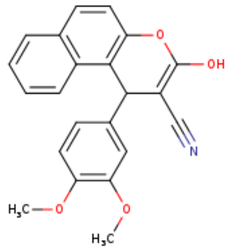

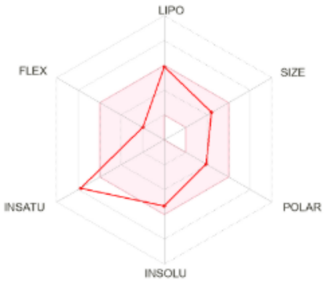

SMILES N#CC1=C(O)Oc2c(C1c1ccc(c(c1)OC)OC)c1ccccc1cc2

Physicochemical Properties	
Formula	C22H17NO4
Molecular weight	359.37 g/mol
Num. heavy atoms	27
Num. arom. heavy atoms	16
Fraction Csp3	0.14
Num. rotatable bonds	3
Num. H-bond acceptors	5
Num. H-bond donors	1
Molar Refractivity	101.22
TPSA	71.71 Å²

Lipophilicity	
Log $P_{o/w}$ (iLOGP)	2.90
Log $P_{o/w}$ (XLOGP3)	4.81
Log $P_{o/w}$ (WLOGP)	4.67
Log $P_{o/w}$ (MLOGP)	2.22
Log $P_{o/w}$ (SILICOS-IT)	4.02
Consensus Log $P_{o/w}$	3.72

Water Solubility	
Log *S* (ESOL)	-5.34
Solubility	1.65e-03 mg/ml ; 4.58e-06 mol/l
Class	Moderately soluble
Log *S* (Ali)	-6.05
Solubility	3.21e-04 mg/ml ; 8.94e-07 mol/l
Class	Poorly soluble
Log *S* (SILICOS-IT)	-6.62
Solubility	8.60e-05 mg/ml ; 2.39e-07 mol/l
Class	Poorly soluble

Pharmacokinetics	
GI absorption	High
BBB permeant	No
P-gp substrate	No
CYP1A2 inhibitor	Yes
CYP2C19 inhibitor	Yes
CYP2C9 inhibitor	Yes
CYP2D6 inhibitor	No
CYP3A4 inhibitor	Yes
Log K_p (skin permeation)	-5.08 cm/s

Druglikeness	
Lipinski	Yes; 0 violation
Ghose	Yes
Veber	Yes
Egan	Yes
Muegge	Yes
Bioavailability Score	0.55

Medicinal Chemistry	
PAINS	0 alert
Brenk	0 alert
Leadlikeness	No; 2 violations: MW>350, XLOGP3>3.5
Synthetic accessibility	3.97

4

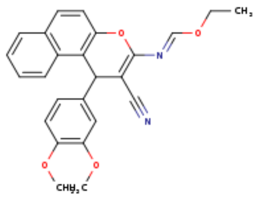

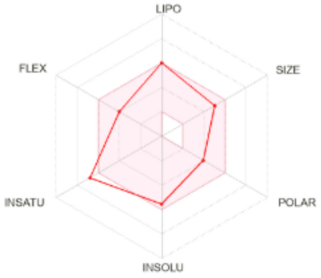

SMILES CCO/C=N/C1=C(C#N)C(c2c(O1)ccc1c2cccc1)c1ccc(c(c1)OC)OC

Physicochemical Properties	
Formula	C25H22N2O4
Molecular weight	414.45 g/mol
Num. heavy atoms	31
Num. arom. heavy atoms	16
Fraction Csp3	0.20
Num. rotatable bonds	6
Num. H-bond acceptors	6
Num. H-bond donors	0
Molar Refractivity	118.84
TPSA	73.07 Å²

Lipophilicity	
Log $P_{o/w}$ (iLOGP)	4.07
Log $P_{o/w}$ (XLOGP3)	5.03
Log $P_{o/w}$ (WLOGP)	5.18
Log $P_{o/w}$ (MLOGP)	2.78
Log $P_{o/w}$ (SILICOS-IT)	5.36
Consensus Log $P_{o/w}$	4.48

Water Solubility	
Log *S* (ESOL)	-5.56
Solubility	1.13e-03 mg/ml ; 2.73e-06 mol/l
Class	Moderately soluble
Log *S* (Ali)	-6.31
Solubility	2.05e-04 mg/ml ; 4.95e-07 mol/l
Class	Poorly soluble
Log *S* (SILICOS-IT)	-7.78
Solubility	6.93e-06 mg/ml ; 1.67e-08 mol/l
Class	Poorly soluble

Pharmacokinetics	
GI absorption	High
BBB permeant	No
P-gp substrate	No
CYP1A2 inhibitor	No
CYP2C19 inhibitor	Yes
CYP2C9 inhibitor	Yes
CYP2D6 inhibitor	No
CYP3A4 inhibitor	Yes
Log K_p (skin permeation)	-5.26 cm/s

Druglikeness	
Lipinski	Yes; 0 violation
Ghose	Yes
Veber	Yes
Egan	Yes
Muegge	No; 1 violation: XLOGP3>5
Bioavailability Score	0.55

Medicinal Chemistry	
PAINS	0 alert
Brenk	2 alerts: imine_1, imine_2
Leadlikeness	No; 2 violations: MW>350, XLOGP3>3.5
Synthetic accessibility	4.54

5

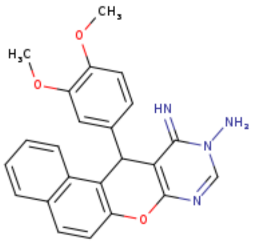

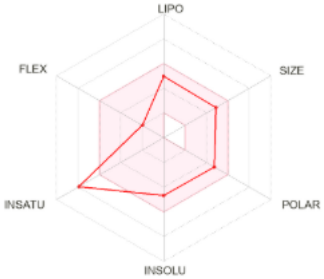

SMILES COc1ccc(cc1OC)C1c2c(ncn(c2=N)N)Oc2c1c1ccccc1cc2

Physicochemical Properties	
Formula	C23H20N4O3
Molecular weight	400.43 g/mol
Num. heavy atoms	30
Num. arom. heavy atoms	22
Fraction Csp3	0.13
Num. rotatable bonds	3
Num. H-bond acceptors	5
Num. H-bond donors	2
Molar Refractivity	113.40
TPSA	95.38 Å²

Lipophilicity	
Log $P_{o/w}$ (iLOGP)	2.75
Log $P_{o/w}$ (XLOGP3)	3.21
Log $P_{o/w}$ (WLOGP)	3.54
Log $P_{o/w}$ (MLOGP)	2.84
Log $P_{o/w}$ (SILICOS-IT)	3.13
Consensus Log $P_{o/w}$	3.09

Water Solubility	
Log *S* (ESOL)	-4.69
Solubility	8.18e-03 mg/ml ; 2.04e-05 mol/l
Class	Moderately soluble
Log *S* (Ali)	-4.89
Solubility	5.22e-03 mg/ml ; 1.30e-05 mol/l
Class	Moderately soluble
Log *S* (SILICOS-IT)	-7.19
Solubility	2.59e-05 mg/ml ; 6.47e-08 mol/l
Class	Poorly soluble

Pharmacokinetics	
GI absorption	High
BBB permeant	No
P-gp substrate	Yes
CYP1A2 inhibitor	No
CYP2C19 inhibitor	Yes
CYP2C9 inhibitor	Yes
CYP2D6 inhibitor	Yes
CYP3A4 inhibitor	No
Log K_p (skin permeation)	-6.46 cm/s

Druglikeness	
Lipinski	Yes; 0 violation
Ghose	Yes
Veber	Yes
Egan	Yes
Muegge	Yes
Bioavailability Score	0.55

Medicinal Chemistry	
PAINS	0 alert
Brenk	0 alert
Leadlikeness	No; 1 violation: MW>350
Synthetic accessibility	4.23

6

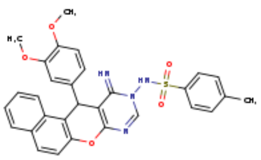

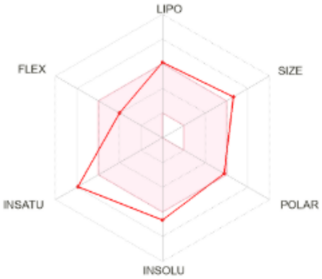

SMILES COc1ccc(cc1OC)C1c2c(ncn(c2=N)NS(=O)(=O)c2ccc(cc2)C)Oc2c1c1ccccc1cc2

Physicochemical Properties	
Formula	C30H26N4O5S
Molecular weight	554.62 g/mol
Num. heavy atoms	40
Num. arom. heavy atoms	28
Fraction Csp3	0.13
Num. rotatable bonds	6
Num. H-bond acceptors	7
Num. H-bond donors	2
Molar Refractivity	151.07
TPSA	123.91 Å²

Lipophilicity	
Log $P_{o/w}$ (iLOGP)	3.49
Log $P_{o/w}$ (XLOGP3)	5.17
Log $P_{o/w}$ (WLOGP)	5.95
Log $P_{o/w}$ (MLOGP)	3.42
Log $P_{o/w}$ (SILICOS-IT)	3.99
Consensus Log $P_{o/w}$	4.40

Water Solubility	
Log *S* (ESOL)	-6.66
Solubility	1.22e-04 mg/ml ; 2.20e-07 mol/l
Class	Poorly soluble
Log *S* (Ali)	-7.52
Solubility	1.68e-05 mg/ml ; 3.03e-08 mol/l
Class	Poorly soluble
Log *S* (SILICOS-IT)	-10.26
Solubility	3.02e-08 mg/ml ; 5.45e-11 mol/l
Class	Insoluble

Pharmacokinetics	
GI absorption	Low
BBB permeant	No
P-gp substrate	No
CYP1A2 inhibitor	No
CYP2C19 inhibitor	No
CYP2C9 inhibitor	Yes
CYP2D6 inhibitor	No
CYP3A4 inhibitor	No
Log K_p (skin permeation)	-6.01 cm/s

Druglikeness	
Lipinski	Yes; 1 violation: MW>500
Ghose	No; 3 violations: MW>480, WLOGP>5.6, MR>130
Veber	Yes
Egan	No; 1 violation: WLOGP>5.88
Muegge	No; 1 violation: XLOGP3>5
Bioavailability Score	0.55

Medicinal Chemistry	
PAINS	0 alert
Brenk	0 alert
Leadlikeness	No; 2 violations: MW>350, XLOGP3>3.5
Synthetic accessibility	4.91

7

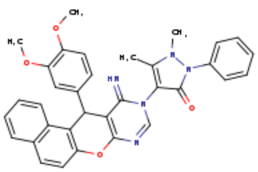

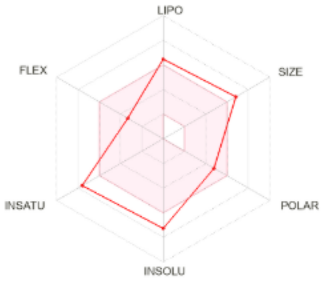

SMILES COc1cc(ccc1OC)C1c2c(ccc3c2cccc3)Oc2c1c(=N)n(cn2)c1c(C)n(n(c1=O)c1ccccc1)C

Physicochemical Properties	
Formula	C34H29N5O4
Molecular weight	571.63 g/mol
Num. heavy atoms	43
Num. arom. heavy atoms	33
Fraction Csp3	0.15
Num. rotatable bonds	5
Num. H-bond acceptors	6
Num. H-bond donors	1
Molar Refractivity	164.41
TPSA	96.29 Å²

Lipophilicity	
Log $P_{o/w}$ (iLOGP)	4.46
Log $P_{o/w}$ (XLOGP3)	5.78
Log $P_{o/w}$ (WLOGP)	5.61
Log $P_{o/w}$ (MLOGP)	4.07
Log $P_{o/w}$ (SILICOS-IT)	4.99
Consensus Log $P_{o/w}$	4.98

Water Solubility	
Log *S* (ESOL)	-7.26
Solubility	3.12e-05 mg/ml ; 5.45e-08 mol/l
Class	Poorly soluble
Log *S* (Ali)	-7.57
Solubility	1.53e-05 mg/ml ; 2.68e-08 mol/l
Class	Poorly soluble
Log *S* (SILICOS-IT)	-10.26
Solubility	3.11e-08 mg/ml ; 5.45e-11 mol/l
Class	Insoluble

Pharmacokinetics	
GI absorption	High
BBB permeant	No
P-gp substrate	No
CYP1A2 inhibitor	No
CYP2C19 inhibitor	No
CYP2C9 inhibitor	Yes
CYP2D6 inhibitor	No
CYP3A4 inhibitor	No
Log K_p (skin permeation)	-5.68 cm/s

Druglikeness	
Lipinski	Yes; 1 violation: MW>500
Ghose	No; 4 violations: MW>480, WLOGP>5.6, MR>130, #atoms>70
Veber	Yes
Egan	Yes
Muegge	No; 1 violation: XLOGP3>5
Bioavailability Score	0.55

Medicinal Chemistry	
PAINS	0 alert
Brenk	0 alert
Leadlikeness	No; 2 violations: MW>350, XLOGP3>3.5
Synthetic accessibility	5.08

8

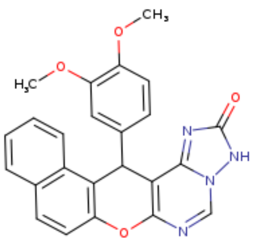

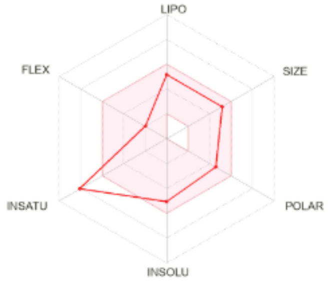

SMILES COc1ccc(cc1OC)C1c2c(ccc3c2cccc3)Oc2c1c1nc(=O)[nH]n1cn2

Physicochemical Properties	
Formula	C24H18N4O4
Molecular weight	426.42 g/mol
Num. heavy atoms	32
Num. arom. heavy atoms	25
Fraction Csp3	0.12
Num. rotatable bonds	3
Num. H-bond acceptors	6
Num. H-bond donors	1
Molar Refractivity	118.11
TPSA	90.74 Å²

Lipophilicity	
Log $P_{o/w}$ (iLOGP)	2.76
Log $P_{o/w}$ (XLOGP3)	3.52
Log $P_{o/w}$ (WLOGP)	3.87
Log $P_{o/w}$ (MLOGP)	2.79
Log $P_{o/w}$ (SILICOS-IT)	3.64
Consensus Log $P_{o/w}$	3.32

Water Solubility	
Log *S* (ESOL)	-5.08
Solubility	3.53e-03 mg/ml ; 8.29e-06 mol/l
Class	Moderately soluble
Log *S* (Ali)	-5.11
Solubility	3.31e-03 mg/ml ; 7.77e-06 mol/l
Class	Moderately soluble
Log *S* (SILICOS-IT)	-7.89
Solubility	5.46e-06 mg/ml ; 1.28e-08 mol/l
Class	Poorly soluble

Pharmacokinetics	
GI absorption	High
BBB permeant	No
P-gp substrate	Yes
CYP1A2 inhibitor	No
CYP2C19 inhibitor	No
CYP2C9 inhibitor	Yes
CYP2D6 inhibitor	Yes
CYP3A4 inhibitor	No
Log K_p (skin permeation)	-6.40 cm/s

Druglikeness	
Lipinski	Yes; 0 violation
Ghose	Yes
Veber	Yes
Egan	Yes
Muegge	Yes
Bioavailability Score	0.55

Medicinal Chemistry	
PAINS	0 alert
Brenk	0 alert
Leadlikeness	No; 2 violations: MW>350, XLOGP3>3.5
Synthetic accessibility	3.97

9

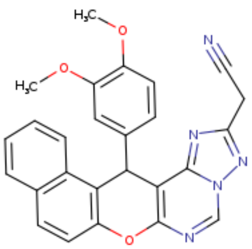

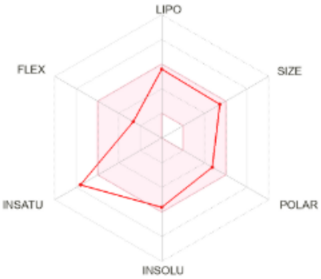

SMILES N#CCc1nn2c(n1)c1c(nc2)Oc2c(C1c1ccc(c(c1)OC)OC)c1ccccc1cc2

Physicochemical Properties	
Formula	C26H19N5O3
Molecular weight	449.46 g/mol
Num. heavy atoms	34
Num. arom. heavy atoms	25
Fraction Csp3	0.15
Num. rotatable bonds	4
Num. H-bond acceptors	7
Num. H-bond donors	0
Molar Refractivity	124.81
TPSA	94.56 Å²

Lipophilicity	
Log $P_{o/w}$ (iLOGP)	3.28
Log $P_{o/w}$ (XLOGP3)	4.30
Log $P_{o/w}$ (WLOGP)	4.65
Log $P_{o/w}$ (MLOGP)	2.80
Log $P_{o/w}$ (SILICOS-IT)	3.93
Consensus Log $P_{o/w}$	3.79

Water Solubility	
Log *S* (ESOL)	-5.62
Solubility	1.09e-03 mg/ml ; 2.42e-06 mol/l
Class	Moderately soluble
Log *S* (Ali)	-6.00
Solubility	4.50e-04 mg/ml ; 1.00e-06 mol/l
Class	Moderately soluble
Log *S* (SILICOS-IT)	-8.46
Solubility	1.58e-06 mg/ml ; 3.51e-09 mol/l
Class	Poorly soluble

Pharmacokinetics	
GI absorption	High
BBB permeant	No
P-gp substrate	Yes
CYP1A2 inhibitor	Yes
CYP2C19 inhibitor	No
CYP2C9 inhibitor	Yes
CYP2D6 inhibitor	No
CYP3A4 inhibitor	Yes
Log K_p (skin permeation)	-5.99 cm/s

Druglikeness	
Lipinski	Yes; 0 violation
Ghose	Yes
Veber	Yes
Egan	Yes
Muegge	Yes
Bioavailability Score	0.55

Medicinal Chemistry	
PAINS	0 alert
Brenk	0 alert
Leadlikeness	No; 2 violations: MW>350, XLOGP3>3.5
Synthetic accessibility	4.15

10

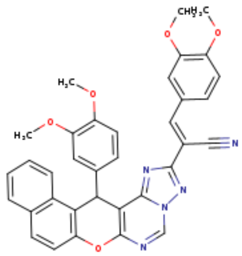

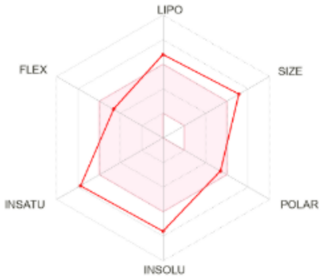

SMILES N#C/C(=C\c1ccc(c(c1)OC)OC)/c1nc2n(n1)cnc1c2C(c2ccc(c(c2)OC)OC)c2c(O1)ccc1c2cccc1

Physicochemical Properties	
Formula	C35H27N5O5
Molecular weight	597.62 g/mol
Num. heavy atoms	45
Num. arom. heavy atoms	31
Fraction Csp3	0.14
Num. rotatable bonds	7
Num. H-bond acceptors	9
Num. H-bond donors	0
Molar Refractivity	168.20
TPSA	113.02 Å²

Lipophilicity	
Log $P_{o/w}$ (iLOGP)	4.65
Log $P_{o/w}$ (XLOGP3)	6.36
Log $P_{o/w}$ (WLOGP)	6.55
Log $P_{o/w}$ (MLOGP)	3.24
Log $P_{o/w}$ (SILICOS-IT)	5.57
Consensus Log $P_{o/w}$	5.27

Water Solubility	
Log *S* (ESOL)	-7.60
Solubility	1.50e-05 mg/ml ; 2.51e-08 mol/l
Class	Poorly soluble
Log *S* (Ali)	-8.52
Solubility	1.79e-06 mg/ml ; 2.99e-09 mol/l
Class	Poorly soluble
Log *S* (SILICOS-IT)	-10.74
Solubility	1.10e-08 mg/ml ; 1.83e-11 mol/l
Class	Insoluble

Pharmacokinetics	
GI absorption	Low
BBB permeant	No
P-gp substrate	No
CYP1A2 inhibitor	No
CYP2C19 inhibitor	No
CYP2C9 inhibitor	Yes
CYP2D6 inhibitor	No
CYP3A4 inhibitor	Yes
Log K_p (skin permeation)	-5.43 cm/s

Druglikeness	
Lipinski	Yes; 1 violation: MW>500
Ghose	No; 4 violations: MW>480, WLOGP>5.6, MR>130, #atoms>70
Veber	Yes
Egan	No; 1 violation: WLOGP>5.88
Muegge	No; 1 violation: XLOGP3>5
Bioavailability Score	0.55

Medicinal Chemistry	
PAINS	0 alert
Brenk	1 alert: conjugated_nitrile_group
Leadlikeness	No; 2 violations: MW>350, XLOGP3>3.5
Synthetic accessibility	5.13

Detailed 2Dand 3D interaction diagrams of the synthesized compounds derivatives docked to EGFR tyrosine kinase (PDB ID: 4HJO). Comparisons include binding interactions of each derivative alongside the reference inhibitor doxorubicin and the co-crystallized ligand (AQ4). Notably, many of the derivatives exhibit interaction profiles that closely resemble those of the co-crystallized ligand, supporting the reliability and accuracy of the docking protocol. The diagrams highlight hydrogen bonding, π-interactions, ionic contacts, and other key molecular interactions within the active site environments.

<table>
<tr>
<td>2
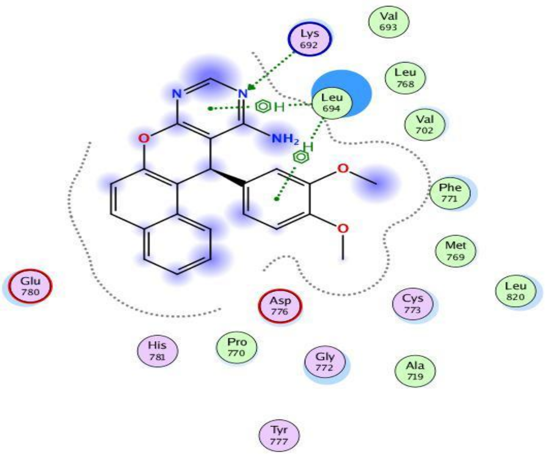
</td>
<td>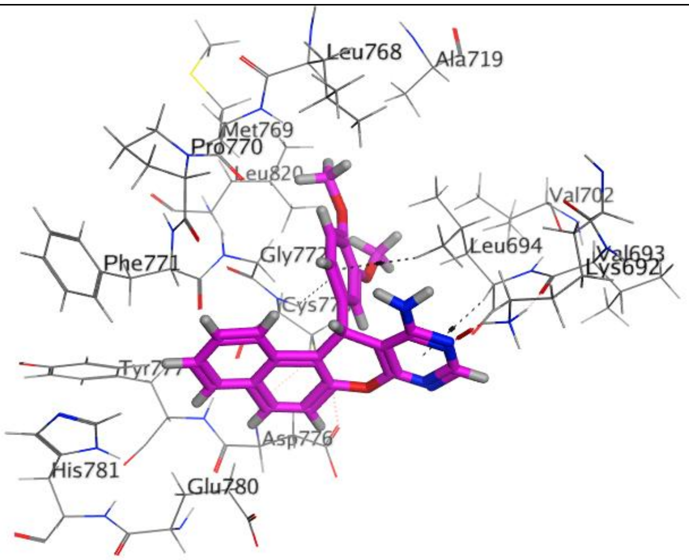
</td>
</tr>
<tr>
<td>3
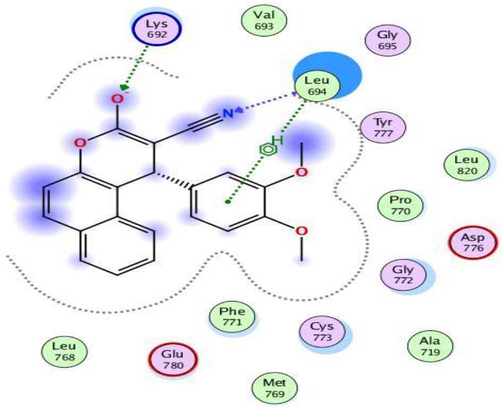
</td>
<td>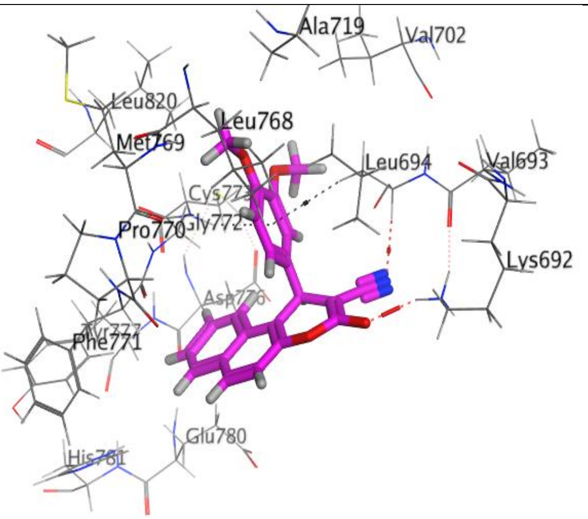
</td>
</tr>
<tr>
<td>4</td>
<td></td>
</tr>
</table>

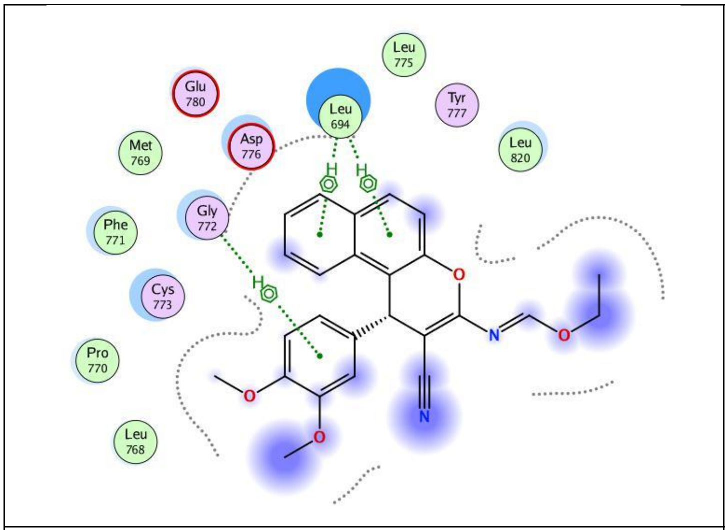

Leu 775
Glu 780
Leu 694
Tyr 777
Asp 776
Leu 820
Met 769
Gly 772
Phe 771
Cys 773
Pro 770
Leu 768

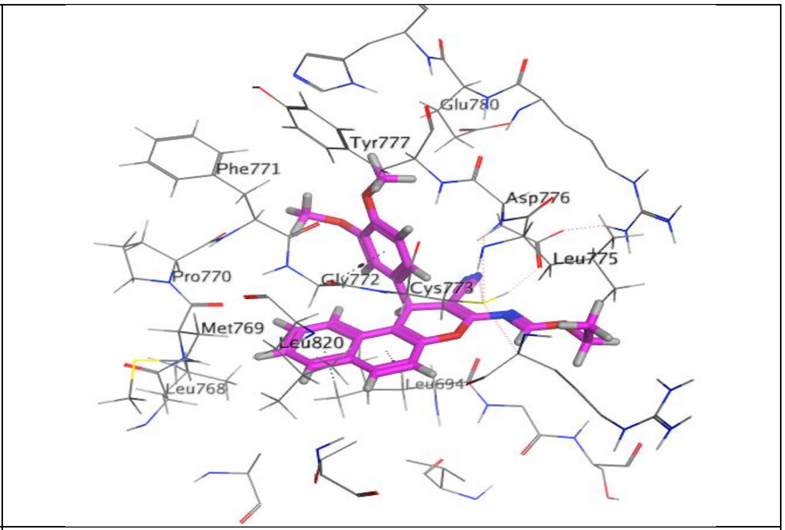

Glu780
Tyr777
Phe771
Asp776
Leu775
Gly772
Cys773
Pro770
Met769
Leu820
Leu768
Leu694

5

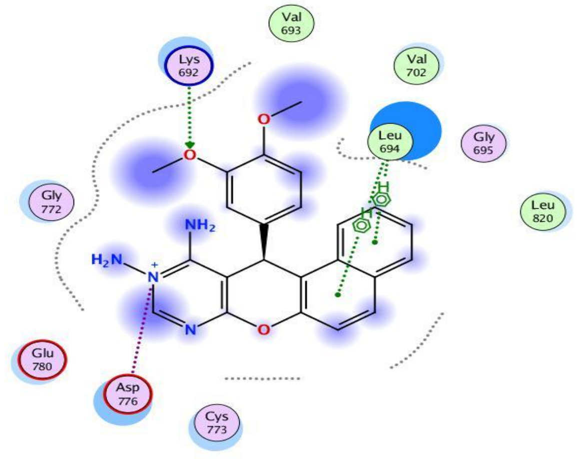

Val 693
Lys 692
Val 702
Leu 694
Gly 695
Gly 772
Leu 820
Glu 780
Asp 776
Cys 773

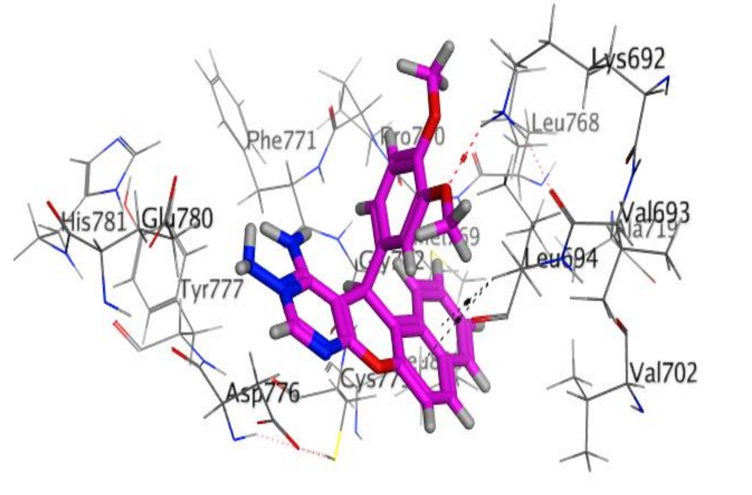

Lys692
Phe771
Leu768
His781
Glu780
Val693
Leu694
Tyr777
Asp776
Val702

6

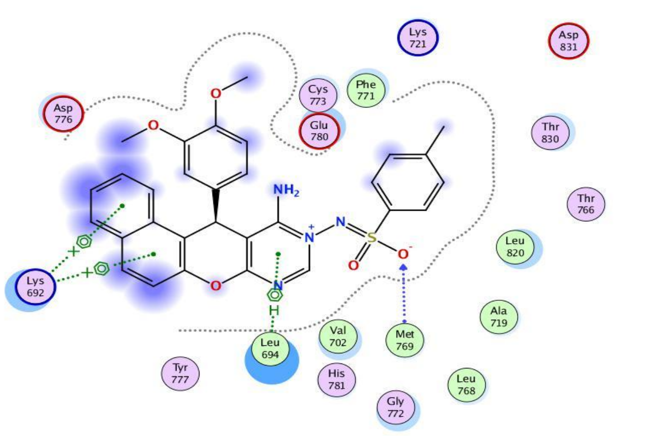

Lys 721
Asp 831
Asp 776
Cys 773
Phe 771
Glu 780
Thr 830
Thr 766
Lys 692
Leu 820
Ala 719
Val 702
Met 769
Leu 694
Tyr 777
His 781
Gly 772
Leu 768

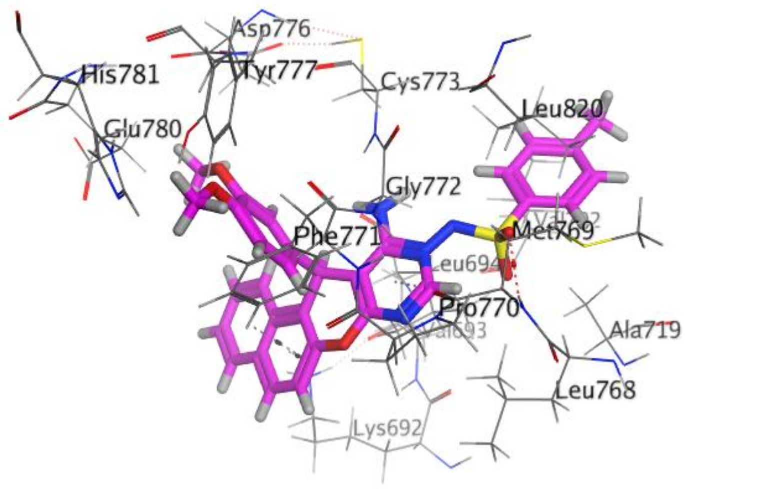

Asp776
His781
Tyr777
Cys773
Leu820
Glu780
Gly772
Met769
Phe771
Pro770
Ala719
Leu768
Lys692

<table>
<tr><td>

7

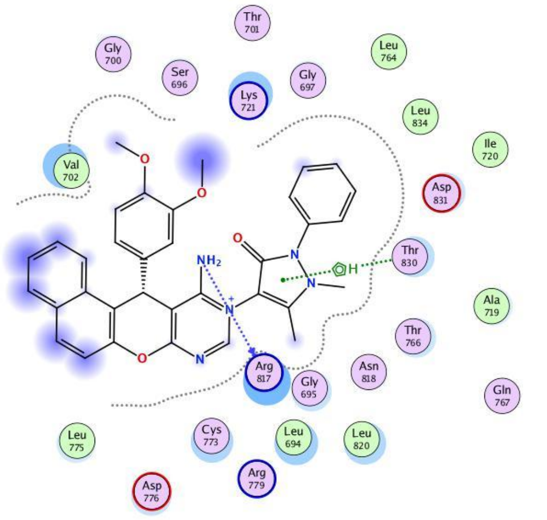

</td><td>
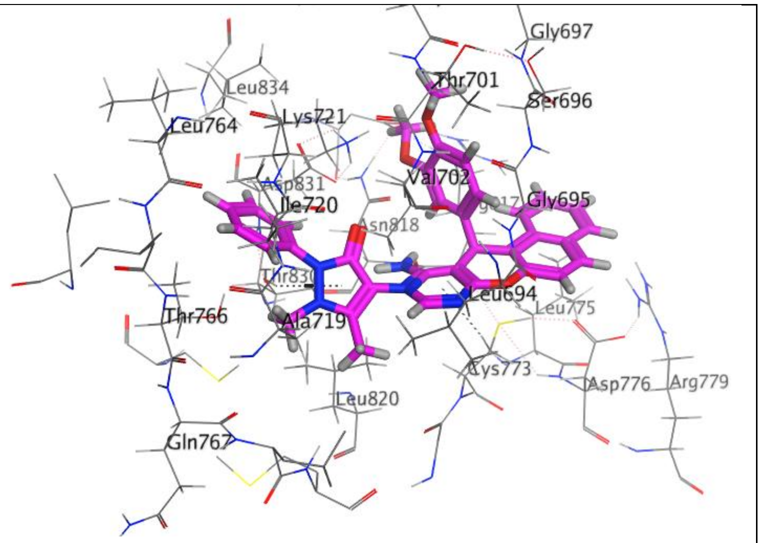

</td></tr>
<tr><td>

8

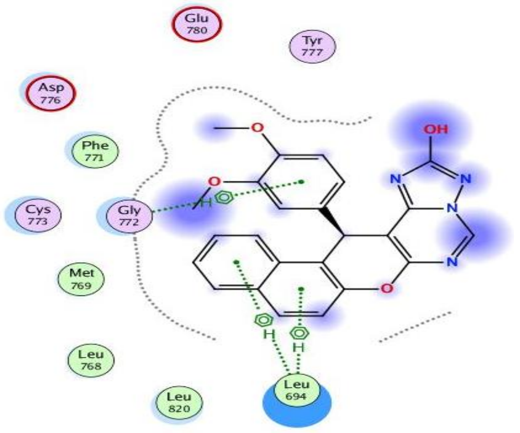

</td><td>
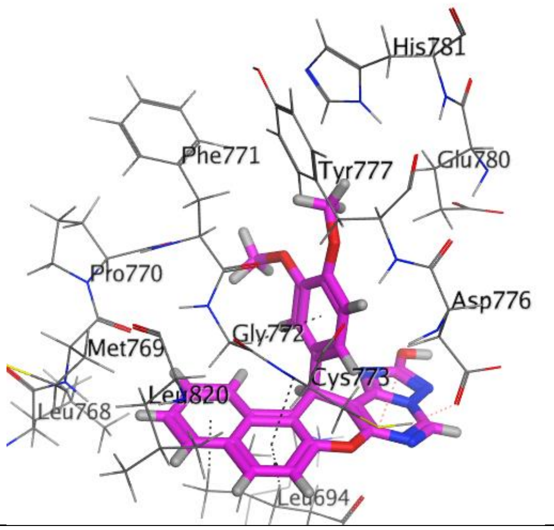

</td></tr>
<tr><td>

9

</td><td></td></tr>
</table>

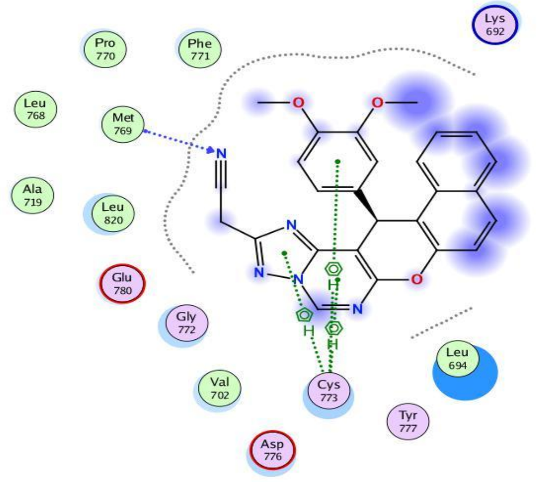

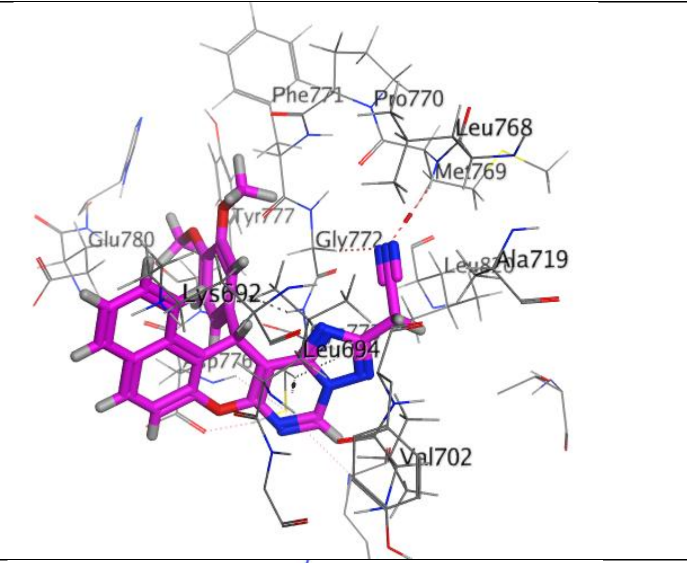

10

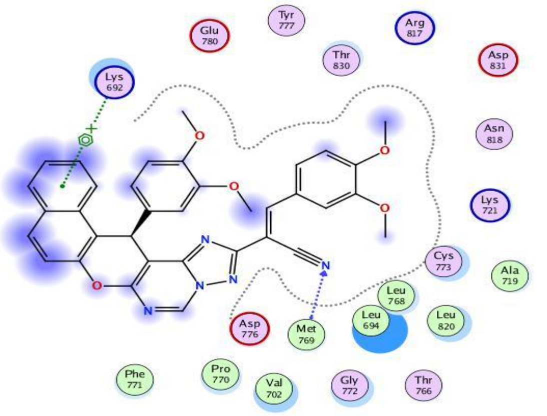

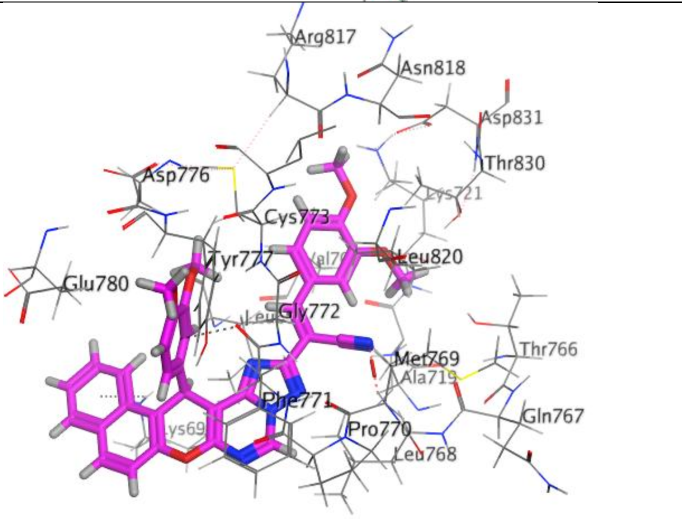

Gefitinib reference

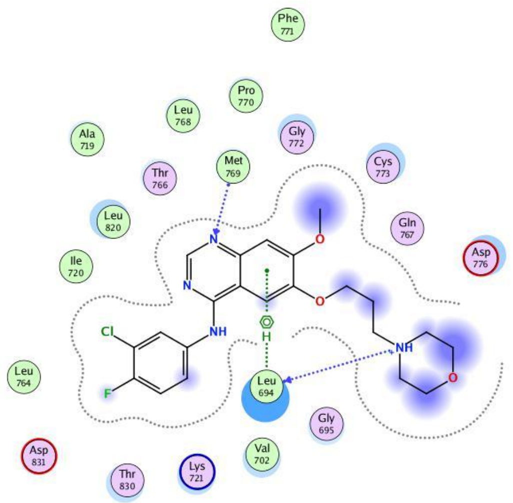

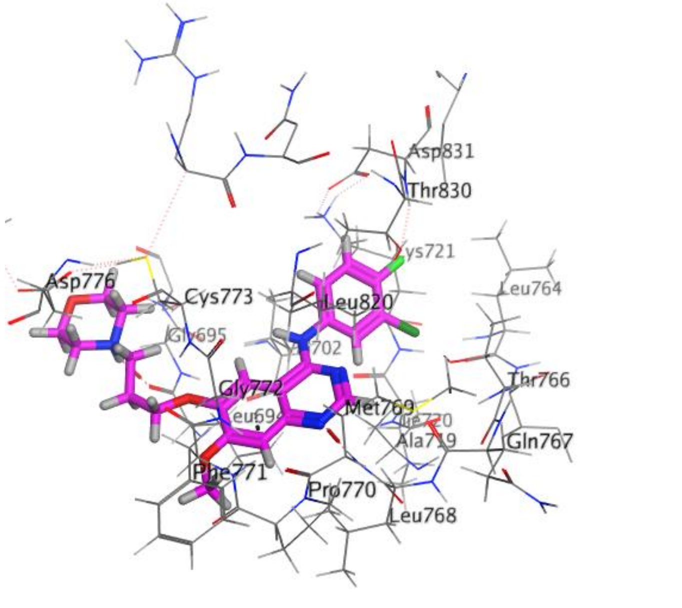

**Co-Crystallized ligand**	
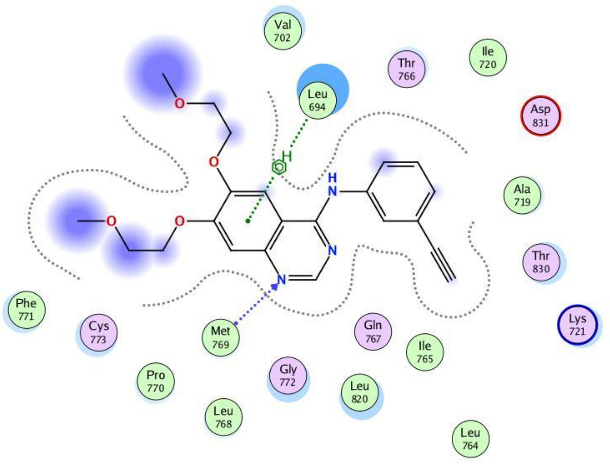	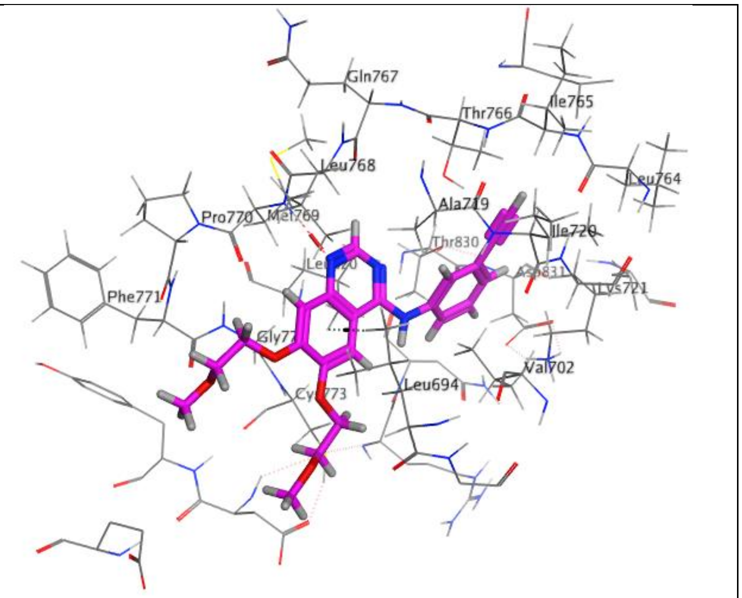
